# Humulane-Type Macrocyclic Sesquiterpenoids From the Endophytic Fungus *Penicillium* sp. of *Carica papaya*

**DOI:** 10.3389/fchem.2021.797858

**Published:** 2021-12-16

**Authors:** Fu-Run Wang, Li Yang, Fan-Dong Kong, Qing-Yun Ma, Qing-Yi Xie, You-Gen Wu, Hao-Fu Dai, Ping Chen, Na Xiao, You-Xing Zhao

**Affiliations:** ^1^ Haikou Key Laboratory for Research and Utilization of Tropical Natural Products, Institute of Tropical Bioscience and Biotechnology, CATAS, Haikou, China; ^2^ Key Laboratory for Quality Regulation of Tropical Horticultural Crops of Hainan Province, College of Horticulture, Hainan University, Haikou, China; ^3^ State Key Laboratory of Crop Biology, College of Agronomy, Shandong Agriculture University, Tai’an, Shandong, China; ^4^ Key Laboratory of Chemistry and Engineering of Forest Products, State Ethnic Affairs Commission, Guangxi Key Laboratory of Chemistry and Engineering of Forest Products, Guangxi Collaborative Innovation Center for Chemistry and Engineering of Forest Products, School of Chemistry and Chemical Engineering, Guangxi University for Nationalities, Nanning, China; ^5^ Hainan Institute for Tropical Agricultural Resources, CATAS, Haikou, China

**Keywords:** endophytic fungus, *Penicillium* sp., humulane-type sesquiterpenoid, anti-diabetic activity, cAMP accumulation

## Abstract

Three new humulane-type sesquiterpenoids, penirolide A (**1**), penirolide B (**2**), and 10-acetyl-phomanoxide (**3**), together with three known compounds aurasperone A (**4**), pughiinin A (**5**), and cyclo(l-Leu-l-Phe) (**6**) were isolated from the endophytic fungus *Penicillium* sp*.* derived from the leaves of *Carica papaya* L. Their structures including their absolute configurations were determined based on the analysis of NMR and HRESIMS spectra, NMR chemical shifts, and ECD calculations. Compounds **2**, **3**, **5**, and **6** significantly inhibited glucagon-induced hepatic glucose production, with EC_50_ values of 33.3, 36.1, 18.8, and 32.1 μM, respectively. Further study revealed that compounds **2**, **3**, **5**, and **6** inhibited hepatic glucose production by suppression of glucagon-induced cAMP accumulation.

## Introduction

Endophytic fungi, living in plants but non-pathogenic, have been proven to be promising sources of secondary metabolites with unusual structures as well as intriguing pharmacology activities, and become interesting and important resources for drug discovery (Strobel, 2003; Uzma et al., 2018; Gupta et al., 2020; Zhang et al., 2006). In recent years, increasing attention has been attracted to metabolite profiles of endophytic fungi from medicinal plants (Kaul et al., 2012). Papaya, *Carica papaya* L. (papaya), an edible and medicinal plant cultivated in tropical and subtropical regions, has been used as topical dressings for ulcer and dermatitis treatment, has gastrointestinal uses such as anti-helminthic and antibacterial activity treatments, has been used as anti-arthritis treatment, and has traditional uses for fertility control (Krishna et al., 2008; Pinnamaneni, 2017).

**FIGURE 1:**
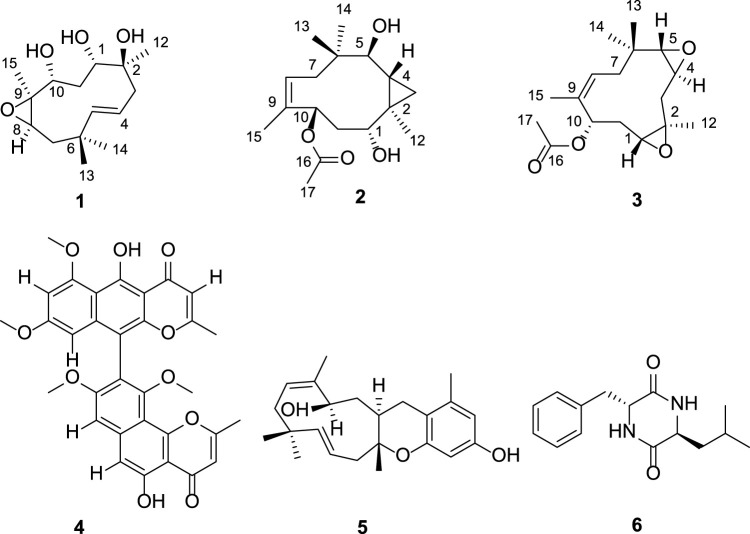
The structures of compounds **1**–**6**.

In our continuing search for structurally novel and biologically active secondary metabolites from endophytic fungi, three new humulane-type sesquiterpenoids, penirolides A (**1**) and B (**2**), and 10-acetyl-phomanoxide (**3**), were isolated from the endophytic fungus *Penicillium* sp. derived from the leaves of papaya. Humulane-type sesquiterpenoids, an uncommon type of compounds featuring an 11-membered macrocycle, were found in plants, liverworts, and fungi, and exhibited various bioactivities including antibacterial, antifungal, cytotoxic, and immunosuppressive activities (Liao et al., 2013; Luo et al., 2006; Toyota et al., 2004). Due to the flexible 11-membered macrocycle in the molecule, elucidation of the stereochemistry of humulane-type sesquiterpenoids is very challenging. Chiral derivatization and chemical conversions were successfully applied to clarify their configurations (Liao et al., 2013). However, limited amounts of sample available hampers broad use of these methods in the structural elucidation of natural products. Quantum calculations of NMR shifts represents a simple, useful, and fast alternative in address complex stereochemical problems by comparing experimental and computed values using parameters (Lodewyk et al., 2012; Grimblat and Sarotti, 2016), such as correlation coefficient, mean absolute error (MAE), corrected mean absolute error (CMAE), CP3 parameter (Smith and Goodman, 2009), DP4 probability (Smith and Goodman, 2010), or its improved version, DP4+ probability (Grimblat et al., 2015), avoiding chemical derivatization. In our effort to determine the configurations of the new humulane-type sesquiterpenoids (**1**–**3**), extensive spectroscopic analysis including 1D and 2D NMR spectra, NMR chemical shift calculations coupled with DP4+ probability analysis, and ECD calculations were utilized. In addition, all the isolates were evaluated for their anti-diabetic activity on a glucagon-induced glucose production model in mouse hepatocytes. Herein, the isolation, structural elucidation, and biological activities of compounds **1**–**6** Figure 1 were reported.

## Results and Discussion

**FIGURE 2:**
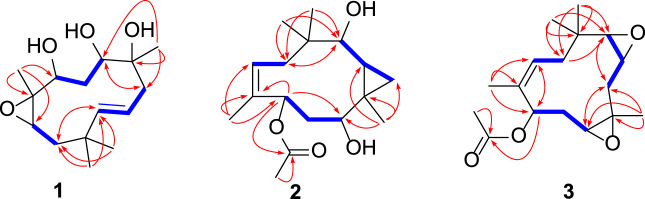
Key ^1^H-^1^H COSY (blue bold lines) and HMBC (red arrows) correlations of compounds **1**–**3**.

Compound **1** was isolated as yellow oil. Its molecular formula was determined as C_15_H_26_O_4_ from the positive ion peak at *m*/*z* 293.1723 [M + Na]^+^ (calcd for C_15_H_26_NaO_4_, 293.1723), requiring three indices of hydrogen deficiency. The ^1^H NMR spectrum (Table 1) showed signals for two olefinic protons (*δ*_H_ 5.47 and 5.43), three methylenes (*δ*_H_ 1.27–2.28), three oxygenated methines (*δ*_H_ 2.93, 3.43, and 3.44), and four methyls (*δ*_H_ 1.06, 1.08, 1.11, and 1.24). The ^13^C NMR spectrum revealed the presence of 15 carbons, which were classified by HSQC spectrum as one double bond (*δ*_C_ 122.8 and 144.7), three methylenes (*δ*_C_ 38.1, 45.1, and 45.6), three oxygenated methines (*δ*_C_ 75.6, 80.3, and 63.7), three quaternary carbons (*δ*_C_ 36.1, 67.1, and 75.6) including two oxygenated, and four methyls (*δ*_C_ 16.5, 22.3, 26.7, and 28.5). The ^1^H−^1^H COSY spectrum of **1** presented three coupling spin systems, H-7*α*/H-7*β*−H-8, H-3*α*/H-3*β*−H-4−H-5, and H-10−H-11α/H-11β−H-1 (Figure 2). The HMBC correlations (Figure 2) from CH_3_-13 (*δ*_H_ 1.06, s)/CH_3_-14 (1.11, s) to C-5 (*δ*_C_ 144.7), C-6 (*δ*_C_ 36.1), and C-7 (*δ*_C_ 45.1), from H-7*α* (*δ*_H_ 1.91, s)/H-7*β* (*δ*_H_ 1.27, s) to C-5, from CH_3_-12 (1.08, s) to C-1 (*δ*_C_ 80.3), C-2 (*δ*_C_ 75.6), and C-3 (*δ*_C_ 45.6), from H-3*α* (*δ*_H_ 2.20, s)/H-3*β* (*δ*_H_ 2.28, s) to C-1, and from CH_3_-15 (1.24, s) to C-8 (*δ*_C_ 63.7), C-9 (*δ*_C_ 67.1), and C-10 (*δ*_C_ 75.6) connected the three coupling spin systems and formed the Humulane-type sesquiterpenoid skeleton. The olefinic bond and the macrocycle accounted for two indices of hydrogen deficiency. The remaining one degree of unsaturation together with the analysis of the chemical shift of C-8 and C-9 suggested the existence of oxirane ring at positions 8 and 9.

**TABLE 1:** ^1^H (500 MHz) and ^13^C NMR (125 MHz) data for compounds **1**–**3** (*δ* in ppm, *J* in Hz).

No	1^a^		2^b^		3^b^	
	*δ*_H_	*δ*_C_	*δ*_H_	*δ*_C_	*δ*_H_	*δ*_C_
1	3.43, t (7.7)	80.3, CH	3.04, dd (11.1, 4.5)	78.9, CH	2.71, dd (13.3, 3.0)	59.7, CH
2		75.6, C		25.8, C		59.6, C
3*α*	2.20, dd (14.0, 9.5)	45.6, CH_2_	0.53, dd (9.9, 4.4)	14.6, CH_2_	2.66, dd, (12.3,3.2)	42.9, CH_2_
3*β*	2.28, dd (14.0, 4.4)		0.38, dd (5.7, 4.4)		0.63, dd (12.3, 10.5)	
4	5.47, ddd (15.8, 9.6, 4.3)	122.8, CH	1.01, dt (10.0, 5.7)	24.5, CH	2.76, ddd (10.2, 3.6, 2.4)	52.6, CH
5	5.43, dd (15.8, 1.1)	144.7, CH	2.93, d (6.1)	72.7, CH	2.33, d (2.4)	65.9, CH
6		36.1, C		39.4, C		34.4, C
7*α*	1.91, dd (13.7, 3.0)	45.1, CH_2_	1.76, ddd (14.6, 4.4, 2.3)	38.9, CH_2_	1.92, ddd (14.8, 4.3, 2.2)	38.7, CH_2_
7*β*	1.27, dd (13.7, 11.4)		2.58, t (13.6)		2.62, m	
8	2.93 dd (11.4, 3.0)	63.7, CH	5.46, dd (12.6, 3.3)	126.8, CH	5.44, dd (12.5, 3.3)	127.7, CH
9		67.1, C		132.2, C		132.6, C
10	3.44, t (5.6)	75.6, CH	5.61, d (10.4)	68.6, CH	5.86, dd (12.0, 3.0)	68.7, CH
11*α*	2.01, dt (15.6, 1.6)	38.1, CH_2_	2.25, dt (14.0, 10.8)	36.4, CH_2_	1.78, m	31.1, CH_2_
11*β*	1.22, ddd (15.6, 8.2, 5.9)		1.83, dd (14.0, 4.5)		2.26, dt (14.0, 3.0)	
12	1.08, s	22.3, CH_3_	0.87, s	12.6, CH_3_	1.27, s	18.2, CH_3_
13	1.06, s	28.5, CH_3_	0.98, s	21.9, CH_3_	1.12, s	29.7, CH_3_
14	1.11, s	26.7, CH_3_	1.15, s	25.9, CH_3_	0.77, s	17.9, CH_3_
15	1.24, s	16.5, CH_3_	1.81, brs	18.4, CH_3_	1.71, brs	18.7, CH_3_
16				170.3, C		170.1, C
17			2.01, s	21.5, CH_3_	2.05, s	21.3, CH_3_

a In MeOD.

b In CDCl_3_.

**FIGURE 3:**
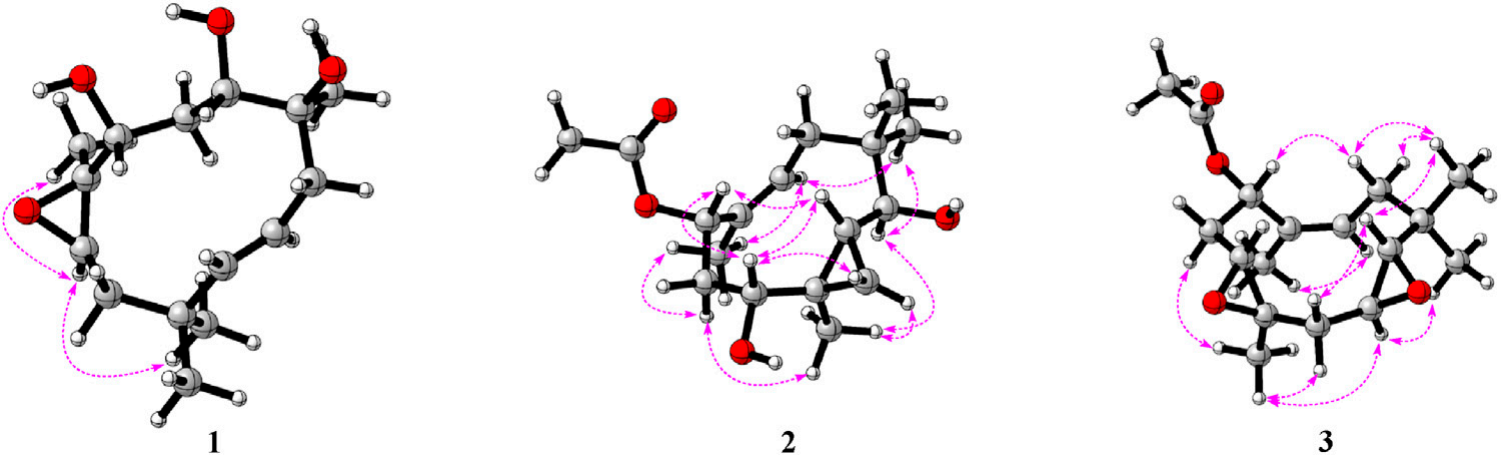
Key ROESY correlations of compounds **1**–**3**.

**FIGURE 4:**
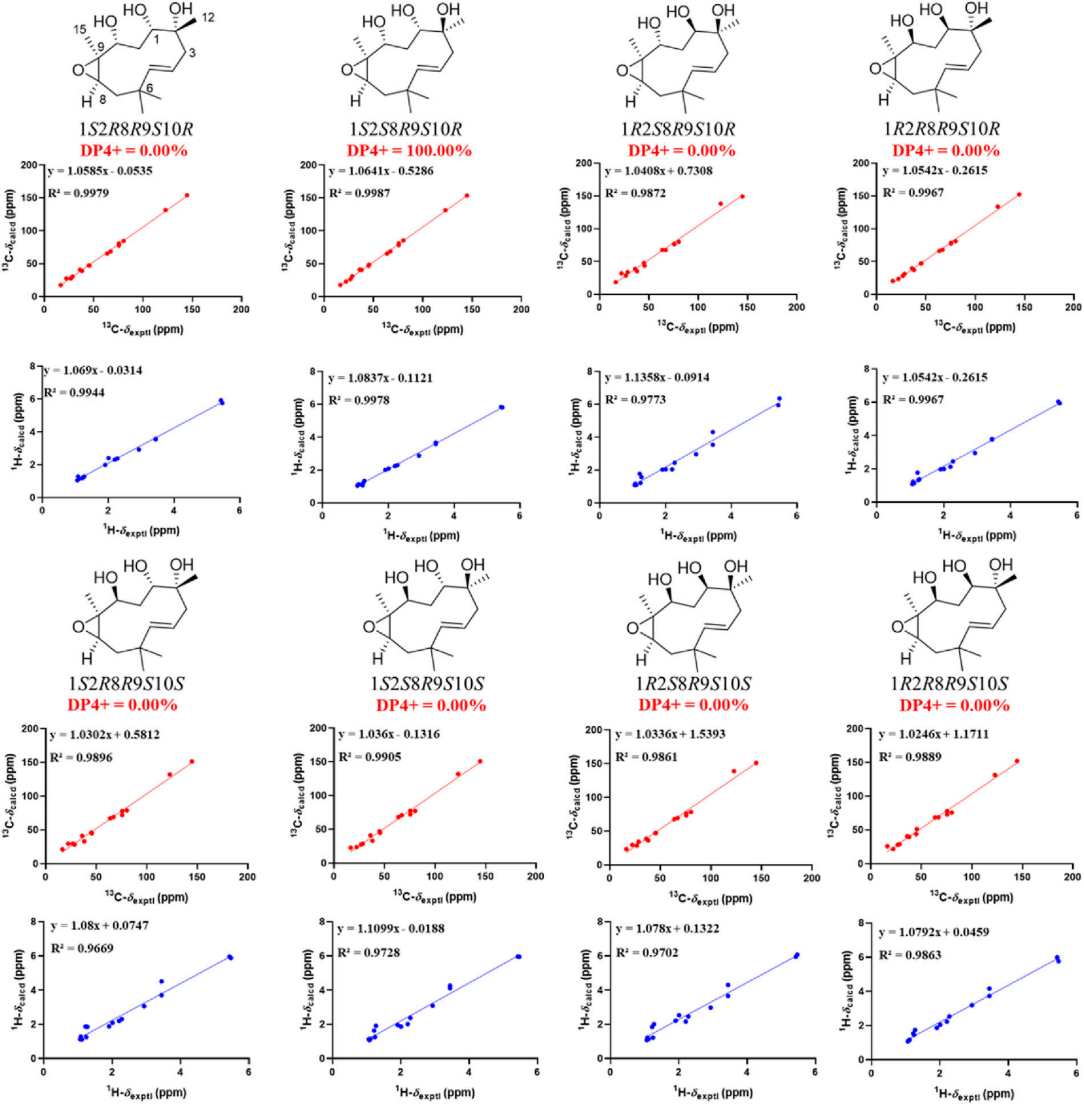
Linear regression analysis between experimental and calculated ^13^C and ^1^H NMR chemical shifts of isomers of **1**.

**FIGURE 5:**
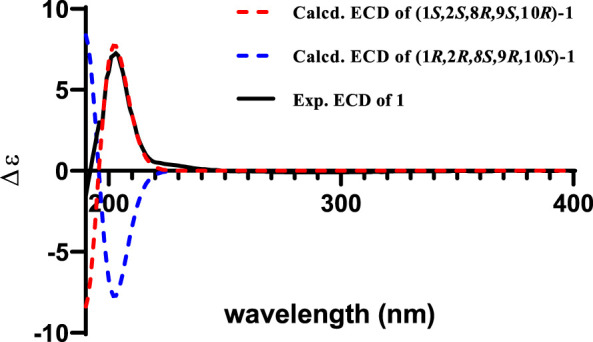
Experimental spectrum of **1** in methanol and calculated ECD spectra of (1*S*,2*S*,8*R*,9*S*,10*R*)-**1** and (1*R*,2*R*,8*S*,9*R*,10*S*)-**1**.

The vicinal coupling constant (*J* = 15.8 Hz) between H-4 and H-5 assigned the *E* configured *Δ* ^4^ double bond. In the ROESY spectrum, the key correlation of CH_3_-15 with H-8 (*δ*_H_ 2.93) indicated that CH_3_-15 and H-8 were cofacial (Figure 3). However, due to the flexibility of the 11-membered ring and the signals overlap (e.g., H-1 and H-10), the relative configuration of other stereocenters (C-1, C-2, and C-10) on the ring could not be fully determined by ROESY spectrum. Thus, we performed theoretical NMR chemical shifts calculations of eight diastereomers (Figure 4) of **1** at mPW1PW91-SCRF/6-311G(d,p)//B3LYP-D3BJ/6-31G(d) theoretical level in methanol with the GIAO method (Wolinski et al., 1990). The calculated ^13^C and ^1^H NMR chemical shifts of (1S*,2S*,8R*,9S*,10R*)-**1** showed the best agreement with the experimental values. Furthermore, DP4+ analysis (Grimblat et al., 2015) predicted that (1S*,2S*,8R*,9S*,10R*)-**1** was the most likely candidates with 100% probability. The absolute configuration of **1** was assigned by the ECD calculation (Figure 5). The calculated ECD curve of (1*S*,2*S*,8*R*,9*S*,10*R*)-**1** matched well with the experimental curve, establishing the absolute configuration of **1** as 1*S*,2*S*,8*R*,9*S*,10*R*. Collectively, compound **1** was identified as shown in Figure 1 and named penirolide A.

Compound **2** was isolated as yellow oil. Its molecular formula was determined as C_17_H_28_O_4_ from the positive ion at *m*/*z* 319.1882 [M + Na]^+^ (calcd for C_17_H_28_NaO_4_, 319.1880) in the HRESIMS, corresponding to four degrees of unsaturation. The ^1^H NMR spectrum (Table 1) showed resonances for one olefinic proton (*δ*_H_ 5.61), three sp^3^ methines (*δ*_H_ 1.01, 2.93, and 3.04), five methyls (*δ*_H_ 0.87, 0.98, 1.15, 1.81, and 2.01), and three methylenes in which one methylene has exceptionally upfield chemical shifts (*δ*_H_ 0.53 and 0.38), suggesting the existence of a cyclopropane unit. The ^13^C NMR spectrum (Table 1) revealed the presence of 17 carbons comprising five methyls, three methylenes, five methines (including one olefinic and three oxygenated), and four non-protonated carbons (including one olefinic and one carbonyl). Three coupling spin systems, H-1−H-11*α*/H-11*β*, H-3*α*/H-3*β*−H-4−H-5, and H-7*α*/H-7*β*−H-8, could be deduced from the ^1^H-^1^H COSY correlations (Figure 2). In the HMBC spectrum, correlations of CH_3_-12 (*δ*_H_ 0.87, s) with C-2 (*δ*_C_ 25.8), C-3 (*δ*_C_ 14.6), and C-4 (*δ*_C_ 24.5) indicated the presence of C-2−C-3−C-4 cyclopropane moiety, which connected with C-1 as established by the correlations from H-1 (*δ*_H_ 3.04) to C-2 and C-3. The HMBC correlations (Figure 2) from two methyls, CH_3_-13 (*δ*_H_ 1.15) and CH_3_-14 (*δ*_H_ 0.98), to C-5 (*δ*_C_ 72.7), C-6 (*δ*_C_ 39.4), and C-7 (*δ*_C_ 38.9), from H-5 (*δ*_H_ 2.93) to C-7 (*δ*_C_ 38.9), and from CH_3_-15 (*δ*_H_ 1.81) to C-8 (*δ*_C_ 126.8), C-9 (*δ*_C_ 132.2), and C-10 (*δ*_C_ 68.6) constructed a 10-membered ring with two geminal methyls at C-6 and one methyl at C-9, which fused with the above noted cyclopropane ring through C-2/C-3. Additional HMBC correlations from CH_3_-17 (*δ*_H_ 2.01) and H-10 (*δ*_H_ 5.61) to carbonyl C-16 (*δ*_C_ 170.3) enable attachment of an acetyl group to the C-10.

**FIGURE 6:**
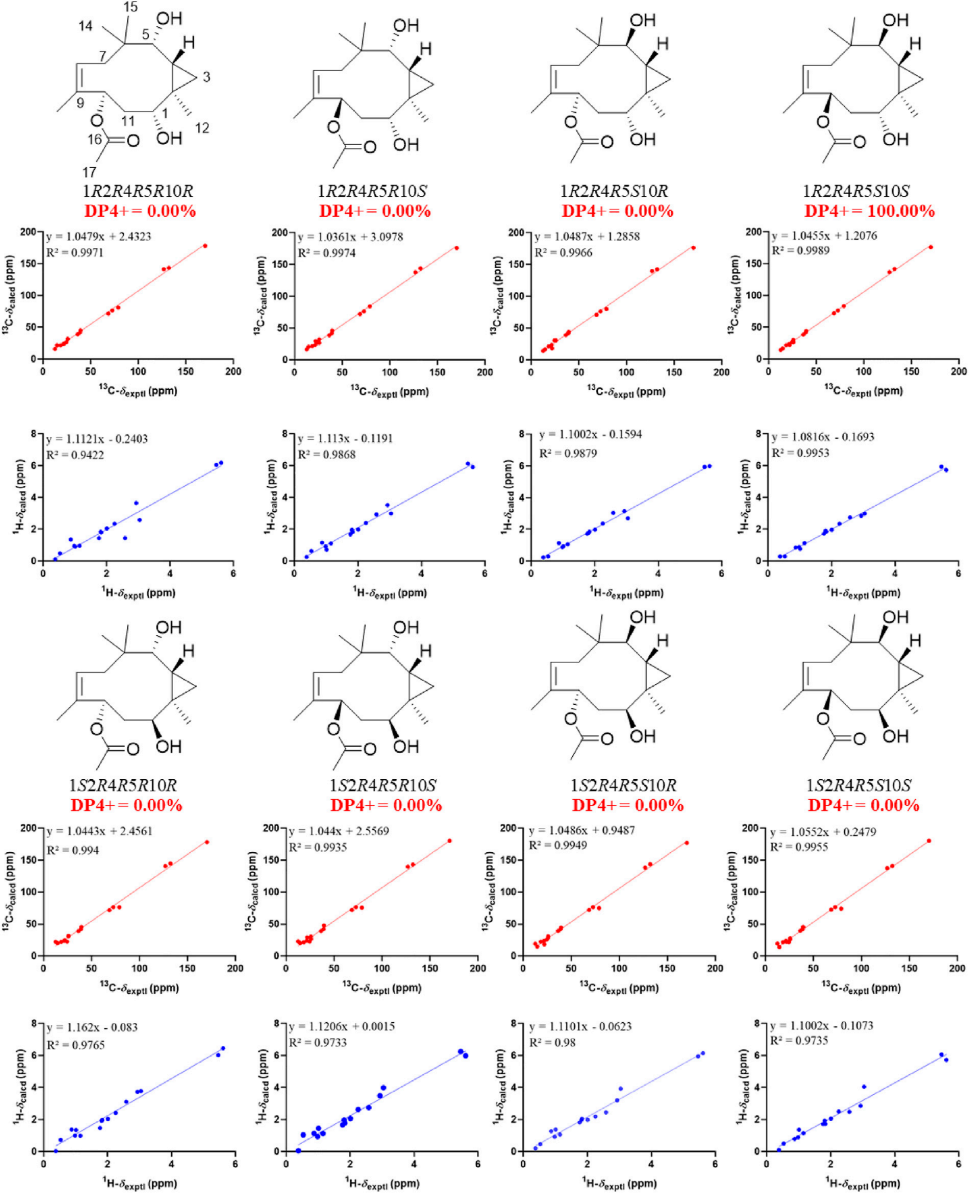
Linear regression analysis between experimental and calculated ^13^C and ^1^H NMR chemical shifts of isomers of **2**.

**FIGURE 7:**
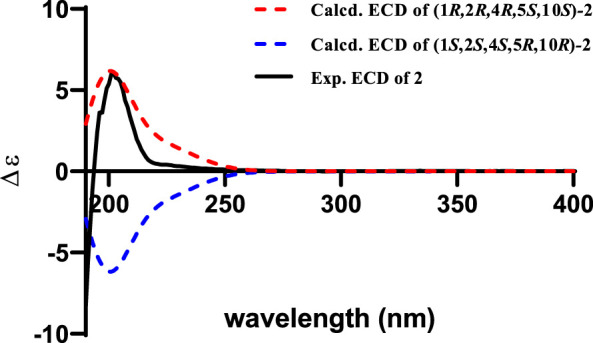
Experimental spectrum of **2** in methanol and calculated ECD spectra of (1*R*,2*R*,4*R*,5*S*,10*S*)-**2** and (1*S*,2*S*,4*S*,5*R*,10*R*)-**2**.

The relative configuration of **2** was initially assigned by ROESY correlations. The ROESY correlations (Figure 3) of CH_3_-15 with H-8 (*δ*_H_ 5.46) and H-10 with H-7α (*δ*_H_ 1.76)/H-7β (*δ*_H_ 2.58) indicated *Z* geometry for the *Δ* ^8^ double bond. The sequential ROESY correlations of H-10/H-4/H-1/H-3*β* placed these protons on the same face the ring. The CH_3_-12 showed ROESY correlations with H-3*α* and H-5, suggesting that they were located on the face opposite to H-4. However, relative configuration assignment only based on ROESY correlations is usually not reliable enough in conformationally flexible molecules such as macrocycles. In order to irrefutably determine the relative configuration of the chiral centers (C-1, C-5, and C-10) on the macrocycle of **2**, eight possible diastereomers of **2** were applied to theoretical calculations of NMR chemical shifts followed by DP4+ analysis (Figure 6). The calculated chemical shifts of (1R*,2R*,4R*,5S*,10S*)-**2** showed best agreement with the experimental values among the possible diastereomers and (1R*,2R*,4R*,5S*,10S*)*-* **2** possessed 100% DP4+ probability (Grimblat et al., 2015), indicating that (1R*,2R*,4R*,5S*,10S*)*-* **2** was the most likely candidate structure. The ECD calculation was further employed to clarify the absolute configuration of **2**. The calculated ECD curve of (1*R*,2*R*,4*R*,5*S*,10*S*)*-* **2** well fitted with the experimental one (Figure 7), defining the stereochemistry of **2** as 1*R*,2*R*,4*R*,5*S*,10*S*. Accordingly, compound **2** was elucidated as shown in Figure 1 and named penirolide B.

Compound **3** was obtained as yellow oil and its molecular formula of C_17_H_26_O_4_ was assigned by HRESIMS positive ion peak at 317.1723 [M + Na]^+^ (calcd for C_17_H_26_NaO_4_, 317.1723), indicating five degrees of unsaturation. The ^1^H NMR spectrum (Table 1) displayed signals for one olefinic proton (*δ*_H_ 5.44), four sp^3^ methines (*δ*_H_ 2.71, 2.76, 2.33, and 5.86), five methyls (*δ*_H_ 0.77, 1.12, 1.27, 1.71, and 2.05), and three methylenes. The ^13^C NMR spectrum (Table 1) showed resonances for 17 carbons ascribed by HSQC spectrum as five methyls, three methylenes, five methines (including one olefinic and three oxygenated), and four non-protonated carbons (including one olefinic and one carbonyl). These NMR data were very similar to that of phomanoxide (Zhang et al., 2015) whose structure was confirmed by x-ray crystallography, with the only difference being the presence of an additional acetyl group [*δ*_H_ 2.05 (s, 3H), *δ*_C_ 21.3, CH_3_-17; *δ*_C_ 170.1, C-16] in **3**. The deshielded shift of C-10 (*δ*_C_ 68.7; *Δδ*_C_ 2.1) in **3** relative to that of phomanoxide indicated that the acetyl attached at C-10, which was supported by the HMBC correlations from CH_3_-17 and H-10 (*δ*_H_ 5.86) to carbonyl C-16 (Figure 2).

**FIGURE 8:**
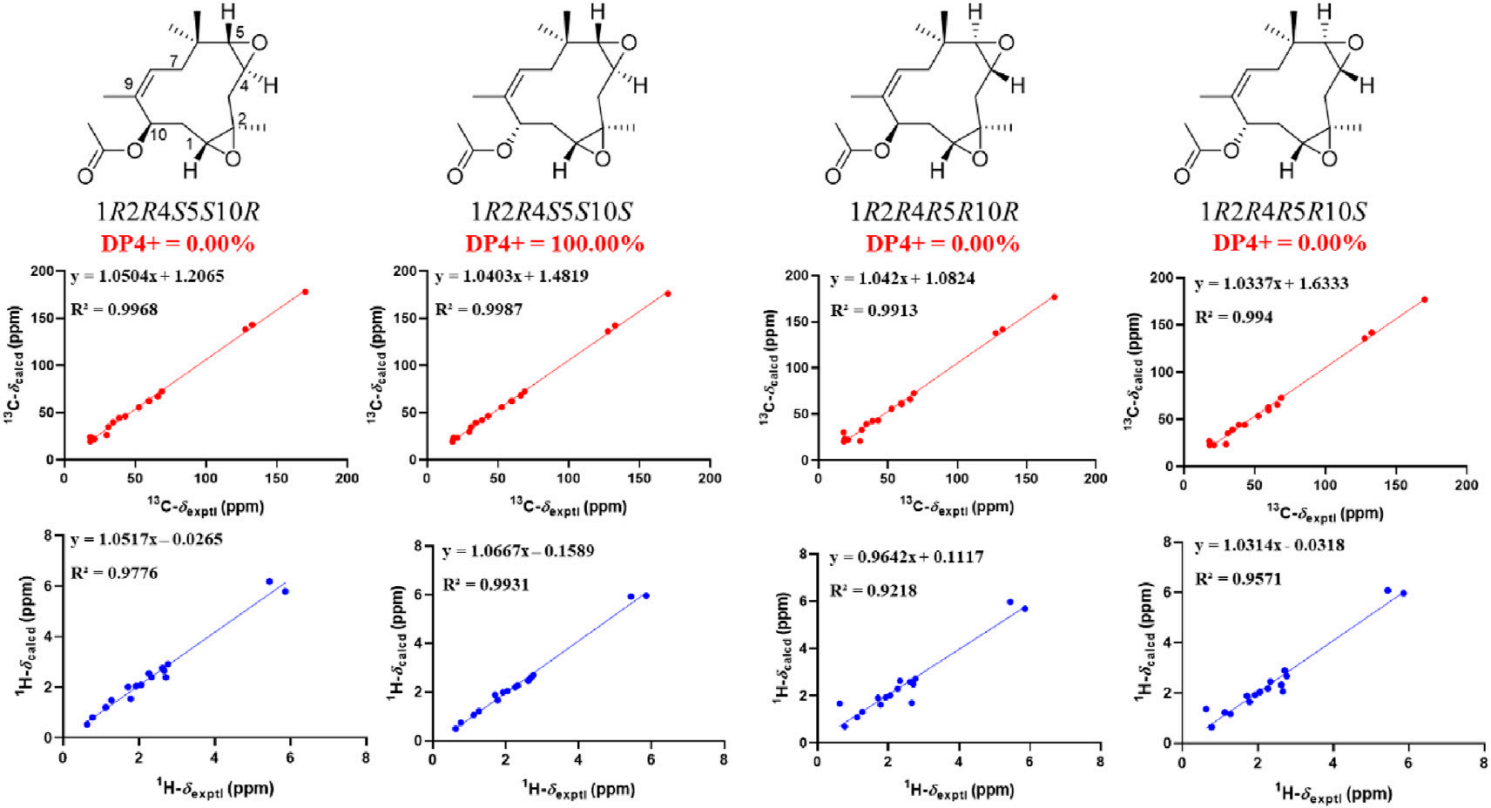
Linear regression analysis between experimental and calculated ^13^C and ^1^H NMR chemical shifts of isomers of **3**.

**FIGURE 9:**
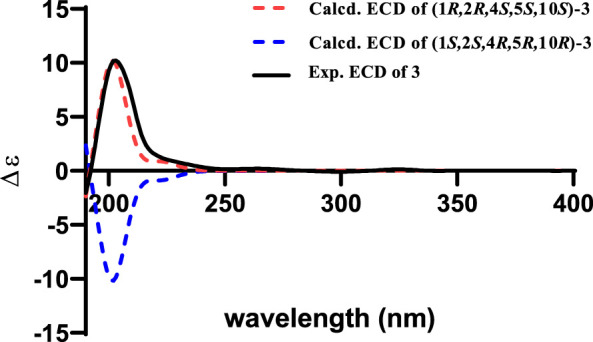
Experimental spectrum of **3** in methanol and calculated ECD spectra of (1*R*,2*R*,4*S*,5*S*,10*S*)-**3** and (1*S*,2*S*,4*R*,5*R*,10*R*)-**3**.

In the ROESY spectrum of **3**, the correlations of CH_3_-12 with H-3*α*, H-4, and H-11*α* suggested that these protons resided on the same face of the molecule. Cross peak of H-3*β*/H-5 indicated that the two protons were cofacial (Figure 3). The large coupling constant *J*_H-10−H-11*α*_ = 12.0 Hz demonstrated that H-10 and H-11*α* were *trans* oriented. Therefore, the relative configuration was proposed as 1R*,2R*,4S*,5S*,10S*, which was further confirmed by the NMR chemical shift calculation (Figure 8) and its similar NMR data to that of phomanoxide. The 1*R*,2*R*,4*S*,5*S*,10*S* configuration for **3** was established based on its well-matched experimental and calculated ECD curves (Figure 9). Thus, the structure of **3** was elucidated as depicted in Figure 1 and named 10-acetyl-phomanoxide.

In addition, three known compounds, aurasperone A (**4**) (Campos et al., 2005), pughiinin A (**5**) (Pittayakhajonwut et al., 2002), and cyclo(l-Leu-l-Phe) (**6**) (Stark and Hofmann, 2005), were identified by comparison of their NMR data with literature data.

**FIGURE 10:**
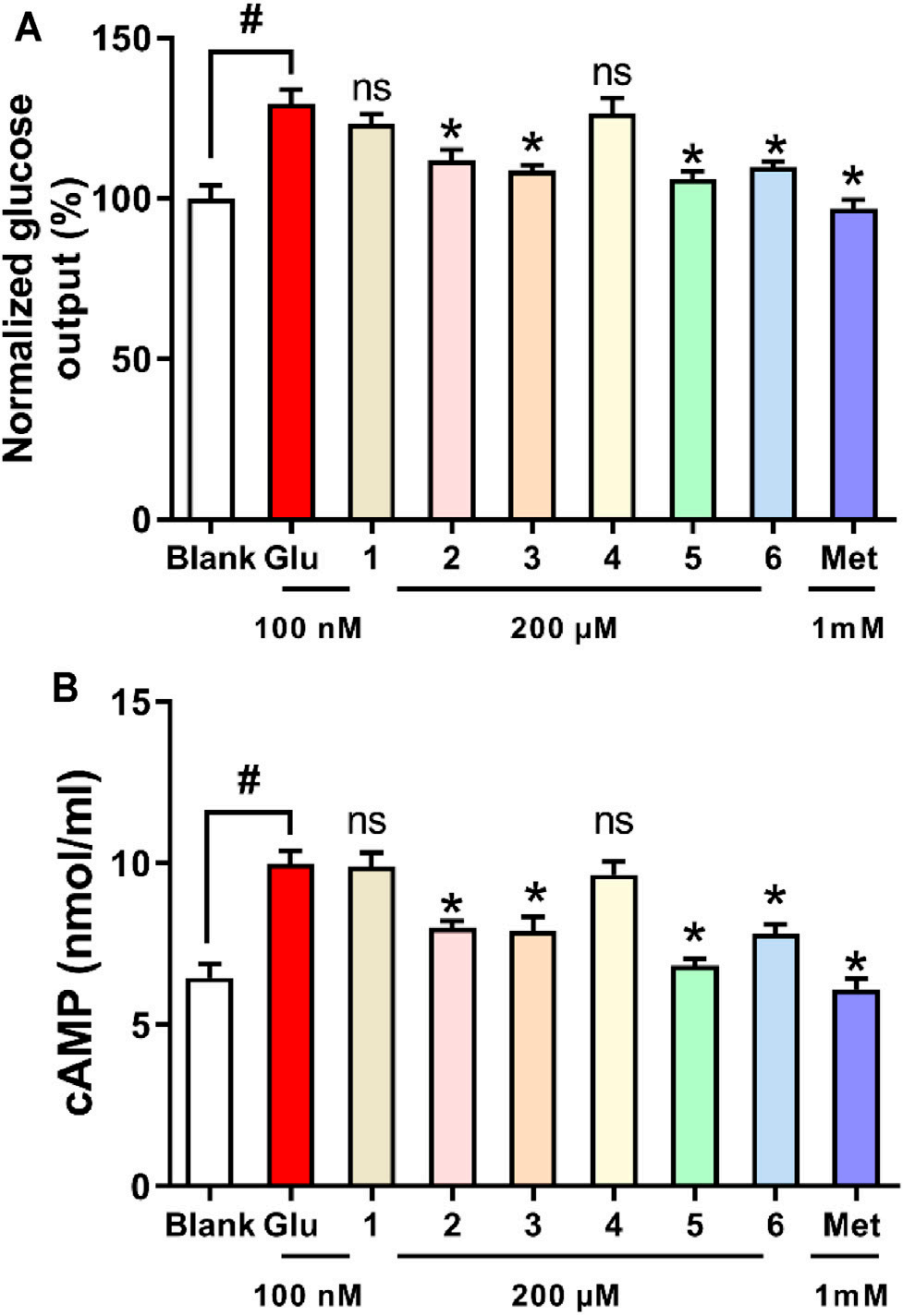
Effects of compounds **1**–**6** on glucagon-induced hepatic glucose production **(A)** and cAMP accumulation **(B)** in mouse hepatocytes. *p < 0.05 and #*p* < 0.05 as compared with the blank group and group treated with glucagon, respectively.

The liver plays a major role in whole body glucose metabolism by maintaining a balance between glucose production and glucose storage. Excessive hepatic glucose production contributes substantially to diabetes, and it is proposed that suppression of hepatic glucose production may provide therapeutic advantages for the control of diabetes (Xiao et al., 2017; Liao et al., 2021). To investigate the anti-diabetic effect of the six compounds, we examined the glucose production in hepatocytes. Compounds **2**, **3**, **5**, and **6** significantly inhibited glucagon-induced hepatic glucose production, with EC_50_ values of 33.3, 36.1, 18.8, and 32.1 μM, respectively, while it was >200 µM for Compounds **1** and **4**, and 2.3 μM for the positive control metformin (Figure 10A). In response to glucagon, cAMP is a second messenger to initiate glucagon signaling cascades in hepatic glucose production. Compounds **2**, **3**, **5**, and **6** treatment suppressed cAMP accumulation (Figure 10B). These results indicated that compounds **2, 3, 5,** and **6** inhibited hepatic glucose production by suppression glucagon-induced cAMP accumulation.

## Materials and Methods

### General Experimental Procedures

Optical rotations were measured with a JASCO P-1020 digital polarimeter. The infrared spectra were recorded on a Shimadzu UV2550 spectrophotometer (Shimadzu, Kyoto, Japan). The mass spectrometric (HRESIMS) data were acquired using an API QSTAR Pulsar mass spectrometer (Bruker, Bremen, Germany). Semipreparative high-performance liquid chromatography (HPLC) equipped with octadecyl silane (ODS) column (Cosmosil ODS-A, 10 × 250 nm, 5 μm, 4 ml/min) was used to isolate compounds. The solvents used to the preparative HPLC, such as methanol, acetonitrile, hexane, and ethanol were of chromatographic grade (Concord Technology Co. Ltd., Tianjin, China). The solvents used to the extraction or isolation of the columns, such as ethyl acetate, methanol, chloroform, and methanol, were of analytical pure (Concord Technology Co. Ltd., Tianjin, China). The NMR spectra were recorded with a Bruker AV-500 spectrometer (Bruker, Bremen, Germany) using TMS as an internal standard. Silica gel (60–80 and 200–300 mesh; Qingdao Haiyang Chemical Co. Ltd., Qingdao, China) and Rp-C18 (20–45 μm; Fuji Silysia Chemical Ltd., Durham, NC, United States) were used for column chromatography.

### Fungal Material

The fungus *Penicillium* sp. was isolated from healthy papaya leaves collected in Haikou, Hainan Province, People’s Republic of China, and identified by sequence analysis of the ITS region of rDNA (GenBank No. MT729953). A voucher strain was deposited in the Institute of Tropical Bioscience and Biotechnology.

### Fermentation and Isolation

Plugs of agar with mycelium were cut from solid medium and transferred aseptically to a 1,000-ml Erlenmeyer flask, containing 300 ml of liquid medium (glucose 10 g/L, maltose 20 g/L, monosodium glutamate 10 g/L, yeast extract 3 g/L, corn starch 1 g/L, mannitol 20 g/L, MgSO_4_ 0.3 g/L, and KH_2_PO_4_ 0.5 g/L). The whole culture broth (45 L) was harvested and filtered to yield the mycelium cake and liquid broth. The mycelium cake and liquid broth were extracted by EtOAc three times. The two EtOAc extracts were evaporated under reduced pressure and combined based on their similar metabolite profiles provided by HPLC analysis, affording a total of 12 g of EtOAc extract. The extract was separated by silica gel column eluted with different ratios of Petroleum ether-EtOAc (8:1, 6:1, 4:1, 2:1, 1:1, and 0:1) to afford six fractions (Fr.1–6). Fr.4 (0.52 g) was purified by a Rp-C_18_ silica gel column (MeOH-H_2_O, 70%–30%), followed by semi-preparative HPLC (MeCN-H_2_O, containing 0.1% Formic acid, 50:50, v/v, 4.0 ml min^−1^) to obtain compounds **1** (7.0 mg, *t*_R_ = 9.5 min) and **2** (4.6 mg, *t*_R_ = 6.5 min). Fr.3 (1.03 g) was applied to a Rp-C_18_ silica gel column chromatography (MeOH-H_2_O, 50%–50%) and semi-preparative HPLC (MeCN-H_2_O, containing 0.1% Formic acid, 20:80, v/v, 4.0 ml min^−1^) to obtain compound **3** (4.8 mg, *t*_R_ = 9.6 min). Fr.6 (1.01 g) was subjected to a Rp-C_18_ silica gel column chromatography (MeOH-H_2_O, 80‒20%) to obtain three subfractions (Fr.6.1–Fr.6.3), Compounds **4** (18.3 mg, *t*_R_ = 8.3 min) and **5** (18.2 mg, *t*_R_ = 7.5 min) were obtained from Fr.6.1 by semi-preparative HPLC (MeCN-H_2_O, containing 0.1% formic acid, 75:25, v/v, 4.0 ml min^−1^). Fr.6.3 (0.33 g) was subjected to semi-preparative HPLC (MeCN-H_2_O, containing 0.1% formic acid, 15:85, v/v, 4.0 ml min^−1^) to afford **6** (3.2 mg, *t*_R_ = 10.5 min).

### Penirolide A (1)

Yellow oily; [ɑ] 25D −9.0 (*c* 0.1, MeOH); UV (CH_3_OH) *λ*_max_ (log*ε*): 206 (2.52) nm; ECD (CH_3_OH) *λ*_max_ (∆*ε*): 203 (7.25) nm; IR(KBr) *ν*_max_: 3,414, 2,962, 2,872, 1,727, 1,668, 1,453,1,384, 1,285, 1,200, 1,091, 1,065, 990 cm^−1^; ^1^H and ^13^C NMR spectral data, Table 1; HRESIMS *m*/*z* 293.1723 ([M + Na]^+^ (calcd for C_15_H_26_NaO_4_, 293.1723).

### Penirolide B (2)

Yellow oily; [ɑ] 25D +3.0 (*c* 0.1, MeOH); UV (CH_3_OH) *λ*_max_ (log*ε*): 205 (2.56) nm; ECD (CH_3_OH) *λ*_max_ (∆*ε*): 202 (8.09) nm; IR(KBr) *ν*_max_: 3,422, 2,960, 1,728, 1,453, 1,373, 1,248, 1,050, 960 cm^−1^; ^1^H and ^13^C NMR spectral data, Table 1; HRESIMS *m*/*z* 319.1882 [M + Na]^+^ (calcd for C_17_H_28_NaO_4_, 319.1880).

### 10-Acetyl-phomanoxide (3)

Yellow oily; [ɑ] 25D +31.9 (*c* 0.1, MeOH); UV (CH_3_OH) *λ*_max_ (log*ε*): 205 (2.55) nm; ECD (CH_3_OH) *λ*_max_ (∆*ε*): 203 (10.12) nm; IR(KBr) *ν*_max_: 3,442, 2,961, 2,929, 1,736, 1,380, 1,242, 1,024 cm^−1^; ^1^H and ^13^C NMR spectral data, Table 1; HRESIMS *m*/*z* 317.1723 [M + Na]^+^ (calcd for C_17_H_26_NaO_4_, 317.1723).

### NMR and ECD Calculations

The conformations of the isomers of compounds **1**–**3** were generated by iMTD-GC method embedded in Crest program (Pracht et al., 2020). Two conformations with the root-mean-square (RMS) distance and energy deviation of 0.5 Å and 0.25 kcal/mol, respectively, were considered as duplicates and one of them was removed. Density functional theory calculations were performed with the Gaussian 16 package (Frisch et al., 2019). The remaining conformers with population over 1% were optimized at the B3LYP-D3BJ/6-31G(d) level in gas phase and the conformers within an energy window of 3 kcal/mol were kept. Then, these conformers were refined by re-optimizations at the B3LYP-D3BJ/6-311G(d,p) level with the IEFPCM solvent model in methanol for **1** and chloroform for **2** and **3**, and frequency analysis of all optimized conformations was also performed at the same level of theory to ensure that no imaginary frequencies were present, confirming that the optimized structures were minima on their potential energy surfaces. NMR shielding tensors were calculated with the GIAO method (Wolinski et al., 1990) at the mPW1PW91/6-311G(d,p) level with the IEFPCM solvent model in methanol for **1** and chloroform for **2** and **3**. The calculated isotropic magnetic shielding constants (σ) were Boltzmann averaged according to their Gibbs free energies. The shielding constants were converted into chemical shifts by referencing to TMS at 0 ppm according to the formula *δ*cal = *σ*TMS–*σ*cal, where the *σ*TMS (the shielding constant of TMS) was calculated at the same level. For each candidate, the parameters a and b of the linear regression *δ*cal = a*δ*exp + b; the correlation coefficient, *R* ^2^; the mean absolute error (MAE) defined as Σn |*δ*cal–*δ*exp|/n; and the corrected mean absolute error, CMAE, defined as Σn |*δ*corr–*δ*exp|/n, where *δ*corr = (*δ*cal–b)/a, were calculated. DP4+ probability analysis was performed using the calculated NMR shielding tensors with DP4+ excel file (Grimblat et al., 2015). ECD spectra were calculated by the TDDFT methodology at the B3LYP/def2TZVP utilizing IEFPCM in methanol. The final ECD spectra were simulated by averaging the spectra of lowest energy conformers according to the Boltzmann distribution theory and their relative Gibbs free energy (*ΔG*) using SpecDis 1.71 (Bruhn et al., 2013) with *σ* = 0.30 eV and uv shift = 5 nm for **1** and 10 nm for **2** and **3**, respectively.

### Primary Mouse Hepatocytes

Male C57BL/6J mice (6 weeks old) were purchased from Pengyue Laboratory Animal Company (Jinan, China). Animal care and experiments were approved by the Animal Ethics Committee of Shandong Agriculture University. Primary mouse hepatocytes were prepared as previously described (Xiao et al., 2017). Briefly, fasted C57BL/6J male mice were anesthetized and livers were washed with Krebs-HEPES and digested with collagenase IV by perfusion through the inferior vena cava at 3 ml/min. Then, the whole liver was removed 6 min later, and hepatocytes were extracted in DMEM with 10% FBS. After filtering, cells were resuspended and cultured in 96- or 48-well plates. Then, the cells were treated as indicated.

### Measurement of Cell Viability and Glucose Production in Hepatocytes

Primary mouse hepatocytes were seeded in a 96-well plate and treated with 100 nM glucagon and various concentrations of test compounds (1–200 μM) for 24 h. After that, cell viability was assessed by the MTT method (Pan et al., 2021). For the determination of glucose production, hepatocytes were incubated in KRB solution containing relevant substrates (10 mM pyruvate, 100 nM glucagon) or indicated compounds (1, 5, 10, 100, and 200 μM) for 6 h. Then, the cell supernatant was collected for glucose analysis using the commercial kit.

### Measurement of cAMP Production

Hepatocytes were incubated with the indicated compounds and stimulated with glucagon (100 nM) for 2 h, lysed in cell lysis buffer, and the supernatant was harvested for the assays of cAMP (Xiao et al., 2017). All data were expressed as the mean ± SD from at least three independent experiments.

## Data Availability

The original contributions presented in the study are included in the article/Supplementary Material. Further inquiries can be directed to the corresponding authors.
