# Supplementary material for: Humulane-Type Macrocyclic Sesquiterpenoids From the Endophytic Fungus Penicillium sp. of Carica papaya
Source: Front Chem. 2021 Dec 16;9:797858. doi: 10.3389/fchem.2021.797858 (PMC8717546; doi:10.3389/fchem.2021.797858)
Supplement: Supplementary file 1 [file DataSheet1.docx]

**Supplementary Material for**

**Humulane-type** **macrocyclic sesquiterpenoids from the endophytic fungus** ***Penicillium* sp. of** ***Carica papaya* L.**

**Fu-Run Wang^1,2,┴^, Li Yang^1,┴^, Fan-Dong Kong^4,┴^, Qing-Yun Ma^1^, Qing-Yi Xie^1^, You-Gen Wu^2^, Hao-Fu Dai^5^, Ping Chen^2,*^, Na Xiao^3,*^, and You-Xing Zhao^1,*^**

*^1^Haikou Key Laboratory for Research and Utilization of Tropical Natural Products,* *Institute of Tropical Bioscience and Biotechnology, CATAS, Haikou 571101, China.*

*^2^College of Horticulture, Hainan University, Key Laboratory for Quality Regulation of Tropical Horticultural Crops of Hainan Province, Haikou 570228, China.*

*^3^State Key Laboratory of Crop Biology, College of Agronomy, Shandong Agriculture University, Tai'an, Shandong 271018, China.*

*^4^Key Laboratory of Chemistry and Engineering of Forest Products, State Ethnic Affairs Commission, Guangxi Key Laboratory of Chemistry and Engineering of Forest Products, Guangxi Collaborative Innovation Center for Chemistry and Engineering of Forest Products, School of Chemistry and Chemical Engineering, Guangxi University for Nationalities, Nanning 530006, China.*

*^5^Hainan Institute for Tropical Agricultural Resources, CATAS, Haikou 571101, China.*

^┴^These authors contributed equally to this work.

* Correspondence:

Ping Chen

chenping08213@163.com

Na Xiao

[xiaona198707@126.com](mailto:xiaona198707@126.com)

Youxing Zhao

zhaoyouxing@itbb.org.cn

**Contents**

**Table S1.** Experimental and calculated ^13^C NMR chemical shifts of **1**. 7

**Table S2.** Experimental and calculated ^1^H NMR chemical shifts of **1**. 7

**Table S3.** The results of the DP4+ analysis of **1** 8

**Table S4.** Conformational analysis of the optimized isomers of **1** at B3LYP/6-311G(d) level in methanol. 9

**Figure S1.** Optimized geometries of isomers of **1** at B3LYP/6-311G(d) level in methanol. 10

**Table S5.** Experimental and calculated ^13^C NMR chemical shifts of **2**. 11

**Table S6.** Experimental and calculated ^1^H NMR chemical shifts of **2**. 11

**Table S7.** The results of the DP4+ analysis of **2** 12

**Table S8.** Conformational analysis of the optimized isomers of **2** at B3LYP/6-311G(d) level in chloroform. 12

**Figure S2.** Optimized geometries of isomers of **2** at B3LYP/6-311G(d) level in chloroform. 14

**Table S9.** Experimental and calculated ^13^C NMR chemical shifts of **3**. 15

**Table S10.** Experimental and calculated ^1^H NMR chemical shifts of **3**. 15

**Table S11.** The results of the DP4+ analysis of **3** 16

**Table S12.** Conformational analysis of the optimized isomers of **3** at B3LYP/6-311G(d) level in chloroform. 16

**Figure S3.** Optimized geometries of isomers of **3** at B3LYP/6-311G(d) level in chloroform. 17

**Table S13.** Optimized cartesian coordinates of conformers of (1*S,*2*R,*8*R,*9*S,*10*R*)-**1** at B3LYP/6-311G(d) level in methanol. 18

**Table S14.** Optimized cartesian coordinates of conformers of (1*S,*2*S,*8*R,*9*S,*10*R*)-**1** at B3LYP/6-311G(d) level in methanol. 19

**Table S15.** Optimized cartesian coordinates of conformers of (1*R,*2*S,*8*R,*9*S,*10*R*)-**1** at B3LYP/6-311G(d) level in methanol. 20

**Table S16.** Optimized cartesian coordinates of conformers of (1*R,*2*R,*8*R,*9*S,*10*R*)-**1** at B3LYP/6-311G(d) level in methanol. 21

**Table S17.** Optimized cartesian coordinates of conformers of (1*S,*2*R,*8*R,*9*S,*10*S)*-1 at B3LYP/6-311G(d) level in methanol. 22

**Table S18.** Optimized cartesian coordinates of conformers of (1*S,*2*S,*8*R,*9*S,*10*S)*-**1** at B3LYP/6-311G(d) level in methanol. 23

**Table S19.** Optimized cartesian coordinates of conformers of (1*R,*2*S,*8*R,*9*S,*10*S)*-**1** at B3LYP/6-311G(d) level in methanol. 24

**Table S20.** Optimized cartesian coordinates of conformers of (1*R,*2*R,*8*R,*9*S,*10*S)*-**1** at B3LYP/6-311G(d) level in methanol. 26

**Table S21.** Optimized cartesian coordinates of conformers of (1*R**,*2*R,*4*R,*5*R,*10*R*)-**2** at B3LYP/6-311G(d) level in chloroform. 27

**Table S22.** Optimized cartesian coordinates of conformers of (1*R,*2*R,*4*R,*5*R,*10*S)*-**2** at B3LYP/6-311G(d) level in chloroform. 28

**Table S23.** Optimized cartesian coordinates of conformers of (1*R,2R,4R,*5*S,*10*R*)-**2** at B3LYP/6-311G(d) level in chloroform. 29

**Table S24.** Optimized cartesian coordinates of conformers of (1*R,*2*R,*4*R,*5*S,*10*S)*-**2** at B3LYP/6-311G(d) level in chloroform. 30

**Table S25.** Optimized cartesian coordinates of conformers of (1*S,2R,4R,*5*R,*10*R*)-**2** at B3LYP/6-311G(d) level in chloroform. 32

**Table S26.** Optimized cartesian coordinates of conformers of (1*S,*2*R,*4*R,*5*R,*10*R*)-**2** at B3LYP/6-311G(d) level in chloroform. 33

**Table S27.** Optimized cartesian coordinates of conformers of (1*S,*2*R,*4*R,*5*R,*10*S)*-**2** at B3LYP/6-311G(d) level in chloroform. 34

**Table S28.** Optimized cartesian coordinates of conformers of (1*S,*2*R,*4*R,*5*S,*10*R*)-**2** at B3LYP/6-311G(d) level in chloroform. 35

**Table S29.** Optimized cartesian coordinates of conformers of (1*S,*2*R,*4*R,*5*S,*10*R*)-**2** at B3LYP/6-311G(d) level in chloroform. 37

**Table S30.** Optimized cartesian coordinates of conformers of (1*S,*2*R,*4*R,*5*S,*10*S)*-**2** at B3LYP/6-311G(d) level in chloroform. 38

**Table S31.** Optimized cartesian coordinates of conformers of (1*R,*2*R,*4*S,*5*S,*10*R*)-**3** and (1*R,*2*R,*4*S,*5*S,*10*R*)-**3** at B3LYP/6-311G(d) level in chloroform. 39

**Table S32.** Optimized cartesian coordinates of conformers of (1*R,*2*R,*4*R,*5*R,*10*R*)- at B3LYP/6-311G(d) level in chloroform. 40

**Table S33.** Optimized cartesian coordinates of conformers of (1*R,*2*R,*4*R,*5*R,*10*S)*- at B3LYP/6-311G(d) level in chloroform. 42

**Figure S4.** ^1^H NMR (500 MHz) spectrum of compound **1** in MeOD 44

**Figure S5.** ^13^C NMR (125 MHz) spectrum of compound **1** in MeOD 44

**Figure S6.** HSQC spectrum of compound **1** in MeOD 45

**Figure S7.** ^1^H-^1^H COSYspectrum of compound **1** in MeOD 45

**Figure S8.** HMBC spectrum of compound **1** in MeOD 46

**Figure S9.** ROESY spectrum of compound **1** in MeOD 46

**Figure S10.** HRESIMS of compound **1** 47

**Figure S11.** ^1^H NMR (500 MHz) spectrum of compound **2** in CDCl_3_ 48

**Figure S12.** ^13^C NMR (125 MHz) Spectrum of compound **2** in CDCl_3_ 48

**Figure S13.** HSQC spectrum of compound **2** in CDCl_3_ 49

**Figure S14.** ^1^H-^1^H COSY spectrum of compound **2** in CDCl_3_ 49

**Figure S15.** HMBC spectrum of compound **2** in CDCl_3_ 50

**Figure S16.** ROESY spectrum of compound **2** in CDCl_3_ 50

**Figure S17.** HRESIMS of compound **2** 51

**Figure S18.** ^1^H NMR (500 MHz) spectrum of compound **3** in CDCl_3_ 52

**Figure S19.** ^13^C NMR (125 MHz) spectrum of compound **3** in CDCl_3_ 52

**Figure S20.** HSQC spectrum of compound **3** in CDCl_3_ 53

**Figure S21.** ^1^H-^1^H COSY spectrum of compound **3** in CDCl_3_ 53

**Figure S22.** HMBC spectrum of compound **3** in CDCl_3_ 54

**Figure S23.** ROESY spectrum of compound **3** in CDCl_3_ 54

**Figure S24.** HRESIMS of compound **3** 55

**Figure S25.** Dose-response-curves of compounds **2**, **3**, **5**, and **6** against glucagon-induced hepatic glucose production. 56

**Figure S26.** Phylogenetic tree of *Penicillium* sp.. 56

## Table S1. Experimental and calculated ^13^C NMR chemical shifts of 1.

| No. | Experimental  (*δ*_C_, ppm) | Calculated (*δ*_C_, ppm) | | | | | | | |
| --- | --- | --- | --- | --- | --- | --- | --- | --- | --- |
|  |  | 1 | 2 | 3 | 4 | 5 | 6 | 7 | 8 |
| 1 | 80.3 | 84.7 | 85.4 | 80.3 | 81.2 | 78.9 | 77.2 | 78.5 | 75.7 |
| 2 | 75.6 | 77.8 | 78.3 | 77.3 | 78.5 | 78.0 | 77.5 | 76.2 | 77.7 |
| 3 | 45.6 | 47.2 | 48.5 | 43.6 | 47.6 | 44.9 | 45.0 | 47.2 | 51.2 |
| 4 | 122.8 | 131.7 | 131.5 | 138.5 | 134.0 | 132.3 | 132.0 | 138.9 | 131.3 |
| 5 | 144.7 | 154.1 | 153.6 | 149.4 | 152.5 | 151.7 | 151.0 | 151.2 | 152.3 |
| 6 | 36.1 | 40.7 | 40.8 | 38.8 | 39.8 | 41.2 | 41.0 | 39.0 | 40.5 |
| 7 | 45.1 | 47.3 | 46.7 | 47.9 | 46.9 | 46.2 | 47.1 | 47.1 | 43.9 |
| 8 | 63.7 | 65.4 | 65.2 | 67.7 | 66.3 | 67.2 | 68.2 | 67.7 | 68.7 |
| 9 | 67.1 | 68.6 | 68.7 | 67.5 | 68.1 | 69.0 | 71.0 | 69.5 | 68.7 |
| 10 | 75.6 | 80.8 | 80.9 | 76.4 | 77.6 | 72.1 | 72.3 | 73.4 | 73.1 |
| 11 | 38.1 | 38.9 | 40.4 | 35.3 | 37.0 | 33.2 | 33.2 | 36.4 | 39.8 |
| 12 | 22.3 | 27.6 | 22.8 | 31.9 | 23.6 | 29.7 | 23.9 | 29.7 | 21.9 |
| 13 | 28.5 | 30.4 | 31.0 | 34.0 | 31.2 | 28.7 | 28.7 | 34.4 | 28.9 |
| 14 | 26.7 | 27.4 | 26.4 | 28.6 | 28.2 | 29.8 | 27.7 | 28.8 | 28.3 |
| 15 | 16.5 | 17.5 | 17.5 | 18.6 | 20.4 | 21.4 | 22.9 | 23.7 | 26.1 |
| **R^2^** |  | **0.9979** | **0.9987** | **0.9872** | **0.9967** | **0.9896** | **0.9905** | **0.9861** | **0.9889** |
| **MAE** |  | **3.4** | **3.3** | **3.8** | **3.1** | **3.8** | **3.6** | **4.3** | **3.8** |
| **CMAE** |  | **1.7** | **1.1** | **3.0** | **1.6** | **3.0** | **2.9** | **3.2** | **3.0** |

## Table S2. Experimental and calculated ^1^H NMR chemical shifts of 1.

| No. | Experimental  (*δ*_H_, ppm) | Calculated (*δ*_H_, ppm) | | | | | | | |
| --- | --- | --- | --- | --- | --- | --- | --- | --- | --- |
|  |  | 1 | 2 | 3 | 4 | 5 | 6 | 7 | 8 |
| 1 | 3.44 | 3.55 | 3.68 | 3.54 | 3.80 | 3.70 | 4.12 | 3.66 | 3.73 |
| 3a | 2.28 | 2.39 | 2.31 | 2.44 | 2.45 | 2.30 | 2.38 | 2.46 | 2.53 |
| 3b | 2.20 | 2.32 | 2.25 | 2.05 | 2.13 | 2.20 | 2.02 | 2.16 | 2.23 |
| 4 | 5.47 | 5.76 | 5.81 | 6.36 | 5.94 | 5.88 | 5.95 | 6.08 | 5.77 |
| 5 | 5.43 | 5.93 | 5.84 | 5.95 | 6.04 | 5.96 | 5.96 | 5.96 | 5.99 |
| 7a | 1.91 | 2.00 | 2.02 | 2.03 | 1.98 | 1.88 | 1.97 | 2.22 | 1.87 |
| 7b | 1.27 | 1.29 | 1.34 | 1.57 | 1.37 | 1.86 | 1.92 | 2.01 | 1.75 |
| 8 | 2.93 | 2.93 | 2.88 | 2.97 | 2.95 | 3.06 | 3.09 | 2.98 | 3.19 |
| 10 | 3.44 | 3.59 | 3.59 | 4.32 | 3.76 | 4.51 | 4.26 | 4.30 | 4.16 |
| 11a | 2.01 | 2.41 | 2.09 | 2.05 | 2.00 | 2.09 | 1.87 | 2.54 | 2.05 |
| 11b | 1.22 | 1.19 | 1.07 | 1.77 | 1.78 | 1.87 | 1.64 | 1.85 | 1.54 |
| 12 | 1.08 | 1.29 | 1.12 | 1.17 | 1.23 | 1.28 | 1.08 | 1.23 | 1.12 |
| 13 | 1.06 | 1.06 | 1.05 | 1.09 | 1.09 | 1.13 | 1.13 | 1.09 | 1.08 |
| 14 | 1.11 | 1.14 | 1.14 | 1.09 | 1.13 | 1.12 | 1.15 | 1.14 | 1.17 |
| 15 | 1.24 | 1.26 | 1.24 | 1.22 | 1.33 | 1.26 | 1.26 | 1.22 | 1.45 |
| **R^2^** |  | **0.9944** | **0.9978** | **0.9773** | **0.9892** | **0.9669** | **0.9728** | **0.9702** | **0.9863** |
| **MAE** |  | **0.14** | **0.12** | **0.26** | **0.20** | **0.27** | **0.29** | **0.33** | **0.24** |
| **CMAE** |  | **0.08** | **0.05** | **0.17** | **0.10** | **0.21** | **0.19** | **0.21** | **0.14** |

## Table S3. The results of the DP4+ analysis of 1.


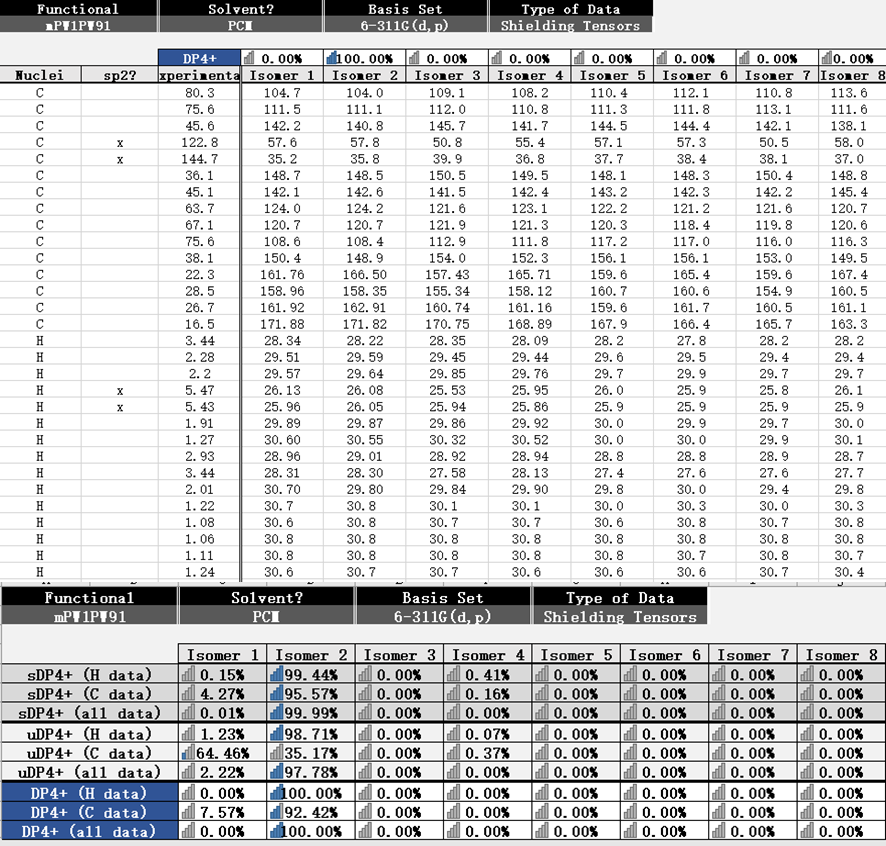


## Table S4. Conformational analysis of the optimized isomers of 1 at B3LYP/6-311G(d) level in methanol.

| Conformations | *G*  (hartree) | *ΔG*  (kcal/mol) | Boltzmann distributions (%) |
| --- | --- | --- | --- |
| (1*S,*2*R,*8*R,*9*S,*10*R*)-**1-1** | -888.034886 | 0 | 62.2 |
| (1*S,*2*R,*8*R,*9*S,*10*R*)-**1-2** | -888.033983 | 0.566641 | 23.9 |
| (1*S,*2*R,*8*R,*9*S,*10*R)*-**1-3** | -888.033468 | 0.889808 | 13.9 |
| (1*S,*2*S,*8*R,*9*S,*10*R*)-**1-1** | -888.036161 | 0 | 33.9 |
| (1*S,*2*S,*8*R,*9*S,*10*R*)-**1-2** | -888.035968 | 0.121109 | 27.6 |
| (1*S,*2*S,*8*R,*9*S,*10*R*)-**1-3** | -888.035417 | 0.466866 | 15.4 |
| (1*S,*2*S,*8*R,*9*S,*10*R*)-**1-4** | -888.035362 | 0.501380 | 14.5 |
| (1*S,*2*S,*8*R,*9*S,*10*R*)-**1**-**5** | -888.034846 | 0.825174 | 8.4 |
| (1*R,*2*S,*8*R,*9*S,*10*R*)-**1-1** | -888.039076 | 0 | 94.8 |
| (1*R,*2*S,*8*R,*9*S,*10*R*)-**1-2** | -888.035992 | 1.93523899 | 3.6 |
| (1*R,*2*S,*8*R,*9*S,*10*R*)-**1-3** | -888.035237 | 2.409008587 | 1.6 |
| (1*R,*2*R,*8*R,*9*S,*10*R*)-**1-1** | -888.035403 | 0 | 47.5 |
| (1*R,*2*R,*8*R,*9*S,*10*R*)-**1-2** | -888.034783 | 0.389055828 | 24.6 |
| (1*R,*2*R,*8*R,*9*S,*10*R*)-**1-3** | -888.033987 | 0.88855331 | 10.6 |
| (1*R,*2*R,*8*R,*9*S,*10*R*)-**1-4** | -888.033625 | 1.115711713 | 7.2 |
| (1*R,*2*R,*8*R,*9*S,*10*R)*-**1-5** | -888.033316 | 1.309612118 | 5.2 |
| (1*R,*2*R,*8*R,*9*S,*10*R*)-**1-6** | -888.033053 | 1.47464709 | 3.9 |
| (1*S,*2*R,*8*R,*9*S,*10*S*)-**1-1** | -888.032733 | 0 | 53.0 |
| (1*S,*2*R,*8*R,*9*S,*10*S*)-**1-2** | -888.032445 | 0.180722707 | 39.1 |
| (1*S,*2*R,*8*R,*9*S,*10*S*)-**1-3** | -888.030562 | 1.362322907 | 5.3 |
| (1*S,*2*R,*8*R,*9*S,*10*S*)-**1-4** | -888.0299 | 1.77773413 | 2.6 |
| (1*S,*2*S,*8*R,*9*S,*10*S*)-**1-1** | -888.03582 | 0 | 98.8 |
| (1*S,*2*S,*8*R,*9*S,*10*S*)-**1-2** | -888.031626 | 2.631774424 | 1.2 |
| (1*R,*2*S,*8*R,*9*S,*10*S*)-**1-1** | -888.034641 | 0 | 94.0 |
| (1*R,*2*S,*8*R,*9*S,*10*S*)-**1-2** | -888.031795 | 1.785891752 | 4.6 |
| (1*R,*2*S,*8*R,*9*S,*10*S*)-**1-3** | -888.030695 | 2.476152092 | 1.4 |
| (1*R,*2*R,*8*R,*9*S,*10*S*)-**1-1** | -888.034934 | 0 | 36.5 |
| (1*R,*2*R,*8*R,*9*S,*10*S*)-**1-2** | -888.034514 | 0.263553948 | 23.4 |
| (1*R,*2*R,*8*R,*9*S,*10*S*)-**1-3** | -888.034275 | 0.413528695 | 18.1 |
| (1*R,*2*R,*8*R,*9*S,*10*S*)-**1-4** | -888.033938 | 0.624999362 | 12.7 |
| (1*R,*2*R,*8*R,*9*S,*10*S*)-**1-5** | -888.033647 | 0.807604598 | 9.3 |


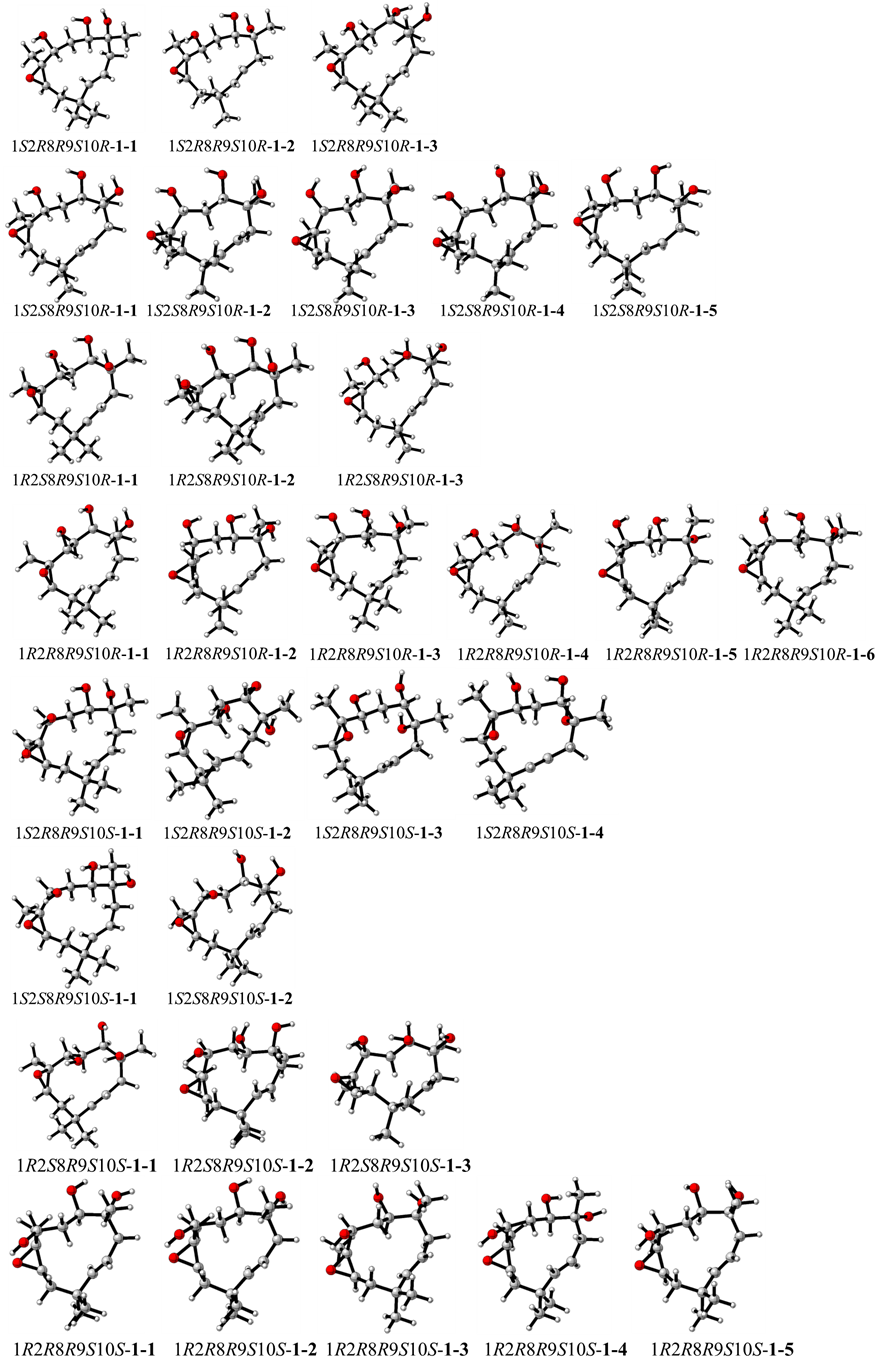


## Figure S1. Optimized geometries of isomers of 1 at B3LYP/6-311G(d) level in methanol.

## Table S5. Experimental and calculated ^13^C NMR chemical shifts of 2.

| No. | Experimental  (*δ*_C_, ppm) | Calculated (*δ*_C_, ppm) | | | | | | | |
| --- | --- | --- | --- | --- | --- | --- | --- | --- | --- |
|  |  | 1 | 2 | 3 | 4 | 5 | 6 | 7 | 8 |
| 1 | 78.9 | 81.0 | 84.2 | 80.0 | 83.4 | 76.2 | 75.5 | 74.9 | 74.0 |
| 2 | 25.8 | 31.8 | 32.2 | 30.2 | 30.0 | 30.7 | 30.5 | 28.4 | 27.5 |
| 3 | 14.6 | 21.8 | 20.8 | 16.4 | 16.6 | 20.1 | 20.1 | 14.5 | 13.9 |
| 4 | 24.5 | 26.7 | 27.0 | 30.7 | 26.7 | 22.5 | 22.8 | 25.6 | 21.8 |
| 5 | 72.7 | 76.4 | 76.4 | 76.4 | 76.4 | 76.4 | 76.4 | 76.4 | 76.4 |
| 6 | 39.4 | 45.1 | 46.1 | 44.0 | 44.3 | 45.4 | 47.7 | 44.4 | 45.4 |
| 7 | 38.9 | 41.9 | 41.9 | 41.9 | 41.9 | 41.9 | 41.9 | 41.9 | 41.9 |
| 8 | 126.8 | 141.4 | 137.9 | 139.7 | 136.8 | 140.7 | 139.7 | 138.4 | 137.5 |
| 9 | 132.2 | 143.4 | 144.1 | 142.5 | 141.8 | 144.9 | 143.3 | 143.8 | 140.9 |
| 10 | 68.6 | 71.6 | 72.2 | 71.1 | 72.0 | 71.7 | 72.1 | 72.1 | 72.5 |
| 11 | 36.4 | 38.9 | 38.8 | 39.1 | 38.4 | 39.1 | 39.0 | 39.2 | 39.4 |
| 12 | 12.6 | 15.9 | 16.4 | 14.3 | 14.2 | 22.2 | 22.9 | 19.1 | 19.4 |
| 13 | 25.9 | 31.4 | 26.9 | 30.9 | 26.5 | 31.6 | 26.8 | 30.9 | 26.3 |
| 14 | 21.9 | 24.4 | 29.6 | 18.2 | 22.1 | 24.6 | 29.2 | 18.1 | 22.2 |
| 15 | 18.4 | 21.6 | 21.7 | 21.3 | 21.7 | 22.2 | 21.5 | 22.4 | 21.5 |
| 16 | 21.5 | 23.7 | 23.4 | 23.6 | 23.5 | 23.7 | 23.9 | 23.6 | 23.9 |
| 17 | 170.3 | 178.3 | 176.1 | 176.4 | 176.0 | 178.3 | 180.4 | 177.1 | 180.4 |
| **R^2^** |  | **0.9971** | **0.9974** | **0.9966** | **0.9989** | **0.994** | **0.9935** | **0.9949** | **0.9955** |
| **MAE** |  | **5.1** | **5.1** | **4.4** | **3.7** | **5.4** | **5.6** | **4.5** | **4.3** |
| **CMAE** |  | **2.0** | **2.1** | **2.0** | **1.2** | **2.8** | **2.9** | **2.4** | **2.1** |

## Table S6. Experimental and calculated ^1^H NMR chemical shifts of 2.

| No. | Experimental  (*δ*_H_, ppm) | Calculated (*δ*_H_, ppm) | | | | | | | |
| --- | --- | --- | --- | --- | --- | --- | --- | --- | --- |
|  |  | 1 | 2 | 3 | 4 | 5 | 6 | 7 | 8 |
| 1 | 3.04 | 2.59 | 3.00 | 2.70 | 2.99 | 3.78 | 3.99 | 3.93 | 4.05 |
| 3a | 0.53 | 0.48 | 0.63 | 0.30 | 0.30 | 0.73 | 1.04 | 0.47 | 0.50 |
| 3b | 0.38 | 0.12 | 0.25 | 0.23 | 0.29 | 0.03 | 0.05 | 0.20 | 0.10 |
| 4 | 1.01 | 0.91 | 0.72 | 0.94 | 0.77 | 1.34 | 1.46 | 1.38 | 1.36 |
| 5 | 2.93 | 3.65 | 3.52 | 3.15 | 2.85 | 3.73 | 3.49 | 3.20 | 2.86 |
| 7a | 2.58 | 1.44 | 2.94 | 3.04 | 2.75 | 3.11 | 2.75 | 2.45 | 2.48 |
| 7b | 1.76 | 1.44 | 1.68 | 1.72 | 1.72 | 1.48 | 1.67 | 1.82 | 1.72 |
| 8 | 5.46 | 6.06 | 6.14 | 5.94 | 5.94 | 6.03 | 6.25 | 5.95 | 6.07 |
| 10 | 5.61 | 6.19 | 5.92 | 6.00 | 5.73 | 6.46 | 5.99 | 6.15 | 5.73 |
| 11a | 2.25 | 2.35 | 2.40 | 2.37 | 2.35 | 2.42 | 2.63 | 2.19 | 2.50 |
| 11b | 1.83 | 1.80 | 1.83 | 1.85 | 1.85 | 1.97 | 1.78 | 2.05 | 1.73 |
| 12 | 0.87 | 1.36 | 1.16 | 1.14 | 0.85 | 1.38 | 1.14 | 1.27 | 0.79 |
| 13 | 1.15 | 0.96 | 1.11 | 1.06 | 1.13 | 0.99 | 1.13 | 1.06 | 1.15 |
| 14 | 0.98 | 0.95 | 0.92 | 0.88 | 0.88 | 1.00 | 0.94 | 0.92 | 0.89 |
| 15 | 1.81 | 1.84 | 1.96 | 1.79 | 1.91 | 1.93 | 1.97 | 1.96 | 1.93 |
| 16 | 2.01 | 2.05 | 1.98 | 1.98 | 1.96 | 2.04 | 2.07 | 1.99 | 2.06 |
| **R^2^** |  | **0.9422** | **0.9868** | **0.9879** | **0.9953** | **0.9765** | **0.9733** | **0.98** | **0.9735** |
| **MAE** |  | **0.32** | **0.21** | **0.19** | **0.12** | **0.36** | **0.32** | **0.25** | **0.21** |
| **CMAE** |  | **0.24** | **0.15** | **0.11** | **0.09** | **0.20** | **0.22** | **0.16** | **0.18** |

## Table S7. The results of the DP4+ analysis of 2


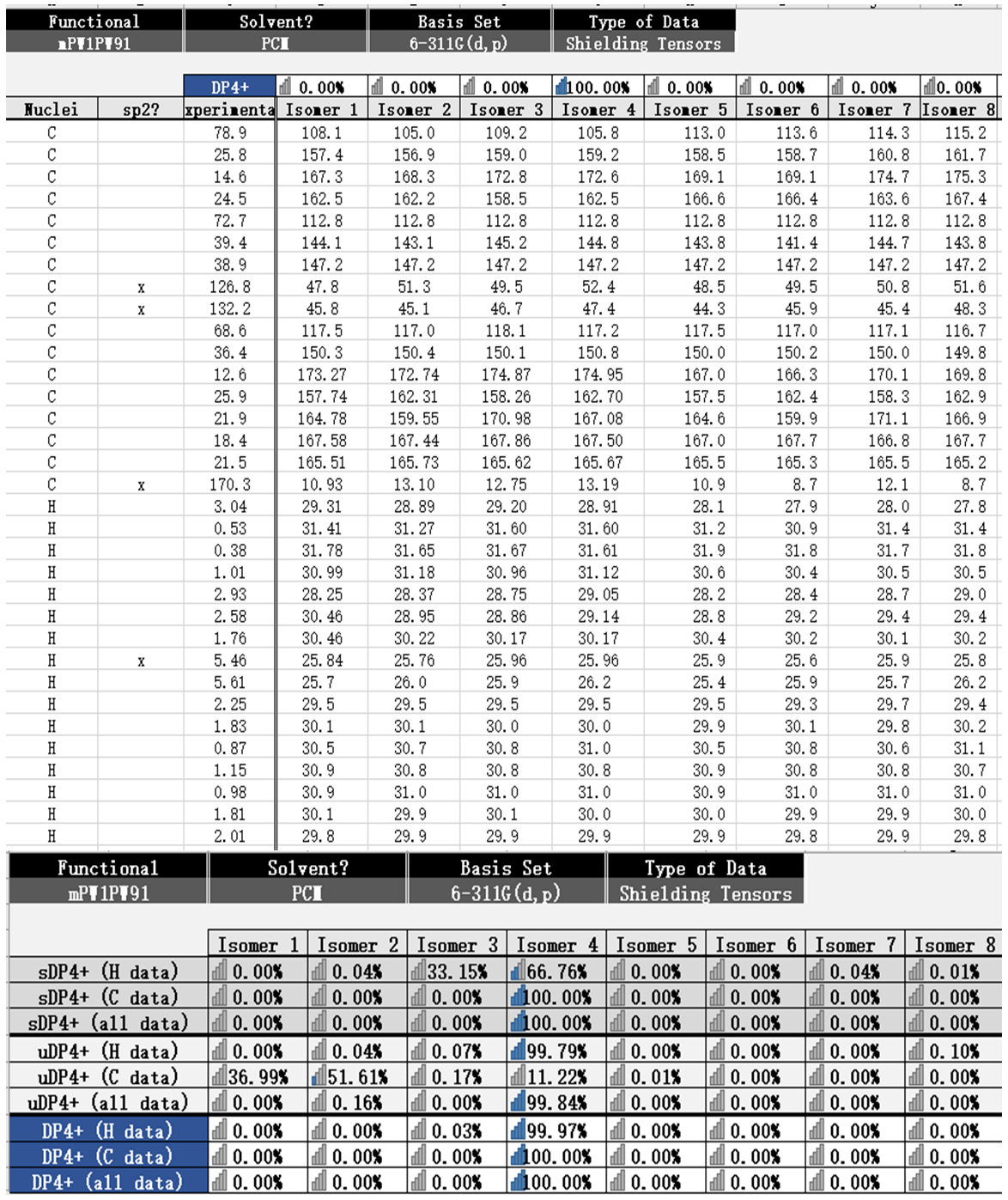


## Table S8. Conformational analysis of the optimized isomers of 2 at B3LYP/6-311G(d) level in chloroform.

| Conformations | *G*  (hartree) | *ΔG*  (kcal/mol) | Boltzmann distributions (%) |
| --- | --- | --- | --- |
| (1*R*,2*R,*4*R,*5*R*,10*R*)-**2-1** | -965.47262 | 0 | 58.8 |
| (1*R*,2*R,*4*R,*5*R*,10*R*)-**2-2** | -965.47182 | 0.50200752 | 25.2 |
| (1*R*,2*R,*4*R,*5*R*,10*R*)-**2-3** | -965.470351 | 1.4219363 | 5.3 |
| (1*R*,2*R,*4*R,*5*R*,10*R*)-**2-4** | -965.470221 | 1.503512522 | 4.6 |
| (1*R*,2*R,*4*R,*5*R*,10*R*)-**2-5** | -965.470006 | 1.638427043 | 3.7 |
| (1*R*,2*R,*4*R,*5*R*,10*R*)-**2-6** | -965.469619 | 1.881273181 | 2.4 |
| (1*R*,2*R,*4*R,*5*R*,10*S*)-**2-1** | -965.471808 | 0 | 75.1 |
| (1*R*,2*R,*4*R,*5*R*,10*S*)-**2-2** | -965.46992 | 1.184737747 | 10.1 |
| (1*R*,2*R,*4*R,*5*R*,10*S*)-**2-3** | -965.469637 | 1.362322907 | 7.5 |
| (1*R*,2*R,*4*R,*5*R*,10*S*)-**2-4** | -965.469609 | 1.37989321 | 7.3 |
| (1*R*,2*R,*4*R,*5*S,*10*R*)-**2-1** | -965.477681 | 0 | 72.6 |
| (1*R*,2*R,*4*R,*5*S,*10*R*)-**2-2** | -965.476084 | 1.002132512 | 13.3 |
| (1*R*,2*R,*4*R,*5*S,*10*R*)-**2-3** | -965.475653 | 1.272589063 | 8.4 |
| (1*R*,2*R,*4*R,*5*S,*10*R*)-**2-4** | -965.475071 | 1.637799534 | 4.6 |
| (1*R*,2*R,*4*R,*5*S,*10*R*)-**2-5** | -965.473728 | 2.480544658 | 1.1 |
| (1*R*,2*R,*4*R,*5*S,*10*S)*-**2-1** | -965.476604 | 0 | 72.1 |
| (1*R*,2*R,*4*R,*5*S,*10*S)*-**2-2** | -965.475132 | 0.923693837 | 15.1 |
| (1*R*,2*R,*4*R,*5*S,*10*S)*-**2-3** | -965.474831 | 1.112574166 | 11.0 |
| (1*R*,2*R,*4*R,*5*S,*10*S)*-**2-4** | -965.473118 | 2.187497768 | 1.8 |
| (1*S,*2*R,*4*R,*5*R*,10*R*)-**2-1** | -965.470362 | 0 | 29.3 |
| (1*S,*2*R,*4*R,*5*R*,10*R*)-**2-2** | -965.470058 | 0.190762858 | 21.2 |
| (1*S,*2*R,*4*R,*5*R*,10*R*)-**2-3** | -965.469825 | 0.336972548 | 16.6 |
| (1*S,*2*R,*4*R,*5*R*,10*R*)-**2-4** | -965.469666 | 0.436746542 | 14.0 |
| (1*S,*2*R,*4*R,*5*R*,10*R*)-**2-5** | -965.46953 | 0.522087821 | 12.1 |
| (1*S,*2*R,*4*R,*5*R*,10*R*)-**2-6** | -965.467931 | 1.525475351 | 2.2 |
| (1*S,*2*R,*4*R,*5*R*,10*R*)-**2-7** | -965.46773 | 1.651604741 | 1.8 |
| (1*S,*2*R,*4*R,*5*R*,10*R*)-**2-8** | -965.467515 | 1.786519262 | 1.4 |
| (1*S,*2*R,*4*R,*5*R*,10*R*)-**2-9** | -965.467455 | 1.824169826 | 1.3 |
| (1*S,*2*R,*4*R,*5*R*,10*S*)-**2-1** | -965.473965 | 0 | 88.7 |
| (1*S,*2*R,*4*R,*5*R*,10*S*)-**2-2** | -965.47133 | 1.653487269 | 5.4 |
| (1*S,*2*R,*4*R,*5*R*,10*S*)-**2-3** | -965.471228 | 1.72493228 | 4.9 |
| (1*S,*2*R,*4*R,*5*R*,10*S*)-**2-4** | -965.469789 | 2.620479254 | 1.0 |
| (1*S,*2*R,*4*R,*5*S,*10*R*)-**2-1** | -965.474801 | 0 | 30.6 |
| (1*S,*2*R,*4*R,*5*S,*10*R*)-**2-2** | -965.474769 | 0.020080301 | 29.6 |
| (1*S,*2*R,*4*R,*5*S,*10*R*)-**2-3** | -965.474463 | 0.21209827 | 21.4 |
| (1*S,*2*R,*4*R,*5*S,*10*R*)-**2-4** | -965.473712 | 0.683357737 | 9.7 |
| (1*S,*2*R,*4*R,*5*S,*10*R*)-**2-5** | -965.472864 | 1.215485708 | 3.9 |
| (1*S,*2*R,*4*R,*5*S,*10*R*)-**2-6** | -965.472739 | 1.293924383 | 3.4 |
| (1*S,*2*R,*4*R,*5*S,*10*R*)-**2-7** | -965.471843 | 1.85622805 | 1.4 |
| (1*S,*2*R,*4*R,*5*S,*10*S*)-**2-1** | -965.478473 | 0 | 81.0 |
| (1*S,*2*R,*4*R,*5*S,*10*S*)-**2-2** | -965.47633 | 1.344752644 | 8.3 |
| (1*S,*2*R,*4*R,*5*S,*10*S*)-**2-3** | -965.476049 | 1.521082786 | 6.2 |
| (1*S,*2*R,*4*R,*5*S,*10*S*)-**2-4** | -965.475741 | 1.714355681 | 4.5 |


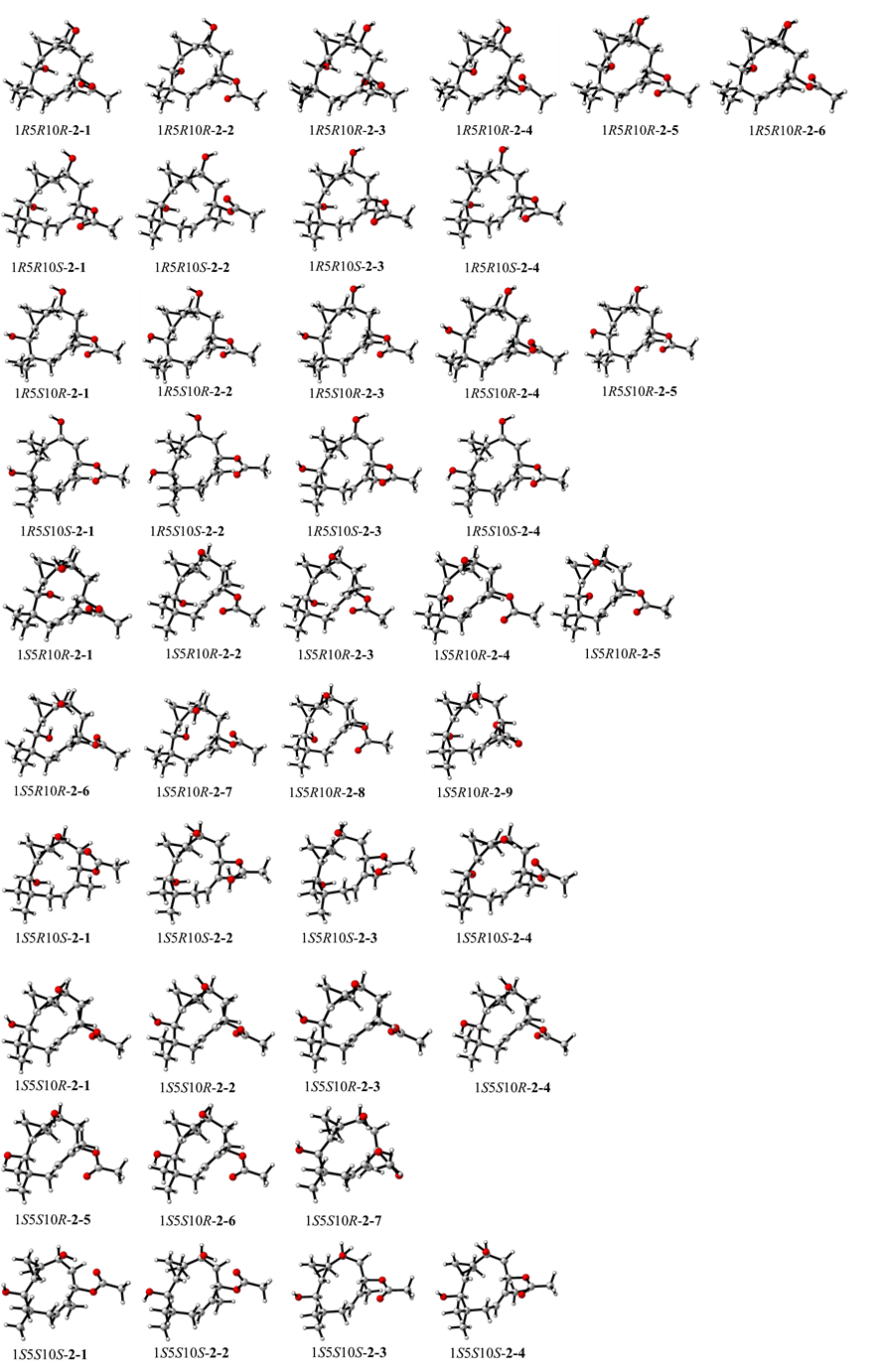


## Figure S2. Optimized geometries of isomers of 2 at B3LYP/6-311G(d) level in chloroform.

## Table S9. Experimental and calculated ^13^C NMR chemical shifts of 3.

| No. | Experimental  (*δ*_C_, ppm) | Calculated (*δ*_C_, ppm) | | | |
| --- | --- | --- | --- | --- | --- |
|  |  | 1 | 2 | 3 | 4 |
| 1 | 59.7 | 62.0 | 62.0 | 60.5 | 59.6 |
| 2 | 59.6 | 62.7 | 62.6 | 62.0 | 62.6 |
| 3 | 42.9 | 46.0 | 46.3 | 43.2 | 44.1 |
| 4 | 52.6 | 55.5 | 55.6 | 55.9 | 53.4 |
| 5 | 65.9 | 67.1 | 68.0 | 66.2 | 65.3 |
| 6 | 34.4 | 39.8 | 39.4 | 39.3 | 39.0 |
| 7 | 38.7 | 44.2 | 42.1 | 42.5 | 44.2 |
| 8 | 127.7 | 138.2 | 136.3 | 137.9 | 135.7 |
| 9 | 132.6 | 143.1 | 142.3 | 141.7 | 141.9 |
| 10 | 68.7 | 72.5 | 72.5 | 72.8 | 72.7 |
| 11 | 31.1 | 34.7 | 34.7 | 33.1 | 35.2 |
| 12 | 18.2 | 19.4 | 20.8 | 20.4 | 22.9 |
| 13 | 17.9 | 24.1 | 19.2 | 30.5 | 27.1 |
| 14 | 29.7 | 26.4 | 30.0 | 21.0 | 23.8 |
| 15 | 18.7 | 24.3 | 23.6 | 23.4 | 23.5 |
| 16 | 21.3 | 22.1 | 23.3 | 22.5 | 22.8 |
| 17 | 170.1 | 178.2 | 176.3 | 176.9 | 177.0 |
| **R^2^** |  | **0.9968** | **0.9987** | **0.9913** | **0.994** |
| **MAE** |  | **4.5** | **3.8** | **4.5** | **4.4** |
| **CMAE** |  | **2.0** | **1.3** | **2.8** | **2.7** |

## Table S10. Experimental and calculated ^1^H NMR chemical shifts of 3.

| No. | Experimental  (*δ*_H_, ppm) | Calculated (*δ*_H_, ppm) | | | |
| --- | --- | --- | --- | --- | --- |
|  |  | 1 | 2 | 3 | 4 |
| 1 | 2.71 | 2.40 | 2.63 | 2.70 | 2.99 |
| 3a | 2.66 | 2.66 | 2.53 | 0.30 | 0.30 |
| 3b | 0.63 | 0.54 | 0.50 | 0.23 | 0.29 |
| 4 | 2.76 | 2.92 | 2.71 | 0.94 | 0.77 |
| 5 | 2.33 | 2.40 | 2.28 | 3.15 | 2.85 |
| 7a | 1.92 | 2.05 | 2.00 | 3.04 | 2.75 |
| 7b | 2.62 | 2.76 | 2.47 | 1.72 | 1.72 |
| 8 | 5.44 | 6.19 | 5.94 | 5.94 | 5.94 |
| 10 | 5.86 | 5.79 | 5.97 | 6.00 | 5.73 |
| 11a | 1.78 | 1.55 | 1.67 | 2.37 | 2.35 |
| 11b | 2.26 | 2.55 | 2.21 | 1.85 | 1.85 |
| 12 | 1.27 | 1.49 | 1.22 | 1.14 | 0.85 |
| 13 | 0.77 | 0.80 | 0.75 | 1.06 | 1.13 |
| 14 | 1.12 | 1.21 | 1.06 | 0.88 | 0.88 |
| 15 | 1.71 | 2.02 | 1.89 | 1.79 | 1.91 |
| 16 | 2.05 | 2.09 | 2.05 | 1.98 | 1.96 |
| **R^2^** |  | **0.9776** | **0.9931** | **0.9879** | **0.9953** |
| **MAE** |  | **0.18** | **0.11** | **0.24** | **0.22** |
| **CMAE** |  | **0.16** | **0.09** | **0.26** | **0.20** |

## Table S11. The results of the DP4+ analysis of 3

**
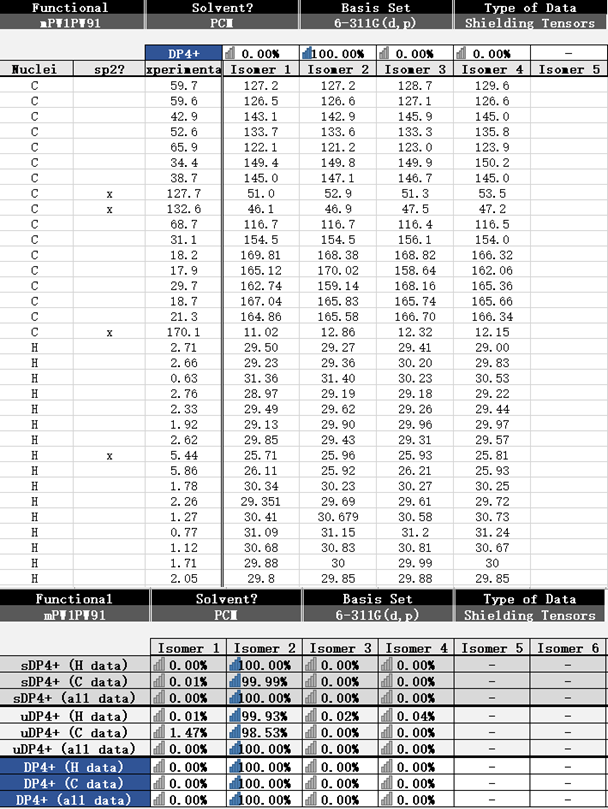
**

## Table S12. Conformational analysis of the optimized isomers of 3 at B3LYP/6-311G(d) level in chloroform.

| Conformations | *G*  (hartree) | *ΔG*  (kcal/mol) | Boltzmann di*S,*tribution*S,* (%) |
| --- | --- | --- | --- |
| (1*R*,2*R*,4*S,*5*S,*10*R*)**-3-1** | -964.033891 | 0 | 55.9 |
| (1*R*,2*R*,4*S,*5*S,*10*R*)**-3-2** | -964.033664 | 0.142445 | 44.1 |
| (1*R*,2*R*,4*S,*5*S,*10*S*)**-3-1** | -964.253093 | 0 | 91.0 |
| (1*R*,2*R*,4*S,*5*S,*10*S*)**-3-2** | -964.250906 | 1.372363 | 9.0 |
| (1*R*,2*R*,4*R*,5*R*,10*R*)**-3-1** | -964.259575 | 0 | 56.7 |
| (1*R*,2*R*,4*R*,5*R*,10*R*)**-3-2** | -964.258459 | 0.7003005 | 17.4 |
| (1*R*,2*R*,4*R*,5*R*,10*R*)**-3-3** | -964.258374 | 0.7536388 | 15.8 |
| (1*R*,2*R*,4*R*,5*R*1,0*R*)**-3-4** | -964.257507 | 1.2976894 | 6.3 |
| (1*R*,2*R*,4*R*,5*R*,10*R*)**-3-5** | -964.256709 | 1.7984419 | 2.7 |
| (1*R*,2*R*,4*R*,5*R*,10*R*)**-3-6** | -964.255815 | 2.3594353 | 1.1 |
| (1*R*,2*R*,4*R*,5*R*,10*S*)**-3-1** | -964.25892 | 0 | 98.1 |
| (1*R*,2*R*,4*R*,5*R*,10*S*)**-3-2** | -964.255176 | 2.349395 | 1.9 |

**
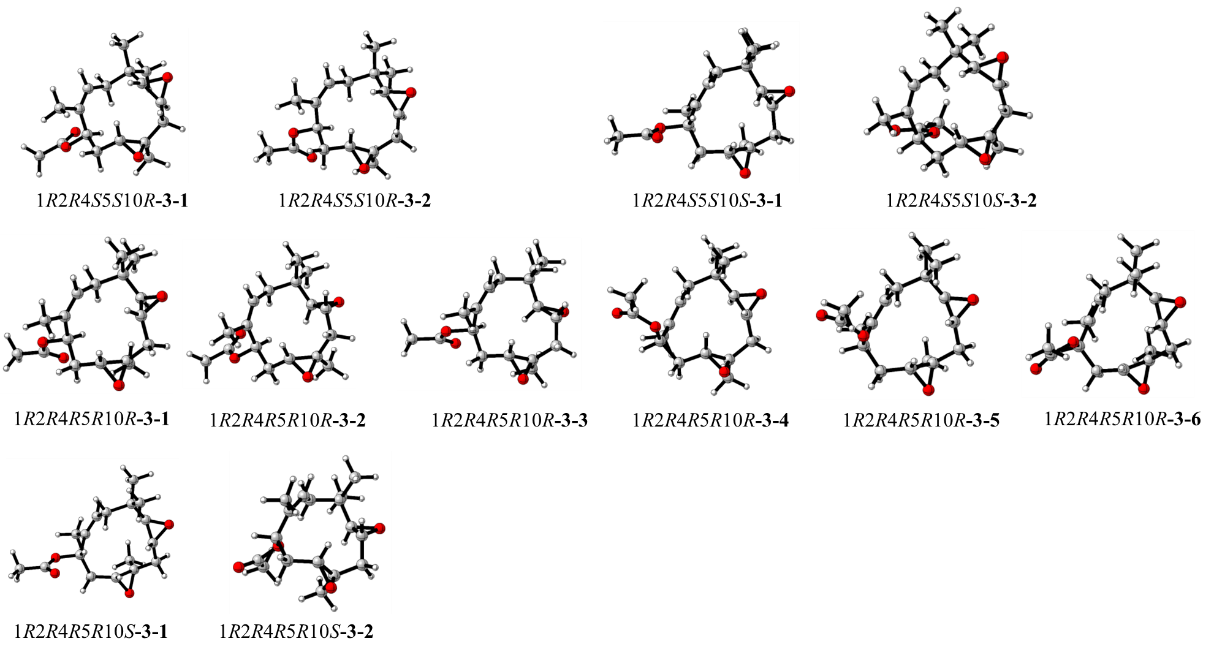
**

## Figure S3. Optimized geometries of isomers of 3 at B3LYP/6-311G(d) level in chloroform.

## Table S13. Optimized cartesian coordinates of conformers of (1*S,*2*R,*8*R,*9*S,*10*R*)-1 at B3LYP/6-311G(d) level in methanol.

|  | (1*S,*2*R,*8*R,*9*S,*10*R*)-**1**-1 | | | (1*S,*2*R,*8*R,*9*S,*10*R*)-**1**-2 | | | (1*S,*2*R,*8*R,*9*S,*10*R*)-**1**-3 | | |
| --- | --- | --- | --- | --- | --- | --- | --- | --- | --- |
| C | 1.50637 | 1.83069 | 0.28584 | 1.50428 | 1.79651 | 0.29024 | 1.26668 | 1.87522 | 0.05837 |
| C | 0.17912 | 1.77219 | -0.44454 | 0.21024 | 1.60068 | -0.4686 | -0.12064 | 1.47516 | -0.42454 |
| C | -0.92052 | 1.13883 | 0.40205 | -0.88467 | 1.01256 | 0.40097 | -1.0655 | 1.18616 | 0.72893 |
| C | -1.9172 | 0.37444 | -0.46792 | -2.05215 | 0.48826 | -0.44455 | -2.44494 | 0.61888 | 0.40242 |
| C | -2.69274 | -0.73739 | 0.25977 | -2.7268 | -0.76577 | 0.14702 | -2.53932 | -0.77913 | -0.28276 |
| C | -1.73957 | -1.69534 | 1.01678 | -1.86576 | -2.02238 | -0.127 | -1.65426 | -1.84365 | 0.4007 |
| C | -0.49314 | -2.05033 | 0.24882 | -0.43237 | -1.9717 | 0.32599 | -0.22874 | -1.88335 | -0.06951 |
| C | 0.72901 | -1.66206 | 0.61196 | 0.60423 | -1.83309 | -0.49783 | 0.84213 | -1.60128 | 0.66781 |
| C | 2.02809 | -1.81478 | -0.14834 | 2.07588 | -1.73808 | -0.15053 | 2.2779 | -1.5772 | 0.15951 |
| C | 2.38811 | -0.45209 | -0.81467 | 2.66336 | -0.41304 | -0.73042 | 2.47803 | -0.36537 | -0.79186 |
| C | 2.50292 | 0.75575 | 0.09107 | 2.60298 | 0.82319 | 0.14376 | 2.43842 | 1.00308 | -0.15375 |
| O | 2.64993 | 2.03014 | -0.58102 | 2.64542 | 2.09249 | -0.55332 | 2.17913 | 2.11259 | -1.04701 |
| O | -0.29146 | 3.07301 | -0.83771 | -0.30217 | 2.84256 | -0.98525 | -0.68668 | 2.53948 | -1.19991 |
| O | -2.89325 | 1.28164 | -1.00863 | -3.03012 | 1.52784 | -0.59737 | -3.10524 | 0.55066 | 1.66941 |
| O | -3.532 | -0.13686 | 1.25678 | -2.84499 | -0.65544 | 1.57646 | -3.89802 | -1.24106 | -0.03655 |
| C | 3.13435 | -2.23892 | 0.83595 | 2.33765 | -1.84249 | 1.35638 | 3.23791 | -1.4927 | 1.35723 |
| C | 1.92916 | -2.85942 | -1.26757 | 2.7853 | -2.90785 | -0.86597 | 2.59706 | -2.85381 | -0.64007 |
| C | -3.5553 | -1.50839 | -0.74435 | -4.11492 | -0.97685 | -0.462 | -2.34361 | -0.70955 | -1.79415 |
| C | 1.53099 | 2.65399 | 1.55008 | 1.42628 | 2.64149 | 1.53877 | 1.33115 | 2.9353 | 1.13164 |
| H | 3.21426 | 0.64087 | 0.90756 | 3.29308 | 0.79695 | 0.98553 | 3.27655 | 1.20523 | 0.51128 |
| H | 0.32914 | 1.18183 | -1.35062 | 0.40767 | 0.93073 | -1.30924 | -0.00719 | 0.59732 | -1.0576 |
| H | -0.47346 | 0.48447 | 1.14023 | -0.46965 | 0.23758 | 1.03402 | -0.57683 | 0.5191 | 1.43794 |
| H | -1.44585 | 1.91933 | 0.95756 | -1.26921 | 1.78738 | 1.06918 | -1.24692 | 2.12272 | 1.26269 |
| H | -1.37529 | -0.0962 | -1.29614 | -1.68552 | 0.21025 | -1.44153 | -2.98205 | 1.32495 | -0.24698 |
| H | -1.47286 | -1.22287 | 1.96436 | -2.37599 | -2.85722 | 0.3655 | -1.70438 | -1.69298 | 1.48208 |
| H | -2.30863 | -2.59696 | 1.26811 | -1.90237 | -2.21742 | -1.20224 | -2.1296 | -2.80751 | 0.19136 |
| H | -0.6338 | -2.60099 | -0.67805 | -0.2704 | -2.01718 | 1.39961 | -0.0901 | -2.16052 | -1.11257 |
| H | 0.83085 | -1.11376 | 1.54856 | 0.40314 | -1.78165 | -1.56946 | 0.69937 | -1.32032 | 1.71023 |
| H | 3.36027 | -0.56693 | -1.30974 | 3.72725 | -0.57568 | -0.9377 | 3.46188 | -0.46014 | -1.26741 |
| H | 1.66595 | -0.25387 | -1.60757 | 2.20297 | -0.20199 | -1.69811 | 1.74413 | -0.41928 | -1.598 |
| H | 0.31339 | 3.42176 | -1.50424 | 0.30589 | 3.15941 | -1.66408 | -0.06936 | 2.71495 | -1.92144 |
| H | -2.43211 | 2.1069 | -1.21187 | -2.53212 | 2.31041 | -0.87264 | -3.85006 | -0.05467 | 1.5327 |
| H | -3.95585 | 0.61523 | 0.81903 | -3.33271 | 0.15637 | 1.76436 | -4.49136 | -0.7662 | -0.63303 |
| H | 3.2193 | -1.54741 | 1.6778 | 3.4074 | -1.75979 | 1.56577 | 3.16112 | -2.39349 | 1.97226 |
| H | 4.10552 | -2.28128 | 0.3349 | 1.9977 | -2.80598 | 1.74361 | 3.01186 | -0.63726 | 1.99883 |
| H | 2.92156 | -3.22973 | 1.24573 | 1.82594 | -1.058 | 1.91865 | 4.2753 | -1.40094 | 1.02394 |
| H | 2.89147 | -2.96415 | -1.77532 | 2.37616 | -3.86647 | -0.53736 | 2.41981 | -3.74415 | -0.0321 |
| H | 1.65053 | -3.83687 | -0.86618 | 2.65834 | -2.84132 | -1.95043 | 1.9816 | -2.9326 | -1.53913 |
| H | 1.18862 | -2.57872 | -2.02046 | 3.85784 | -2.90066 | -0.65156 | 3.64476 | -2.85734 | -0.95405 |
| H | -2.93961 | -2.04937 | -1.46865 | -4.06625 | -1.04962 | -1.55161 | -2.49933 | -1.69692 | -2.23466 |
| H | -4.17854 | -2.23056 | -0.2131 | -4.55209 | -1.89763 | -0.06962 | -3.06887 | -0.01983 | -2.23555 |
| H | -4.20375 | -0.82565 | -1.2956 | -4.77157 | -0.14277 | -0.21336 | -1.3534 | -0.36178 | -2.08148 |
| H | 2.54179 | 2.69911 | 1.95759 | 0.95564 | 3.60108 | 1.31378 | 0.98039 | 2.55094 | 2.0919 |
| H | 1.19221 | 3.67082 | 1.34093 | 0.83641 | 2.14742 | 2.31419 | 2.35799 | 3.28191 | 1.25537 |
| H | 0.87114 | 2.22867 | 2.31021 | 2.42613 | 2.82751 | 1.93316 | 0.70761 | 3.78892 | 0.85614 |

## Table S14. Optimized cartesian coordinates of conformers of (1*S,*2*S,*8*R,*9*S,*10*R*)-1 at B3LYP/6-311G(d) level in methanol.

|  | (1*S,*2*S,*8*R,*9*S,*10*R*)-**1**-1 | | | (1*S,*2*S,*8*R,*9*S,*10*R*)-**1**-2 | | | (1*S,*2*S,*8*R,*9*S,*10*R*)-**1**-3 | | | (1*S,*2*S,*8*R,*9*S,*10*R*)-**1**-4 | | | (1*S,*2*S,*8*R,*9*S,*10*R*)-**1**-5 | | |
| --- | --- | --- | --- | --- | --- | --- | --- | --- | --- | --- | --- | --- | --- | --- | --- |
| C | 1.51097 | 1.80751 | 0.2926 | 1.47955 | 1.82581 | 0.33121 | 1.50694 | 1.78982 | 0.3166 | 1.53297 | 1.83791 | 0.25915 | 1.51151 | 1.85555 | 0.28014 |
| C | 0.21734 | 1.61128 | -0.4676 | 0.17336 | 1.75737 | -0.43528 | 0.19692 | 1.8605 | -0.45294 | 0.23356 | 1.68324 | -0.49677 | 0.18587 | 1.83451 | -0.4487 |
| C | -0.87111 | 1.01695 | 0.40686 | -0.96957 | 1.1734 | 0.39353 | -0.97669 | 1.2598 | 0.32906 | -0.82109 | 1.00682 | 0.37579 | -0.9112 | 1.16599 | 0.3969 |
| C | -2.03695 | 0.48597 | -0.43224 | -1.91873 | 0.35379 | -0.47744 | -1.84715 | 0.33822 | -0.51858 | -1.98089 | 0.47458 | -0.45677 | -1.84936 | 0.35417 | -0.47474 |
| C | -2.72227 | -0.77858 | 0.12155 | -2.69878 | -0.74821 | 0.25082 | -2.67744 | -0.69303 | 0.27671 | -2.70749 | -0.74923 | 0.1399 | -2.67839 | -0.71212 | 0.25744 |
| C | -1.87582 | -2.03337 | -0.19941 | -1.74146 | -1.75038 | 0.93933 | -1.7676 | -1.66035 | 1.06974 | -1.89885 | -2.04654 | -0.13561 | -1.76205 | -1.72278 | 1.00345 |
| C | -0.45303 | -2.01379 | 0.28836 | -0.47966 | -2.04856 | 0.17511 | -0.53324 | -2.06647 | 0.31529 | -0.46782 | -2.04727 | 0.33042 | -0.51231 | -2.08108 | 0.24754 |
| C | 0.59596 | -1.82695 | -0.51065 | 0.72895 | -1.6575 | 0.57831 | 0.69339 | -1.65866 | 0.63558 | 0.56773 | -1.87056 | -0.48827 | 0.70744 | -1.70061 | 0.62582 |
| C | 2.0619 | -1.73881 | -0.13973 | 2.05053 | -1.79034 | -0.14732 | 1.96935 | -1.84483 | -0.15517 | 2.03859 | -1.76984 | -0.14339 | 2.00563 | -1.83214 | -0.13944 |
| C | 2.66316 | -0.41582 | -0.71069 | 2.43128 | -0.41967 | -0.78146 | 2.33297 | -0.4871 | -0.8283 | 2.61457 | -0.43762 | -0.7211 | 2.34254 | -0.46067 | -0.79849 |
| C | 2.6029 | 0.82419 | 0.15913 | 2.5036 | 0.77636 | 0.14273 | 2.47472 | 0.69635 | 0.10027 | 2.57836 | 0.79969 | 0.15455 | 2.47095 | 0.74128 | 0.11318 |
| O | 2.65809 | 2.08875 | -0.54621 | 2.63494 | 2.06174 | -0.51167 | 2.67393 | 1.98669 | -0.52392 | 2.71063 | 2.05707 | -0.55013 | 2.67782 | 2.00485 | -0.56004 |
| O | -0.30184 | 2.84767 | -0.98504 | -0.24986 | 3.05157 | -0.89359 | -0.12338 | 3.22378 | -0.75856 | -0.21657 | 2.97017 | -0.93962 | -0.16833 | 3.17731 | -0.79968 |
| O | -3.05455 | 1.49336 | -0.56102 | -2.88038 | 1.18696 | -1.14253 | -2.69472 | 1.06197 | -1.42467 | -2.87857 | 1.57614 | -0.64082 | -2.67883 | 1.2853 | -1.17838 |
| O | -3.9405 | -0.96735 | -0.61627 | -3.40632 | -1.4947 | -0.75766 | -3.36735 | -1.49502 | -0.69667 | -3.91691 | -0.81811 | -0.64628 | -3.35022 | -1.37395 | -0.83783 |
| C | 2.30053 | -1.84621 | 1.37067 | 3.12701 | -2.22818 | 0.86408 | 3.09266 | -2.29272 | 0.79939 | 2.30535 | -1.88294 | 1.36174 | 3.12267 | -2.25123 | 0.83436 |
| C | 2.77702 | -2.91054 | -0.8472 | 1.98795 | -2.81874 | -1.28428 | 1.82116 | -2.88737 | -1.27033 | 2.75234 | -2.93092 | -0.86969 | 1.91351 | -2.86952 | -1.2665 |
| C | -3.05562 | -0.6566 | 1.60964 | -3.69634 | -0.18541 | 1.26334 | -3.69734 | -0.03383 | 1.20569 | -3.06255 | -0.56432 | 1.61089 | -3.70552 | -0.10977 | 1.21015 |
| C | 1.4334 | 2.66187 | 1.53503 | 1.46483 | 2.64033 | 1.60143 | 1.54514 | 2.56006 | 1.6153 | 1.4768 | 2.69604 | 1.50138 | 1.55013 | 2.68303 | 1.54308 |
| H | 3.2875 | 0.79815 | 1.00539 | 3.20177 | 0.6663 | 0.97115 | 3.16718 | 0.5358 | 0.92527 | 3.23994 | 0.74123 | 1.01757 | 3.15844 | 0.60324 | 0.94658 |
| H | 0.41536 | 0.93894 | -1.30642 | 0.3403 | 1.12916 | -1.31242 | 0.32649 | 1.31947 | -1.39339 | 0.42646 | 1.05913 | -1.37549 | 0.32129 | 1.26747 | -1.37343 |
| H | -0.43292 | 0.23815 | 1.02198 | -0.56098 | 0.55478 | 1.18742 | -0.59912 | 0.70154 | 1.18327 | -0.35311 | 0.2118 | 0.94479 | -0.46344 | 0.52855 | 1.15301 |
| H | -1.25336 | 1.78353 | 1.08515 | -1.51644 | 1.98743 | 0.87539 | -1.5748 | 2.07722 | 0.74317 | -1.21845 | 1.72926 | 1.09286 | -1.47432 | 1.93898 | 0.92618 |
| H | -1.68148 | 0.23341 | -1.43897 | -1.32907 | -0.16288 | -1.24236 | -1.20703 | -0.24449 | -1.18089 | -1.61403 | 0.15955 | -1.44304 | -1.25333 | -0.19938 | -1.20843 |
| H | -2.40976 | -2.89415 | 0.21848 | -1.48653 | -1.37344 | 1.9328 | -1.4896 | -1.19862 | 2.02036 | -2.44896 | -2.87688 | 0.32284 | -1.49634 | -1.31312 | 1.98175 |
| H | -1.89097 | -2.1479 | -1.2864 | -2.32455 | -2.66466 | 1.0922 | -2.38514 | -2.53213 | 1.31043 | -1.93127 | -2.20194 | -1.21707 | -2.37038 | -2.61394 | 1.19669 |
| H | -0.30421 | -2.11621 | 1.36084 | -0.60189 | -2.56385 | -0.77385 | -0.69739 | -2.65194 | -0.58513 | -0.30305 | -2.13897 | 1.40144 | -0.65618 | -2.60558 | -0.69323 |
| H | 0.40863 | -1.72732 | -1.58106 | 0.8015 | -1.13601 | 1.53321 | 0.81366 | -1.06251 | 1.54057 | 0.36174 | -1.7764 | -1.55565 | 0.8043 | -1.16726 | 1.57194 |
| H | 3.72823 | -0.58508 | -0.90553 | 3.42262 | -0.52394 | -1.2394 | 3.29305 | -0.60877 | -1.34461 | 3.67552 | -0.59991 | -0.94449 | 3.30617 | -0.56443 | -1.31279 |
| H | 2.21594 | -0.20235 | -1.68395 | 1.7409 | -0.21242 | -1.59995 | 1.59371 | -0.27116 | -1.60106 | 2.14129 | -0.22252 | -1.68137 | 1.60374 | -0.25983 | -1.57539 |
| H | 0.30662 | 3.16837 | -1.66175 | 0.41725 | 3.37595 | -1.51168 | 0.58592 | 3.56979 | -1.31484 | -1.16928 | 2.89365 | -1.09375 | -1.04667 | 3.12187 | -1.19922 |
| H | -2.62638 | 2.26666 | -0.95175 | -2.39626 | 1.908 | -1.5654 | -3.11491 | 1.78748 | -0.94735 | -3.67832 | 1.20698 | -1.04172 | -3.25393 | 0.7615 | -1.75151 |
| H | -4.3028 | -0.08064 | -0.75548 | -3.81504 | -0.83908 | -1.33898 | -3.69484 | -0.87168 | -1.3612 | -4.53312 | -1.42715 | -0.22392 | -4.05468 | -1.92673 | -0.47932 |
| H | 1.95506 | -2.81014 | 1.75181 | 2.90035 | -3.22281 | 1.25689 | 3.20845 | -1.612 | 1.6461 | 3.37504 | -1.7946 | 1.56863 | 4.09231 | -2.27259 | 0.32889 |
| H | 1.78078 | -1.06196 | 1.92617 | 3.18877 | -1.54568 | 1.71535 | 4.05121 | -2.34545 | 0.27528 | 1.97385 | -2.85156 | 1.74359 | 2.92681 | -3.2504 | 1.23232 |
| H | 3.36697 | -1.76474 | 1.5964 | 4.11225 | -2.26738 | 0.39102 | 2.8737 | -3.28401 | 1.20465 | 1.79036 | -1.1055 | 1.93105 | 3.20107 | -1.56966 | 1.68483 |
| H | 3.84654 | -2.90662 | -0.61818 | 1.70318 | -3.80327 | -0.9052 | 1.06757 | -2.59208 | -2.00443 | 2.62039 | -2.85805 | -1.95307 | 2.87586 | -2.96216 | -1.77658 |
| H | 2.36022 | -3.86785 | -0.52484 | 1.26651 | -2.53019 | -2.0525 | 2.76865 | -3.0106 | -1.80131 | 3.82563 | -2.91751 | -0.65963 | 1.64359 | -3.85218 | -0.87187 |
| H | 2.66485 | -2.84253 | -1.93311 | 2.96436 | -2.91115 | -1.76701 | 1.53402 | -3.85973 | -0.86247 | 2.35091 | -3.8942 | -0.54541 | 1.1694 | -2.58959 | -2.01604 |
| H | -3.6484 | -1.51993 | 1.91809 | -4.23641 | -1.0042 | 1.74408 | -3.2183 | 0.60454 | 1.9524 | -2.17413 | -0.54298 | 2.24452 | -4.36383 | 0.57162 | 0.67069 |
| H | -2.16379 | -0.61271 | 2.23834 | -3.19588 | 0.39996 | 2.03918 | -4.40355 | 0.57517 | 0.63543 | -3.6092 | 0.36988 | 1.75041 | -4.31429 | -0.89647 | 1.66599 |
| H | -3.64325 | 0.24622 | 1.79019 | -4.41837 | 0.46115 | 0.76273 | -4.27108 | -0.80185 | 1.72869 | -3.69249 | -1.38906 | 1.95697 | -3.22148 | 0.44451 | 2.01731 |
| H | 2.43335 | 2.84717 | 1.92936 | 2.46794 | 2.70518 | 2.02494 | 1.25458 | 3.59801 | 1.44377 | 2.47438 | 2.81099 | 1.92743 | 2.5477 | 2.66184 | 1.98416 |
| H | 0.9666 | 3.62139 | 1.30211 | 1.10834 | 3.6512 | 1.39206 | 0.85682 | 2.13369 | 2.34926 | 1.08472 | 3.68404 | 1.25348 | 1.28835 | 3.71815 | 1.31664 |
| H | 0.84082 | 2.1761 | 2.31364 | 0.80316 | 2.19694 | 2.34928 | 2.55018 | 2.54263 | 2.03875 | 0.82714 | 2.25425 | 2.26055 | 0.83791 | 2.30978 | 2.2831 |

## Table S15. Optimized cartesian coordinates of conformers of (1*R,*2*S,*8*R,*9*S,*10*R*)-1 at B3LYP/6-311G(d) level in methanol.

|  | (1*R,*2*S,*8*R,*9*S,*10*R*)-1-1 | | | (1*R,*2*S,*8*R,*9*S,*10*R*)-1-2 | | | (1*R,*2*S,*8*R,*9*S,*10*R*)-1-3 | | |
| --- | --- | --- | --- | --- | --- | --- | --- | --- | --- |
| C | 1.06723 | 1.90726 | 0.12782 | 1.20563 | 1.84391 | 0.23022 | -1.55728 | -1.67866 | 0.3664 |
| C | -0.31487 | 1.38429 | -0.22246 | -0.1861 | 1.43328 | -0.21866 | -0.22899 | -1.51334 | -0.34686 |
| C | -1.12829 | 0.96251 | 0.99607 | -1.13387 | 1.1165 | 0.93548 | 0.91968 | -1.22565 | 0.60594 |
| C | -2.46971 | 0.28487 | 0.70156 | -2.4607 | 0.46363 | 0.53689 | 2.24727 | -0.86151 | -0.10713 |
| C | -2.45201 | -1.1128 | 0.01742 | -2.42566 | -1.00235 | 0.02126 | 2.83957 | 0.51963 | 0.23677 |
| C | -1.37593 | -2.06649 | 0.58036 | -1.57924 | -1.96347 | 0.88043 | 1.98759 | 1.71004 | -0.2722 |
| C | -0.00779 | -1.88415 | -0.00452 | -0.08247 | -1.7529 | 0.94511 | 0.58549 | 1.82175 | 0.25583 |
| C | 1.10492 | -1.60622 | 0.66853 | 0.7287 | -1.77015 | -0.10848 | -0.4915 | 1.74907 | -0.52209 |
| C | 2.4704 | -1.38999 | 0.02591 | 2.22136 | -1.53377 | -0.13932 | -1.95157 | 1.80759 | -0.13294 |
| C | 2.42133 | -0.15961 | -0.91965 | 2.48269 | -0.21967 | -0.93316 | -2.66275 | 0.53931 | -0.6928 |
| C | 2.29777 | 1.18148 | -0.24043 | 2.40276 | 1.06534 | -0.14275 | -2.64918 | -0.70549 | 0.16885 |
| O | 1.82341 | 2.27803 | -1.05615 | 2.03816 | 2.25105 | -0.88725 | -2.64908 | -1.96597 | -0.54434 |
| O | -1.06022 | 2.45424 | -0.85668 | -0.78526 | 2.54926 | -0.92575 | 0.11145 | -2.72817 | -1.05085 |
| O | -3.30868 | 1.13722 | -0.09867 | -3.12711 | 1.24056 | -0.47631 | 2.14568 | -0.89816 | -1.53717 |
| O | -2.19484 | -0.93955 | -1.38403 | -1.90819 | -1.00954 | -1.31666 | 4.11315 | 0.59572 | -0.43112 |
| C | 3.52551 | -1.19133 | 1.12602 | 2.85234 | -1.46742 | 1.25614 | -2.16299 | 1.93585 | 1.37949 |
| C | 2.86423 | -2.61648 | -0.81903 | 2.86056 | -2.6945 | -0.92713 | -2.56747 | 3.03475 | -0.83754 |
| C | -3.83786 | -1.74481 | 0.19475 | -3.86892 | -1.52811 | -0.01269 | 3.14725 | 0.62893 | 1.72385 |
| C | 1.13335 | 2.93261 | 1.23473 | 1.28669 | 2.78453 | 1.40894 | -1.56635 | -2.54508 | 1.60332 |
| H | 3.17285 | 1.46195 | 0.34362 | 3.26383 | 1.23788 | 0.50101 | -3.38236 | -0.69083 | 0.97379 |
| H | -0.2355 | 0.56056 | -0.92769 | -0.12525 | 0.58716 | -0.8989 | -0.314 | -0.71031 | -1.07694 |
| H | -0.52299 | 0.3145 | 1.62834 | -0.6288 | 0.49383 | 1.67163 | 0.60984 | -0.43394 | 1.28462 |
| H | -1.34526 | 1.8592 | 1.58452 | -1.37015 | 2.0603 | 1.43656 | 1.08176 | -2.11429 | 1.21861 |
| H | -2.97365 | 0.1554 | 1.66559 | -3.09911 | 0.47019 | 1.42755 | 3.01172 | -1.58595 | 0.19763 |
| H | -1.3528 | -1.97546 | 1.67089 | -1.99153 | -1.93776 | 1.8931 | 2.55896 | 2.61121 | -0.02095 |
| H | -1.7074 | -3.08998 | 0.36676 | -1.78427 | -2.97208 | 0.50541 | 1.95717 | 1.64654 | -1.36221 |
| H | 0.03494 | -1.98253 | -1.08572 | 0.33314 | -1.58362 | 1.93506 | 0.47104 | 1.9546 | 1.32913 |
| H | 1.05537 | -1.49325 | 1.75114 | 0.28698 | -1.9307 | -1.0888 | -0.33233 | 1.62765 | -1.59467 |
| H | 3.34915 | -0.13277 | -1.50411 | 3.49409 | -0.25952 | -1.35386 | -3.71845 | 0.78057 | -0.8587 |
| H | 1.6107 | -0.28706 | -1.63917 | 1.80221 | -0.16682 | -1.78643 | -2.25335 | 0.29821 | -1.67673 |
| H | -0.63679 | 2.64647 | -1.70233 | -0.25156 | 2.71063 | -1.71394 | -0.60499 | -2.90311 | -1.67504 |
| H | -2.73146 | 1.79934 | -0.51747 | -2.49357 | 1.89906 | -0.81037 | 1.69642 | -1.72565 | -1.77045 |
| H | -2.77525 | -0.21142 | -1.65116 | -2.36677 | -0.27951 | -1.75925 | 3.95592 | 0.28032 | -1.33174 |
| H | 4.50456 | -0.96044 | 0.69694 | 3.9269 | -1.27932 | 1.18222 | -1.70997 | 1.10671 | 1.92798 |
| H | 3.62759 | -2.10128 | 1.72378 | 2.71428 | -2.41229 | 1.78746 | -3.22999 | 1.95106 | 1.61695 |
| H | 3.25521 | -0.38083 | 1.80758 | 2.41808 | -0.67472 | 1.86918 | -1.72665 | 2.86391 | 1.75668 |
| H | 2.86642 | -3.52159 | -0.20666 | 2.65521 | -3.65171 | -0.44072 | -2.06685 | 3.95299 | -0.52064 |
| H | 2.16715 | -2.77262 | -1.64555 | 2.46489 | -2.7433 | -1.94576 | -2.46962 | 2.95536 | -1.92408 |
| H | 3.8648 | -2.48859 | -1.24289 | 3.94528 | -2.57009 | -0.99241 | -3.63165 | 3.1244 | -0.60094 |
| H | -4.0213 | -2.012 | 1.23959 | -4.50465 | -0.84766 | -0.58194 | 2.24298 | 0.66256 | 2.33261 |
| H | -4.61823 | -1.0502 | -0.12303 | -3.89291 | -2.50826 | -0.49328 | 3.75184 | -0.21928 | 2.05323 |
| H | -3.90919 | -2.65032 | -0.41141 | -4.2858 | -1.62429 | 0.9937 | 3.71396 | 1.54366 | 1.91037 |
| H | 2.12835 | 3.37764 | 1.27453 | 0.63785 | 3.64866 | 1.24781 | -1.02447 | -2.07323 | 2.42583 |
| H | 0.40488 | 3.72714 | 1.05883 | 0.97851 | 2.29328 | 2.33444 | -2.59235 | -2.72464 | 1.92689 |
| H | 0.92084 | 2.48186 | 2.20689 | 2.3098 | 3.14091 | 1.53453 | -1.09684 | -3.50901 | 1.39312 |

## Table S16. Optimized cartesian coordinates of conformers of (1*R,*2*R,*8*R,*9*S,*10*R*)-1 at B3LYP/6-311G(d) level in methanol.

|  | (1*R,*2*R,*8*R,*9*S,*10*R*)-1-1 | | | (1*R,*2*R,*8*R,*9*S,*10*R*)-1-2 | | | (1*R,*2*R,*8*R,*9*S,*10*R*)-1-3 | | | (1*R,*2*R,*8*R,*9*S,*10*R*)-1-4 | | | (1*R,*2*R,*8*R,*9*S,*10*R*)-1-5 | | | (1*R,*2*R,*8*R,*9*S,*10*R*)-1-6 | | |
| --- | --- | --- | --- | --- | --- | --- | --- | --- | --- | --- | --- | --- | --- | --- | --- | --- | --- | --- |
| C | -0.68136 | 2.03993 | -0.26016 | -1.07148 | 1.95157 | -0.079 | -0.71573 | 2.04508 | -0.26109 | -1.54252 | -1.67187 | 0.36465 | -0.6697 | 2.04987 | -0.261 | 0.59214 | 2.0385 | 0.24018 |
| C | 0.24152 | 1.86874 | 0.95377 | 0.29403 | 1.37199 | 0.24122 | 0.21315 | 1.90449 | 0.95651 | -0.21526 | -1.50151 | -0.35087 | 0.25226 | 1.8742 | 0.94586 | -0.32828 | 1.91013 | -0.98778 |
| C | 0.94234 | 0.5157 | 1.06598 | 1.06372 | 0.94757 | -0.99844 | 0.9138 | 0.54588 | 1.0819 | 0.94541 | -1.24854 | 0.59739 | 0.93666 | 0.50847 | 1.06625 | -1.01636 | 0.54773 | -1.15913 |
| C | 1.75469 | 0.11319 | -0.16311 | 2.40116 | 0.24183 | -0.7646 | 1.73971 | 0.14776 | -0.1352 | 2.27348 | -0.87045 | -0.11285 | 1.74117 | 0.09915 | -0.16502 | -1.67063 | 0.0362 | 0.11017 |
| C | 2.26915 | -1.35142 | -0.16681 | 2.43162 | -1.10262 | 0.01405 | 2.28271 | -1.31017 | -0.14676 | 2.82163 | 0.52698 | 0.24992 | 2.26137 | -1.36284 | -0.16818 | -2.2363 | -1.39505 | 0.07021 |
| C | 1.19942 | -2.30463 | -0.75139 | 1.40392 | -2.11308 | -0.5383 | 1.23095 | -2.28827 | -0.7259 | 1.96985 | 1.69768 | -0.29085 | 1.19013 | -2.31927 | -0.74562 | -1.14461 | -2.45141 | -0.24913 |
| C | -0.14612 | -2.22933 | -0.08878 | 0.01602 | -1.97244 | 0.01825 | -0.11903 | -2.22932 | -0.06857 | 0.56729 | 1.78384 | 0.23553 | -0.15519 | -2.23511 | -0.08339 | 0.20502 | -2.11944 | 0.32212 |
| C | -1.16469 | -1.53624 | -0.59426 | -1.06496 | -1.60472 | -0.66563 | -1.14227 | -1.55078 | -0.58372 | -0.51146 | 1.75751 | -0.54141 | -1.16963 | -1.53753 | -0.5912 | 1.22842 | -1.77105 | -0.45447 |
| C | -2.49563 | -1.22677 | 0.05713 | -2.45028 | -1.38141 | -0.06991 | -2.48108 | -1.25588 | 0.05642 | -1.96857 | 1.80217 | -0.13923 | -2.49832 | -1.21683 | 0.05936 | 2.56206 | -1.17654 | -0.07644 |
| C | -2.46737 | 0.23906 | 0.59259 | -2.43261 | -0.15745 | 0.88765 | -2.47593 | 0.21385 | 0.58149 | -2.67173 | 0.53013 | -0.70066 | -2.4607 | 0.25012 | 0.59122 | 2.55295 | 0.32078 | -0.52465 |
| C | -1.93937 | 1.27075 | -0.37847 | -2.30413 | 1.2072 | 0.24912 | -1.95718 | 1.25048 | -0.39023 | -2.64568 | -0.71249 | 0.16292 | -1.9257 | 1.278 | -0.38009 | 1.86671 | 1.30083 | 0.39918 |
| O | -1.96339 | 2.64207 | 0.06162 | -1.83238 | 2.26699 | 1.11522 | -2.01318 | 2.62058 | 0.0502 | -2.63098 | -1.97483 | -0.54639 | -1.95036 | 2.64995 | 0.06049 | 1.86602 | 2.67664 | -0.03649 |
| O | 1.29318 | 2.85424 | 0.95247 | 1.10753 | 2.34992 | 0.92169 | 1.16058 | 2.97588 | 0.9944 | 0.10408 | -2.71129 | -1.07831 | 1.25021 | 2.92245 | 0.8679 | -1.29933 | 2.96283 | -0.98633 |
| O | 2.90761 | 0.9635 | -0.28963 | 3.3708 | 1.13738 | -0.20385 | 2.83027 | 1.07692 | -0.20116 | 2.18447 | -0.93407 | -1.53652 | 2.88735 | 0.95667 | -0.30146 | -2.70614 | 0.96748 | 0.46281 |
| O | 3.35583 | -1.42339 | -1.10268 | 3.71926 | -1.68407 | -0.28772 | 3.42983 | -1.31899 | -1.03331 | 2.83733 | 0.53791 | 1.68843 | 3.34415 | -1.43349 | -1.10854 | -2.68903 | -1.55702 | 1.43577 |
| C | -3.60915 | -1.38028 | -0.99659 | -3.46145 | -1.16394 | -1.20697 | -3.58423 | -1.4321 | -1.00474 | -2.16623 | 1.91771 | 1.3762 | -3.61272 | -1.36515 | -0.99419 | 2.87653 | -1.32392 | 1.417 |
| C | -2.80234 | -2.1525 | 1.2407 | -2.88609 | -2.60902 | 0.75233 | -2.7827 | -2.17868 | 1.24358 | -2.60195 | 3.02865 | -0.82816 | -2.81162 | -2.13756 | 1.24512 | 3.65826 | -1.89262 | -0.88937 |
| C | 2.76679 | -1.80582 | 1.2055 | 2.35548 | -0.95177 | 1.53655 | 2.82007 | -1.75009 | 1.2058 | 4.25204 | 0.65943 | -0.27818 | 2.76575 | -1.81355 | 1.20276 | -3.41519 | -1.54049 | -0.8869 |
| C | -0.06037 | 2.63365 | -1.50707 | -1.12227 | 3.02854 | -1.13556 | -0.10127 | 2.65395 | -1.50176 | -1.54601 | -2.53472 | 1.60416 | -0.05099 | 2.6437 | -1.50803 | -0.05146 | 2.59565 | 1.49363 |
| H | -2.31342 | 1.16235 | -1.39566 | -3.17731 | 1.51033 | -0.32613 | -2.32513 | 1.13264 | -1.40912 | -3.37986 | -0.70373 | 0.96712 | -2.29789 | 1.16896 | -1.39793 | 2.19603 | 1.20982 | 1.43253 |
| H | -0.3749 | 2.01469 | 1.84558 | 0.14579 | 0.52451 | 0.90434 | -0.41563 | 2.04166 | 1.83888 | -0.29734 | -0.68764 | -1.06885 | -0.35698 | 2.04026 | 1.83719 | 0.29412 | 2.09414 | -1.86538 |
| H | 1.61026 | 0.56795 | 1.92973 | 0.42458 | 0.31769 | -1.61491 | 1.57834 | 0.60074 | 1.94806 | 0.67131 | -0.47834 | 1.31382 | 1.60806 | 0.5475 | 1.92972 | -1.76538 | 0.65555 | -1.9487 |
| H | 0.19698 | -0.24181 | 1.28463 | 1.28706 | 1.84068 | -1.58875 | 0.17281 | -0.21829 | 1.29228 | 1.10517 | -2.16205 | 1.17383 | 0.18454 | -0.24202 | 1.28988 | -0.2854 | -0.17633 | -1.50557 |
| H | 1.14312 | 0.22332 | -1.06438 | 2.79098 | -0.01795 | -1.75337 | 1.13509 | 0.25274 | -1.04336 | 3.03917 | -1.57925 | 0.22665 | 1.12153 | 0.20372 | -1.06152 | -0.92484 | 0.02702 | 0.91051 |
| H | 1.10509 | -2.04796 | -1.8097 | 1.39133 | -2.04844 | -1.6311 | 1.11089 | -2.04452 | -1.78809 | 2.50873 | 2.61979 | -0.03238 | 1.09625 | -2.06953 | -1.80558 | -1.51747 | -3.41009 | 0.12935 |
| H | 1.60483 | -3.32074 | -0.70213 | 1.7974 | -3.10468 | -0.28825 | 1.65473 | -3.29602 | -0.67409 | 1.9567 | 1.63899 | -1.38116 | 1.59313 | -3.33592 | -0.68929 | -1.05984 | -2.55712 | -1.33423 |
| H | -0.24209 | -2.70691 | 0.88331 | -0.07929 | -2.17066 | 1.08399 | -0.21056 | -2.70555 | 0.90403 | 0.46491 | 1.8551 | 1.31463 | -0.25367 | -2.70834 | 0.89056 | 0.28351 | -2.06997 | 1.40482 |
| H | -1.01899 | -1.06289 | -1.5656 | -0.96694 | -1.4102 | -1.73278 | -0.99643 | -1.07902 | -1.55574 | -0.36034 | 1.68249 | -1.61969 | -1.02139 | -1.06869 | -1.56435 | 1.08309 | -1.83767 | -1.5341 |
| H | -3.49323 | 0.52703 | 0.85046 | -3.37661 | -0.14725 | 1.44585 | -3.50787 | 0.48672 | 0.83191 | -3.72951 | 0.76268 | -0.86637 | -3.48515 | 0.54507 | 0.8469 | 3.59566 | 0.65687 | -0.58517 |
| H | -1.90049 | 0.27184 | 1.52436 | -1.64518 | -0.29342 | 1.63104 | -1.91549 | 0.2607 | 1.51657 | -2.26009 | 0.29156 | -1.68433 | -1.89586 | 0.2819 | 1.52441 | 2.15301 | 0.39675 | -1.53931 |
| H | 0.91582 | 3.74107 | 0.9265 | 0.6808 | 2.55482 | 1.76286 | 1.93971 | 2.67948 | 0.49912 | -0.62184 | -2.8666 | -1.69683 | 1.6049 | 3.06399 | 1.75345 | -2.01682 | 2.66798 | -0.40499 |
| H | 2.66341 | 1.84079 | 0.04445 | 2.90312 | 1.67746 | 0.45283 | 3.50773 | 0.65667 | -0.75372 | 1.67945 | -1.73507 | -1.74899 | 2.61788 | 1.84247 | -0.00802 | -3.12982 | 0.61741 | 1.25851 |
| H | 3.86215 | -0.60737 | -0.9809 | 4.36936 | -0.99162 | -0.10557 | 3.11712 | -1.38946 | -1.94433 | 3.26233 | 1.35525 | 1.97472 | 3.84251 | -0.61086 | -0.99971 | -3.2768 | -2.32036 | 1.47894 |
| H | -4.58372 | -1.12561 | -0.57118 | -3.54269 | -2.06347 | -1.8232 | -3.613 | -2.46623 | -1.3579 | -3.23095 | 1.92662 | 1.62427 | -4.58564 | -1.10311 | -0.56947 | 2.09671 | -0.9053 | 2.05517 |
| H | -3.65479 | -2.41172 | -1.35566 | -3.16263 | -0.3422 | -1.86285 | -3.42134 | -0.79237 | -1.87555 | -1.72902 | 2.84453 | 1.75553 | -3.66518 | -2.39699 | -1.35121 | 3.81531 | -0.82116 | 1.66493 |
| H | -3.44331 | -0.73819 | -1.86516 | -4.45535 | -0.93688 | -0.81168 | -4.56508 | -1.1887 | -0.58743 | -1.70421 | 1.08665 | 1.91387 | -3.44238 | -0.72583 | -1.86394 | 2.9826 | -2.37983 | 1.67769 |
| H | -3.77455 | -1.90267 | 1.67362 | -3.90338 | -2.47623 | 1.13169 | -3.7612 | -1.93948 | 1.66808 | -2.10577 | 3.94912 | -0.51029 | -3.78197 | -1.87971 | 1.67756 | 3.49348 | -1.77361 | -1.96421 |
| H | -2.83209 | -3.19778 | 0.92323 | -2.86626 | -3.51155 | 0.13687 | -2.79606 | -3.22638 | 0.9329 | -2.51494 | 2.9577 | -1.91628 | -2.84894 | -3.18335 | 0.93015 | 4.64561 | -1.48624 | -0.65336 |
| H | -2.05679 | -2.06078 | 2.03414 | -2.22961 | -2.77304 | 1.61002 | -2.04424 | -2.07118 | 2.04167 | -3.66431 | 3.10814 | -0.57992 | -2.06532 | -2.04933 | 2.03827 | 3.66813 | -2.9628 | -0.66689 |
| H | 3.24034 | -2.78475 | 1.10891 | 3.16684 | -0.30989 | 1.88207 | 2.02245 | -1.83458 | 1.94484 | 4.6789 | 1.62654 | 0.00422 | 3.24137 | -2.79145 | 1.10598 | -3.11013 | -1.36614 | -1.92073 |
| H | 3.51 | -1.10329 | 1.59062 | 1.4192 | -0.52504 | 1.89287 | 3.30735 | -2.7218 | 1.10961 | 4.88176 | -0.12748 | 0.14329 | 3.5087 | -1.10863 | 1.58408 | -3.82764 | -2.55236 | -0.83047 |
| H | 1.96083 | -1.8857 | 1.9384 | 2.47863 | -1.93308 | 1.99984 | 3.55392 | -1.03022 | 1.57368 | 4.27684 | 0.57954 | -1.36573 | 1.96249 | -1.89445 | 1.93857 | -4.20167 | -0.82921 | -0.63437 |
| H | 0.87404 | 2.14358 | -1.77907 | -0.91831 | 2.62178 | -2.12848 | 0.17391 | 3.6898 | -1.29764 | -1.00281 | -2.0588 | 2.42333 | -0.76842 | 2.62477 | -2.32947 | -0.92687 | 2.02269 | 1.79829 |
| H | -0.7515 | 2.55869 | -2.34754 | -2.10949 | 3.49195 | -1.15132 | 0.80481 | 2.13238 | -1.81335 | -2.5707 | -2.71654 | 1.93088 | 0.23226 | 3.67914 | -1.31535 | 0.66597 | 2.60502 | 2.31526 |
| H | 0.16214 | 3.69147 | -1.34923 | -0.37971 | 3.80122 | -0.92331 | -0.81273 | 2.63787 | -2.32879 | -1.07445 | -3.49803 | 1.39549 | 0.84782 | 2.11165 | -1.82102 | -0.37843 | 3.61984 | 1.30753 |

## Table S17. Optimized cartesian coordinates of conformers of (1*S,*2*R,*8*R,*9*S,*10*S*)-1 at B3LYP/6-311G(d) level in methanol.

|  | (1*S,*2*R,*8*R,*9*S,*10*S)*-1-1 | | | (1*S,*2*R,*8*R,*9*S,*10*S)*-1-2 | | | (1*S,*2*R,*8*R,*9*S,*10*S)*-1-3 | | | (1*S,*2*R,*8*R,*9*S,*10*S)*-1-4 | | |
| --- | --- | --- | --- | --- | --- | --- | --- | --- | --- | --- | --- | --- |
| C | 0.99725 | 2.00629 | -0.05875 | -1.65291 | -1.78309 | 0.37783 | 0.99397 | 2.01403 | -0.03575 | 1.00949 | 2.00338 | -0.05406 |
| C | -0.33879 | 1.356 | 0.31485 | -0.27912 | -2.11551 | -0.2104 | -0.33772 | 1.36333 | 0.33921 | -0.34279 | 1.38538 | 0.32104 |
| C | -1.16438 | 1.04235 | -0.92824 | 0.86681 | -1.37344 | 0.51882 | -1.15148 | 1.04255 | -0.9178 | -1.16652 | 1.04668 | -0.92346 |
| C | -2.47337 | 0.28629 | -0.6917 | 1.73985 | -0.53945 | -0.41947 | -2.45352 | 0.27442 | -0.69145 | -2.47626 | 0.29417 | -0.67205 |
| C | -2.40068 | -1.15217 | -0.09461 | 2.6988 | 0.41955 | 0.30948 | -2.39144 | -1.15627 | -0.08965 | -2.40464 | -1.15518 | -0.09849 |
| C | -1.27361 | -2.03235 | -0.68509 | 1.91667 | 1.41067 | 1.2074 | -1.26013 | -2.03255 | -0.68036 | -1.27994 | -2.02871 | -0.70101 |
| C | 0.06236 | -1.87705 | -0.0191 | 0.73145 | 1.98409 | 0.48267 | 0.07717 | -1.8714 | -0.01827 | 0.05372 | -1.88007 | -0.02971 |
| C | 1.19732 | -1.52023 | -0.61326 | -0.53191 | 1.65652 | 0.74428 | 1.20969 | -1.51597 | -0.61746 | 1.19286 | -1.52788 | -0.61791 |
| C | 2.51999 | -1.28157 | 0.1065 | -1.74126 | 1.91674 | -0.12478 | 2.533 | -1.2713 | 0.09894 | 2.5115 | -1.29818 | 0.11169 |
| C | 2.43004 | 0.00515 | 0.97858 | -2.08752 | 0.57778 | -0.85037 | 2.43709 | 0.00917 | 0.97969 | 2.41464 | -0.01426 | 0.98617 |
| C | 2.27842 | 1.31891 | 0.22776 | -2.44339 | -0.57993 | 0.05096 | 2.27816 | 1.3272 | 0.23808 | 2.27909 | 1.29766 | 0.23202 |
| O | 1.70434 | 1.29886 | -1.09713 | -2.72951 | -1.85901 | -0.58702 | 1.69833 | 1.31372 | -1.08363 | 1.6992 | 1.28334 | -1.09205 |
| O | -1.12307 | 2.24768 | 1.14165 | -0.22944 | -1.85305 | -1.61385 | -1.03818 | 2.25868 | 1.21188 | -1.07682 | 2.25262 | 1.21826 |
| O | -3.35779 | 1.05152 | 0.14852 | 2.53823 | -1.4127 | -1.23254 | -3.31373 | 1.0169 | 0.21868 | -3.346 | 1.05381 | 0.18746 |
| O | -2.18197 | -1.05245 | 1.3212 | 3.55583 | -0.32418 | 1.1911 | -2.1681 | -1.06602 | 1.32575 | -2.18105 | -1.08454 | 1.31861 |
| C | 2.84647 | -2.45546 | 1.0482 | -2.91597 | 2.39499 | 0.74792 | 2.87103 | -2.44948 | 1.03137 | 2.82755 | -2.47593 | 1.05191 |
| C | 3.65277 | -1.14438 | -0.92269 | -1.47252 | 2.96532 | -1.21156 | 3.66094 | -1.11965 | -0.9335 | 3.65255 | -1.16046 | -0.90819 |
| C | -3.75514 | -1.82877 | -0.34009 | 3.55515 | 1.17795 | -0.70969 | -3.7431 | -1.8372 | -0.33944 | -3.76122 | -1.82372 | -0.35275 |
| C | 0.97938 | 3.50623 | -0.23067 | -1.92292 | -2.48994 | 1.68532 | 0.97867 | 3.51464 | -0.1997 | 1.03204 | 3.50525 | -0.23038 |
| H | 3.15344 | 1.96506 | 0.27469 | -3.20799 | -0.33985 | 0.78723 | 3.15309 | 1.97405 | 0.28486 | 3.16342 | 1.93123 | 0.27289 |
| H | -0.17361 | 0.43609 | 0.86817 | -0.14922 | -3.19593 | -0.07012 | -0.15259 | 0.43302 | 0.87216 | -0.19867 | 0.48209 | 0.90417 |
| H | -1.41818 | 1.98474 | -1.42688 | 0.44291 | -0.74152 | 1.29503 | -1.40978 | 1.98274 | -1.41919 | -1.42059 | 1.97718 | -1.44597 |
| H | -0.53673 | 0.4829 | -1.61926 | 1.51485 | -2.09097 | 1.02297 | -0.52142 | 0.4828 | -1.60819 | -0.53955 | 0.47291 | -1.60386 |
| H | -2.96533 | 0.19665 | -1.66619 | 1.1047 | 0.06121 | -1.06984 | -2.96053 | 0.19254 | -1.65752 | -2.98749 | 0.22206 | -1.63728 |
| H | -1.5942 | -3.07606 | -0.57991 | 1.5969 | 0.88435 | 2.10914 | -1.57977 | -3.07554 | -0.56945 | -1.60147 | -3.07322 | -0.60765 |
| H | -1.19935 | -1.84469 | -1.76091 | 2.61382 | 2.19274 | 1.52551 | -1.18929 | -1.84846 | -1.75663 | -1.20451 | -1.82861 | -1.77433 |
| H | 0.05173 | -2.05125 | 1.05307 | 0.95501 | 2.59908 | -0.38513 | 0.06952 | -2.03983 | 1.05462 | 0.03778 | -2.05646 | 1.04179 |
| H | 1.19183 | -1.31401 | -1.68081 | -0.73385 | 1.036 | 1.61698 | 1.20097 | -1.31428 | -1.68589 | 1.1949 | -1.32003 | -1.68511 |
| H | 1.61664 | -0.12507 | 1.6974 | -2.95845 | 0.75932 | -1.49271 | 1.62408 | -0.12877 | 1.69709 | 1.58983 | -0.14164 | 1.69199 |
| H | 3.34688 | 0.07623 | 1.57201 | -1.25845 | 0.30272 | -1.50029 | 3.35454 | 0.07999 | 1.57247 | 3.32308 | 0.05469 | 1.59249 |
| H | -0.81805 | 2.18207 | 2.0536 | -1.10998 | -2.05759 | -1.96097 | -1.9672 | 1.98262 | 1.17672 | -1.16998 | 3.12276 | 0.80954 |
| H | -2.79732 | 1.66702 | 0.65209 | 1.91218 | -1.88573 | -1.79702 | -3.83731 | 1.65127 | -0.28175 | -2.77128 | 1.58786 | 0.7616 |
| H | -2.80959 | -0.37757 | 1.61858 | 3.95477 | -1.02371 | 0.65656 | -2.83637 | -0.45605 | 1.66534 | -2.84067 | -0.45738 | 1.64659 |
| H | 2.09434 | -2.56725 | 1.83237 | -3.83808 | 2.45845 | 0.16291 | 3.83908 | -2.29226 | 1.51636 | 2.07 | -2.58756 | 1.83077 |
| H | 3.81428 | -2.30208 | 1.53481 | -2.70503 | 3.38778 | 1.15385 | 2.92066 | -3.3844 | 0.4676 | 3.79259 | -2.32713 | 1.54531 |
| H | 2.89072 | -3.3944 | 0.49083 | -3.09898 | 1.72996 | 1.59506 | 2.12186 | -2.57198 | 1.81679 | 2.87243 | -3.41329 | 0.49197 |
| H | 3.45258 | -0.33496 | -1.62633 | -1.15742 | 3.91502 | -0.77207 | 3.45214 | -0.30687 | -1.63068 | 3.45995 | -0.34925 | -1.61197 |
| H | 4.60666 | -0.9401 | -0.42774 | -0.69638 | 2.63896 | -1.90786 | 4.61513 | -0.9117 | -0.44063 | 4.60294 | -0.9593 | -0.40541 |
| H | 3.76479 | -2.06954 | -1.49551 | -2.38052 | 3.14736 | -1.7921 | 3.77774 | -2.0402 | -1.51293 | 3.76719 | -2.08446 | -1.48228 |
| H | -4.57094 | -1.1856 | -0.00325 | 4.26138 | 1.82482 | -0.18509 | -4.56333 | -1.19915 | -0.00439 | -3.91203 | -2.02011 | -1.41826 |
| H | -3.90377 | -2.04338 | -1.40251 | 4.11325 | 0.47838 | -1.33218 | -3.88701 | -2.05346 | -1.40186 | -3.8099 | -2.77344 | 0.18394 |
| H | -3.80176 | -2.76965 | 0.21228 | 2.94246 | 1.7971 | -1.37033 | -3.78477 | -2.77775 | 0.21308 | -4.57474 | -1.18367 | -0.00429 |
| H | 1.94981 | 3.84976 | -0.59261 | -1.19284 | -2.19676 | 2.44425 | 1.9501 | 3.86122 | -0.55708 | 0.25837 | 3.83897 | -0.92937 |
| H | 0.21653 | 3.80664 | -0.95402 | -2.9187 | -2.24996 | 2.05958 | 0.21724 | 3.81968 | -0.9228 | 0.887 | 4.02167 | 0.72116 |
| H | 0.75839 | 4.0082 | 0.71201 | -1.8545 | -3.57328 | 1.55463 | 0.7526 | 4.00539 | 0.74711 | 1.99624 | 3.81349 | -0.63539 |

## Table S18. Optimized cartesian coordinates of conformers of (1*S,*2*S,*8*R,*9*S,*10*S*)-1 at B3LYP/6-311G(d) level in methanol.

|  | (1*S,*2*S,*8*R,*9*S,*10*S)*-1-1 | | | (1*S,*2*S,*8*R,*9*S,*10*S)*-1-2 | | |
| --- | --- | --- | --- | --- | --- | --- |
| C | -1.59956 | -1.81403 | 0.39782 | -1.51195 | -1.88941 | 0.33191 |
| C | -0.23762 | -2.13937 | -0.22087 | -0.18883 | -1.91639 | -0.42454 |
| C | 0.93529 | -1.42926 | 0.50041 | 0.87136 | -1.14105 | 0.369 |
| C | 1.72495 | -0.50033 | -0.41536 | 2.03028 | -0.60289 | -0.4617 |
| C | 2.68568 | 0.4664 | 0.29859 | 2.74789 | 0.62021 | 0.16478 |
| C | 1.91015 | 1.48969 | 1.16253 | 1.96364 | 1.92486 | -0.10958 |
| C | 0.70137 | 2.01669 | 0.44675 | 0.54276 | 1.95768 | 0.37998 |
| C | -0.5481 | 1.66462 | 0.74068 | -0.50542 | 1.81869 | -0.42819 |
| C | -1.77953 | 1.88783 | -0.10825 | -1.97247 | 1.73914 | -0.07054 |
| C | -2.10497 | 0.53986 | -0.82418 | -2.56325 | 0.42227 | -0.66755 |
| C | -2.4156 | -0.6234 | 0.08617 | -2.52185 | -0.81948 | 0.19846 |
| O | -2.6936 | -1.90636 | -0.54646 | -2.68258 | -2.09557 | -0.49108 |
| O | -0.21118 | -1.84734 | -1.61897 | -0.3228 | -1.38503 | -1.73796 |
| O | 2.51071 | -1.25446 | -1.35497 | 3.03326 | -1.63231 | -0.63254 |
| O | 3.3364 | 1.22736 | -0.73526 | 3.98549 | 0.79102 | -0.54086 |
| C | -2.95042 | 2.34 | 0.78326 | -2.22469 | 1.83543 | 1.43808 |
| C | -1.55706 | 2.93969 | -1.20268 | -2.67895 | 2.91711 | -0.77555 |
| C | 3.74237 | -0.2575 | 1.13273 | 3.04961 | 0.42258 | 1.65227 |
| C | -1.83853 | -2.52561 | 1.70933 | -1.49772 | -2.69341 | 1.61256 |
| H | -3.16775 | -0.39668 | 0.83931 | -3.19206 | -0.75283 | 1.05421 |
| H | -0.11524 | -3.2235 | -0.10534 | 0.114 | -2.97042 | -0.50751 |
| H | 0.54788 | -0.86399 | 1.34792 | 0.37765 | -0.31843 | 0.87648 |
| H | 1.61934 | -2.17566 | 0.90764 | 1.28248 | -1.79362 | 1.14161 |
| H | 1.03272 | 0.11784 | -0.98331 | 1.67543 | -0.29846 | -1.44671 |
| H | 1.62124 | 1.02384 | 2.10787 | 2.54304 | 2.74116 | 0.33659 |
| H | 2.6169 | 2.29095 | 1.40207 | 1.98387 | 2.07663 | -1.19211 |
| H | 0.90438 | 2.60555 | -0.443 | 0.39563 | 2.03917 | 1.45447 |
| H | -0.71709 | 1.05247 | 1.62716 | -0.31163 | 1.72226 | -1.4966 |
| H | -2.99265 | 0.69626 | -1.45014 | -3.627 | 0.59352 | -0.87114 |
| H | -1.28158 | 0.28562 | -1.48944 | -2.08765 | 0.22726 | -1.62627 |
| H | -1.09894 | -2.0401 | -1.95449 | -1.15293 | -1.741 | -2.08444 |
| H | 1.87316 | -1.63212 | -1.97594 | 2.8755 | -2.07683 | -1.47134 |
| H | 3.52478 | 0.58941 | -1.43899 | 4.29553 | -0.11138 | -0.71443 |
| H | -2.75693 | 3.33918 | 1.18225 | -3.29348 | 1.75826 | 1.65466 |
| H | -3.102 | 1.67393 | 1.63582 | -1.87657 | 2.79447 | 1.82947 |
| H | -3.8841 | 2.37867 | 0.21456 | -1.71342 | 1.04455 | 1.99218 |
| H | -1.2553 | 3.89735 | -0.77125 | -2.26398 | 3.87161 | -0.44222 |
| H | -0.78721 | 2.63018 | -1.91344 | -2.5558 | 2.8565 | -1.86073 |
| H | -2.48041 | 3.09887 | -1.76564 | -3.75085 | 2.91345 | -0.55748 |
| H | 3.29805 | -0.81263 | 1.96258 | 3.60847 | -0.50374 | 1.80625 |
| H | 4.29867 | -0.96032 | 0.50956 | 3.66245 | 1.25317 | 2.00847 |
| H | 4.44516 | 0.47024 | 1.54441 | 2.14512 | 0.37877 | 2.26269 |
| H | -2.83458 | -2.30498 | 2.09457 | -1.15252 | -3.71369 | 1.4236 |
| H | -1.75093 | -3.60751 | 1.57837 | -0.83147 | -2.24302 | 2.3519 |
| H | -1.10676 | -2.21822 | 2.46073 | -2.49919 | -2.7443 | 2.04156 |

## Table S19. Optimized cartesian coordinates of conformers of (1*R,*2*S,*8*R,*9*S,*10*S*)-1 at B3LYP/6-311G(d) level in methanol.

|  | (1*R,*2*S,*8*R,*9*S,*10*S)*-1-1 | | | (1*R,*2*S,*8*R,*9*S,*10*S)*-1-2 | | | (1*R,*2*S,*8*R,*9*S,*10*S)*-1-3 | | |
| --- | --- | --- | --- | --- | --- | --- | --- | --- | --- |
| C | 0.8586 | 1.91344 | 0.44866 | -1.19887 | 1.99331 | -0.08213 | 1.7208 | -1.75976 | -0.35821 |
| C | 0.01569 | 2.0199 | -0.83546 | 0.26785 | 1.7271 | 0.24232 | 0.41858 | -2.13981 | 0.35777 |
| C | -0.97907 | 0.87822 | -1.07589 | 1.0035 | 1.10791 | -0.94806 | -0.78336 | -1.45951 | -0.34205 |
| C | -1.76282 | 0.42925 | 0.15668 | 2.42182 | 0.55636 | -0.71698 | -1.89854 | -0.86902 | 0.53571 |
| C | -2.54769 | -0.88875 | -0.02018 | 2.58416 | -0.84398 | -0.0501 | -2.8504 | 0.07043 | -0.25676 |
| C | -1.65227 | -2.04831 | -0.53153 | 1.58198 | -1.90152 | -0.5873 | -2.11234 | 1.12726 | -1.13775 |
| C | -0.30785 | -2.08434 | 0.14258 | 0.19665 | -1.82638 | -0.01919 | -0.94938 | 1.78806 | -0.45819 |
| C | 0.82149 | -1.82758 | -0.51232 | -0.92114 | -1.65419 | -0.7191 | 0.31574 | 1.64549 | -0.84808 |
| C | 2.20387 | -1.57042 | 0.03108 | -2.31291 | -1.50502 | -0.11921 | 1.55665 | 1.99892 | -0.05758 |
| C | 2.54771 | -0.08091 | -0.28253 | -2.34889 | -0.29417 | 0.85285 | 2.01876 | 0.71012 | 0.69382 |
| C | 1.97649 | 0.96337 | 0.64905 | -2.32011 | 1.0779 | 0.21866 | 2.41168 | -0.46673 | -0.17007 |
| O | 2.23388 | 2.34256 | 0.26783 | -2.01472 | 2.19938 | 1.09781 | 2.86324 | -1.6718 | 0.52355 |
| O | 0.85261 | 2.08656 | -1.98996 | 0.39155 | 0.93955 | 1.42794 | 0.5052 | -1.8334 | 1.75585 |
| O | -2.65498 | 1.46387 | 0.58454 | 3.22087 | 1.50658 | -0.00801 | -1.41388 | -0.08321 | 1.62961 |
| O | -3.55361 | -0.57401 | -1.00322 | 2.48921 | -0.70971 | 1.37568 | -3.65681 | 0.75652 | 0.71241 |
| C | 2.32723 | -1.88147 | 1.52659 | -3.34037 | -1.33499 | -1.24946 | 2.65319 | 2.51447 | -1.00536 |
| C | 3.20392 | -2.44552 | -0.74989 | -2.6751 | -2.7636 | 0.69324 | 1.28439 | 3.06612 | 1.01173 |
| C | -3.21887 | -1.27323 | 1.2991 | 4.00751 | -1.33605 | -0.31296 | -3.81023 | -0.73033 | -1.12867 |
| C | 0.2235 | 2.55973 | 1.66122 | -1.40835 | 3.02257 | -1.1708 | 1.95078 | -2.52897 | -1.63597 |
| H | 2.23175 | 0.79606 | 1.69339 | -3.20957 | 1.28309 | -0.37508 | 3.09783 | -0.21062 | -0.97444 |
| H | -0.53715 | 2.96448 | -0.74499 | 0.72445 | 2.70339 | 0.44953 | 0.31413 | -3.22771 | 0.2798 |
| H | -1.68328 | 1.18632 | -1.85365 | 0.38806 | 0.34625 | -1.41999 | -0.39098 | -0.6717 | -0.97317 |
| H | -0.4071 | 0.05115 | -1.48295 | 1.11057 | 1.91424 | -1.67868 | -1.24121 | -2.19394 | -1.00827 |
| H | -1.07475 | 0.27174 | 0.98632 | 2.86773 | 0.46802 | -1.71263 | -2.51136 | -1.68436 | 0.94404 |
| H | -2.20167 | -2.98549 | -0.38371 | 1.55228 | -1.84194 | -1.68032 | -1.78161 | 0.66413 | -2.07205 |
| H | -1.52164 | -1.92389 | -1.60854 | 2.00748 | -2.88192 | -0.34133 | -2.88266 | 1.85911 | -1.40273 |
| H | -0.29758 | -2.22143 | 1.22109 | 0.13731 | -1.89998 | 1.06373 | -1.17326 | 2.31212 | 0.46473 |
| H | 0.75986 | -1.70128 | -1.59384 | -0.85377 | -1.57671 | -1.80396 | 0.51317 | 1.09686 | -1.77028 |
| H | 3.63714 | 0.03265 | -0.21694 | -3.28734 | -0.34169 | 1.41945 | 2.90136 | 0.96523 | 1.29394 |
| H | 2.27044 | 0.14864 | -1.312 | -1.53931 | -0.39859 | 1.56983 | 1.22901 | 0.42537 | 1.38417 |
| H | 1.68877 | 2.47369 | -1.68976 | -0.23686 | 1.29537 | 2.07151 | 1.45099 | -1.82722 | 1.97462 |
| H | -3.40675 | 1.42712 | -0.02384 | 3.19702 | 1.22504 | 0.9191 | -0.68386 | -0.58308 | 2.03298 |
| H | -4.24061 | -1.24968 | -0.97284 | 1.64593 | -0.26267 | 1.57855 | -3.07826 | 0.9083 | 1.47399 |
| H | 2.17334 | -2.948 | 1.70871 | -3.08569 | -0.50087 | -1.90837 | 3.59854 | 2.65563 | -0.47334 |
| H | 1.59774 | -1.33625 | 2.12853 | -4.34198 | -1.15585 | -0.84862 | 2.36092 | 3.47678 | -1.4341 |
| H | 3.32377 | -1.61997 | 1.89259 | -3.38247 | -2.23769 | -1.86523 | 2.83388 | 1.83029 | -1.83802 |
| H | 4.2259 | -2.2742 | -0.40041 | -1.99617 | -2.8985 | 1.53864 | 2.2099 | 3.32644 | 1.5322 |
| H | 2.97236 | -3.50597 | -0.62125 | -3.69375 | -2.69226 | 1.08634 | 0.8819 | 3.9762 | 0.56006 |
| H | 3.17212 | -2.22047 | -1.81983 | -2.61275 | -3.65728 | 0.06738 | 0.57179 | 2.71597 | 1.76162 |
| H | -2.47699 | -1.53784 | 2.0567 | 4.73266 | -0.56263 | -0.05743 | -4.50789 | -0.05508 | -1.62973 |
| H | -3.81822 | -0.44727 | 1.67983 | 4.20868 | -2.21947 | 0.29625 | -3.28038 | -1.29809 | -1.89598 |
| H | -3.86724 | -2.14243 | 1.15333 | 4.13908 | -1.60345 | -1.36402 | -4.38898 | -1.4248 | -0.51609 |
| H | 0.80858 | 2.36431 | 2.56116 | -0.79514 | 3.91009 | -0.99203 | 2.89666 | -2.24305 | -2.09728 |
| H | 0.18813 | 3.64206 | 1.50522 | -1.14359 | 2.61835 | -2.15023 | 1.97217 | -3.60381 | -1.43763 |
| H | -0.79974 | 2.22373 | 1.82128 | -2.4544 | 3.3297 | -1.20044 | 1.14843 | -2.33439 | -2.35247 |

## Table S20. Optimized cartesian coordinates of conformers of (1*R,*2*R,*8*R,*9*S,*10*S*)-1 at B3LYP/6-311G(d) level in methanol.

|  | (1*R,*2*R,*8*R,*9*S,*10*S)*-1-1 | | | (1*R,*2*R,*8*R,*9*S,*10*S)*-1-2 | | | (1*R,*2*R,*8*R,*9*S,*10*S)*-1-3 | | | (1*R,*2*R,*8*R,*9*S,*10*S)*-1-4 | | | (1*R,*2*R,*8*R,*9*S,*10*S)*-1-5 | | |
| --- | --- | --- | --- | --- | --- | --- | --- | --- | --- | --- | --- | --- | --- | --- | --- |
| C | -0.96991 | 1.91284 | -0.44448 | -0.9797 | 1.90878 | -0.44327 | -0.98299 | 1.90825 | -0.44859 | -1.00412 | 1.89787 | -0.45244 | 0.8723 | 1.90934 | 0.42072 |
| C | -0.03375 | 2.00679 | 0.77701 | -0.05859 | 2.00565 | 0.78874 | -0.06941 | 2.01251 | 0.78856 | -0.03187 | 2.00318 | 0.74429 | 0.01141 | 2.02088 | -0.8505 |
| C | 0.91229 | 0.81928 | 0.97362 | 0.89489 | 0.82549 | 0.98731 | 0.88577 | 0.835 | 0.99555 | 0.91329 | 0.81927 | 0.95158 | -1.01874 | 0.90557 | -1.07791 |
| C | 1.84429 | 0.50493 | -0.18954 | 1.82592 | 0.53227 | -0.18142 | 1.81906 | 0.53525 | -0.17061 | 1.9012 | 0.53879 | -0.17416 | -1.75972 | 0.4282 | 0.1576 |
| C | 2.58201 | -0.86762 | -0.10103 | 2.57968 | -0.82533 | -0.08579 | 2.58206 | -0.82192 | -0.07612 | 2.58511 | -0.85821 | -0.11216 | -2.55762 | -0.88184 | -0.01524 |
| C | 1.7025 | -1.99887 | -0.68548 | 1.71835 | -1.98624 | -0.64791 | 1.73261 | -1.98942 | -0.63454 | 1.69325 | -1.94815 | -0.7496 | -1.65854 | -2.08083 | -0.41119 |
| C | 0.34576 | -2.14516 | -0.06072 | 0.35643 | -2.14004 | -0.0346 | 0.36898 | -2.13998 | -0.02345 | 0.34516 | -2.12542 | -0.11468 | -0.30366 | -2.05648 | 0.23558 |
| C | -0.76559 | -1.67673 | -0.62468 | -0.75277 | -1.6897 | -0.61718 | -0.739 | -1.69201 | -0.60961 | -0.78012 | -1.64925 | -0.64258 | 0.80794 | -1.84084 | -0.46311 |
| C | -2.15303 | -1.60507 | -0.02529 | -2.14671 | -1.6206 | -0.03316 | -2.13495 | -1.626 | -0.03052 | -2.15437 | -1.6056 | -0.00959 | 2.20784 | -1.57109 | 0.02764 |
| C | -2.41592 | -0.14556 | 0.45611 | -2.42047 | -0.16038 | 0.43951 | -2.41807 | -0.16474 | 0.43346 | -2.42615 | -0.15363 | 0.49303 | 2.54471 | -0.08995 | -0.32675 |
| C | -2.07013 | 0.93194 | -0.54415 | -2.06957 | 0.91796 | -0.55865 | -2.06713 | 0.91142 | -0.56717 | -2.11492 | 0.92808 | -0.51228 | 1.99614 | 0.96501 | 0.60437 |
| O | -2.3362 | 2.30074 | -0.13924 | -2.35445 | 2.2838 | -0.15674 | -2.36123 | 2.27696 | -0.17176 | -2.3588 | 2.30058 | -0.10682 | 2.24383 | 2.34248 | 0.21161 |
| O | -0.781 | 2.12997 | 1.98538 | -0.82256 | 2.12015 | 1.98927 | -0.84054 | 2.12908 | 1.98411 | -0.75343 | 2.15533 | 1.96591 | 0.82934 | 2.06215 | -2.01905 |
| O | 2.80542 | 1.56613 | -0.36453 | 2.75267 | 1.61455 | -0.2549 | 2.7507 | 1.61339 | -0.24916 | 2.94634 | 1.5386 | -0.11989 | -2.63279 | 1.46984 | 0.60125 |
| O | 3.70908 | -0.79774 | -0.98505 | 3.68529 | -0.64883 | -1.00331 | 3.75725 | -0.69961 | -0.91763 | 3.73485 | -0.80232 | -0.96645 | -3.06891 | -1.08241 | 1.32548 |
| C | -3.17935 | -2.00202 | -1.10341 | -3.15989 | -2.02587 | -1.12047 | -3.1417 | -2.04194 | -1.11986 | -3.20106 | -2.00699 | -1.06651 | 2.37993 | -1.85454 | 1.52427 |
| C | -2.3211 | -2.53142 | 1.18534 | -2.32418 | -2.54257 | 1.17964 | -2.31196 | -2.54263 | 1.18635 | -2.28024 | -2.54604 | 1.19503 | 3.17999 | -2.46508 | -0.76696 |
| C | 3.07702 | -1.19026 | 1.30947 | 3.12129 | -1.10823 | 1.31008 | 3.12338 | -1.09872 | 1.31771 | 3.03342 | -1.2251 | 1.30355 | -3.7207 | -0.74233 | -0.99245 |
| C | -0.43948 | 2.57762 | -1.69263 | -0.43739 | 2.58307 | -1.68158 | -0.43646 | 2.58133 | -1.68574 | -0.51875 | 2.52597 | -1.73718 | 0.25382 | 2.55117 | 1.64295 |
| H | -2.4286 | 0.74513 | -1.55474 | -2.41336 | 0.72724 | -1.57377 | -2.40412 | 0.71511 | -1.58352 | -2.50642 | 0.74101 | -1.51058 | 2.27096 | 0.80544 | 1.64479 |
| H | 0.56024 | 2.91933 | 0.63614 | 0.53174 | 2.92149 | 0.65809 | 0.51926 | 2.92957 | 0.65806 | 0.56581 | 2.909 | 0.57338 | -0.51749 | 2.97918 | -0.7567 |
| H | 1.51292 | 1.03207 | 1.86532 | 1.50733 | 1.05002 | 1.86431 | 1.49724 | 1.06456 | 1.87179 | 1.49175 | 1.03214 | 1.85437 | -1.7386 | 1.25352 | -1.82375 |
| H | 0.31433 | -0.05447 | 1.21355 | 0.3009 | -0.05077 | 1.22651 | 0.29184 | -0.04034 | 1.23878 | 0.31491 | -0.06162 | 1.16007 | -0.48135 | 0.0706 | -1.51951 |
| H | 1.29576 | 0.48984 | -1.1315 | 1.25339 | 0.48484 | -1.11579 | 1.24556 | 0.48808 | -1.10554 | 1.41779 | 0.59323 | -1.15106 | -1.03244 | 0.21486 | 0.94781 |
| H | 1.59825 | -1.78599 | -1.75261 | 1.61806 | -1.79869 | -1.72003 | 1.61492 | -1.8209 | -1.7115 | 1.57812 | -1.68377 | -1.80444 | -2.21233 | -2.98867 | -0.14529 |
| H | 2.2745 | -2.92907 | -0.59976 | 2.30004 | -2.90875 | -0.53715 | 2.32053 | -2.90543 | -0.51763 | 2.26365 | -2.8828 | -0.71542 | -1.54075 | -2.0964 | -1.49819 |
| H | 0.30604 | -2.5963 | 0.92768 | 0.31141 | -2.57738 | 0.95968 | 0.3247 | -2.57513 | 0.97144 | 0.32553 | -2.60809 | 0.85919 | -0.27629 | -2.12385 | 1.31978 |
| H | -0.67669 | -1.2203 | -1.61119 | -0.65694 | -1.24458 | -1.60803 | -0.64194 | -1.24947 | -1.60148 | -0.71467 | -1.15938 | -1.6148 | 0.71586 | -1.77307 | -1.54818 |
| H | -3.48442 | -0.05029 | 0.6842 | -3.49187 | -0.06957 | 0.65609 | -3.49113 | -0.07872 | 0.64338 | -3.48982 | -0.07485 | 0.74784 | 3.63558 | 0.02463 | -0.29416 |
| H | -1.8819 | 0.03649 | 1.38773 | -1.89704 | 0.02703 | 1.37593 | -1.90143 | 0.02973 | 1.37215 | -1.87024 | 0.02972 | 1.41166 | 2.2372 | 0.1197 | -1.35186 |
| H | -1.65576 | 2.4593 | 1.72708 | -1.69633 | 2.44305 | 1.72038 | -1.71365 | 2.44873 | 1.70943 | -1.63258 | 2.48202 | 1.71973 | 1.67258 | 2.44845 | -1.73771 |
| H | 3.10385 | 1.8642 | 0.50382 | 3.49181 | 1.28432 | -0.78656 | 3.51751 | 1.2533 | -0.72116 | 2.88567 | 2.10361 | -0.89603 | -3.14021 | 1.08517 | 1.32995 |
| H | 3.98851 | 0.13101 | -0.97369 | 4.35788 | -1.31335 | -0.81681 | 3.49154 | -0.82653 | -1.8375 | 4.13562 | 0.06189 | -0.7877 | -3.79126 | -1.7202 | 1.28759 |
| H | -4.19915 | -1.91706 | -0.71812 | -4.18397 | -1.94451 | -0.74589 | -3.05937 | -1.41838 | -2.01366 | -3.15332 | -1.37164 | -1.95438 | 1.6694 | -1.3005 | 2.14037 |
| H | -3.02117 | -3.03665 | -1.41883 | -2.99388 | -3.06071 | -1.43134 | -4.16743 | -1.9646 | -0.74915 | -4.21255 | -1.93709 | -0.65691 | 3.38738 | -1.5859 | 1.85363 |
| H | -3.10291 | -1.37314 | -1.99398 | -3.07722 | -1.39895 | -2.01191 | -2.96884 | -3.0772 | -1.42546 | -3.03778 | -3.03748 | -1.39264 | 2.23304 | -2.91791 | 1.72913 |
| H | -2.1377 | -3.57329 | 0.91105 | -2.13391 | -3.58494 | 0.91183 | -3.33574 | -2.47496 | 1.56353 | -3.28974 | -2.49248 | 1.61107 | 4.21298 | -2.29149 | -0.45271 |
| H | -1.63822 | -2.2685 | 1.99656 | -1.651 | -2.27341 | 1.9969 | -2.11622 | -3.58547 | 0.92452 | -2.08842 | -3.58256 | 0.90651 | 2.94901 | -3.52205 | -0.61109 |
| H | -3.33901 | -2.459 | 1.57731 | -3.34646 | -2.4723 | 1.56056 | -1.64274 | -2.2661 | 2.0044 | -1.58247 | -2.28068 | 1.99269 | 3.1157 | -2.2603 | -1.83954 |
| H | 3.7105 | -0.38643 | 1.69478 | 2.31946 | -1.29927 | 2.02512 | 2.31875 | -1.27526 | 2.03231 | 3.61092 | -2.15104 | 1.26883 | -3.36627 | -0.54905 | -2.00694 |
| H | 2.25881 | -1.34613 | 2.01573 | 3.76467 | -1.99307 | 1.29423 | 3.76535 | -1.98106 | 1.29457 | 3.67294 | -0.44016 | 1.71516 | -4.30746 | -1.66576 | -1.01764 |
| H | 3.67978 | -2.09994 | 1.27772 | 3.70924 | -0.26066 | 1.66727 | 3.71429 | -0.25035 | 1.66862 | 2.19333 | -1.36974 | 1.9857 | -4.37383 | 0.07738 | -0.69277 |
| H | -1.10179 | 2.40546 | -2.54211 | -0.41609 | 3.66478 | -1.52032 | -1.05514 | 2.36927 | -2.55897 | -1.3054 | 2.50453 | -2.49248 | 0.8481 | 2.35062 | 2.53563 |
| H | -0.36639 | 3.65588 | -1.52465 | 0.58342 | 2.26813 | -1.89977 | -0.42177 | 3.66365 | -1.52797 | -0.23228 | 3.56702 | -1.56628 | 0.21619 | 3.63445 | 1.49333 |
| H | 0.55817 | 2.22108 | -1.9508 | -1.06265 | 2.3771 | -2.55157 | 0.58733 | 2.27092 | -1.8964 | 0.34898 | 2.00306 | -2.14327 | -0.7681 | 2.21168 | 1.80557 |

## Table S21. Optimized cartesian coordinates of conformers of (1*R*,2*R*,4*R*,5*R*,10*R*)-2 at B3LYP/6-311G(d) level in chloroform.

|  | (1*R*,2*R*,4*R*,5*R*,10*R*)-2-1 | | | (1*R*,2*R*,4*R*,5*R*,10*R*)-2-2 | | | (1*R*,2*R*,4*R*,5*R*,10*R*)-2-3 | | | (1*R*,2*R*,4*R*,5*R*,10*R*)-2-4 | | | (1*R*,2*R*,4*R*,5*R*,10*R*)-2-5 | | | (1*R*,2*R*,4*R*,5*R*,10*R*)-2-6 | | |
| --- | --- | --- | --- | --- | --- | --- | --- | --- | --- | --- | --- | --- | --- | --- | --- | --- | --- | --- |
| C | -1.18289 | -1.83404 | 0.69733 | -1.15214 | -1.84356 | 0.69677 | -0.6437 | -2.02883 | 0.42965 | -1.20001 | -1.94569 | 0.233 | -0.64405 | -1.97306 | 0.32818 | -1.28113 | -1.99396 | 0.40935 |
| C | 1.35088 | -0.1403 | 0.4929 | 1.36151 | -0.11602 | 0.49384 | 1.43662 | 0.25191 | 0.17913 | 1.37534 | -0.26393 | 0.40968 | 1.49334 | 0.26416 | 0.16227 | 1.28274 | -0.26809 | 0.45684 |
| C | 1.91391 | 1.27535 | 0.40474 | 1.90413 | 1.30678 | 0.40268 | 1.46797 | 1.77976 | 0.16674 | 1.77067 | 1.20667 | 0.54465 | 1.50781 | 1.78907 | 0.17816 | 1.74544 | 1.19175 | 0.51937 |
| C | 0.82943 | 2.36529 | 0.41787 | 0.79476 | 2.36002 | 0.41614 | 0.12323 | 2.4664 | 0.43456 | 0.59389 | 2.17236 | 0.72743 | 0.14823 | 2.42509 | 0.45331 | 0.61918 | 2.21438 | 0.66217 |
| C | -0.27699 | 2.10316 | -0.60091 | -0.29971 | 2.07746 | -0.60804 | -1.00068 | 1.98041 | -0.4743 | -0.49303 | 2.00961 | -0.33176 | -0.96978 | 1.92884 | -0.45763 | -0.46341 | 2.06259 | -0.39739 |
| C | -1.40034 | 1.23121 | -0.05901 | -1.42466 | 1.21205 | -0.06698 | -1.81419 | 0.84001 | 0.11902 | -1.65184 | 1.11993 | 0.10442 | -1.85479 | 0.85857 | 0.16093 | -1.56504 | 1.10761 | 0.007 |
| C | -2.11361 | 0.08569 | -0.78217 | -2.12621 | 0.05469 | -0.78214 | -2.3435 | -0.3905 | -0.60089 | -2.42425 | 0.10795 | -0.72898 | -2.47057 | -0.35681 | -0.51428 | -2.19661 | 0.0303 | -0.87234 |
| C | -2.45706 | -1.10499 | 0.16874 | -2.44255 | -1.13976 | 0.17367 | -2.15036 | -1.7077 | 0.21094 | -2.58124 | -1.28202 | -0.03841 | -2.17125 | -1.69892 | 0.22125 | -2.56549 | -1.24953 | -0.07436 |
| C | -0.41197 | -1.23207 | 1.84782 | -0.39071 | -1.22896 | 1.84687 | 0.07525 | -1.33194 | 1.55256 | -0.46751 | -1.54543 | 1.48396 | 0.09595 | -1.33742 | 1.47227 | -0.55722 | -1.48067 | 1.62811 |
| C | 0.73219 | -0.53763 | 1.8112 | 0.74533 | -0.52116 | 1.81115 | 1.0055 | -0.37204 | 1.4862 | 0.66944 | -0.85121 | 1.60483 | 1.04445 | -0.39441 | 1.44406 | 0.58005 | -0.77702 | 1.69404 |
| H | 0.62185 | -0.25866 | -0.29138 | 0.63499 | -0.24723 | -0.29101 | 0.83033 | -0.11777 | -0.64132 | 0.7854 | -0.39464 | -0.49096 | 0.90194 | -0.09254 | -0.67359 | 0.66987 | -0.41878 | -0.42647 |
| O | 1.43326 | 3.65329 | 0.26302 | 1.44347 | 3.61338 | 0.181 | 0.29516 | 3.8887 | 0.37751 | 1.08713 | 3.51701 | 0.79753 | 0.35396 | 3.83592 | 0.3026 | 1.2508 | 3.49858 | 0.60411 |
| C | 0.18102 | 2.0514 | -2.04638 | 0.17661 | 2.01922 | -2.04725 | -0.72891 | 2.13603 | -1.95782 | 0.0026 | 2.118 | -1.76178 | -0.66029 | 2.03774 | -1.93853 | 0.03458 | 2.21335 | -1.82318 |
| C | -1.63254 | 2.69852 | -0.32607 | -1.66364 | 2.67552 | -0.34846 | -2.41005 | 2.22136 | 0.00227 | -1.83463 | 2.61309 | -0.00742 | -2.37768 | 2.26265 | -0.00895 | -1.84471 | 2.58882 | -0.07112 |
| H | -1.26343 | 1.02777 | 0.99267 | -1.29693 | 1.02203 | 0.98851 | -1.49768 | 0.61468 | 1.12608 | -1.53527 | 0.79273 | 1.12741 | -1.56822 | 0.64754 | 1.18141 | -1.43335 | 0.77715 | 1.02545 |
| O | -1.45398 | -0.36634 | -1.96839 | -1.46883 | -0.3884 | -1.97219 | -1.71927 | -0.48798 | -1.88582 | -1.8023 | -0.02656 | -2.01057 | -2.04567 | -0.40481 | -1.8783 | -1.33948 | -0.41378 | -1.93371 |
| C | -3.33522 | -0.61762 | 1.32978 | -3.32397 | -0.66643 | 1.33807 | -2.92393 | -1.62447 | 1.53374 | -3.41346 | -1.13525 | 1.24289 | -2.85645 | -1.69549 | 1.59529 | -3.49737 | -0.90545 | 1.09446 |
| C | -3.26188 | -2.12945 | -0.6491 | -3.23292 | -2.18048 | -0.63779 | -2.7461 | -2.86316 | -0.61505 | -3.35222 | -2.20281 | -1.00198 | -2.78688 | -2.83925 | -0.61036 | -3.31469 | -2.19721 | -1.02728 |
| C | 1.45098 | -0.11852 | 3.06508 | 1.4577 | -0.09455 | 3.06617 | 1.69586 | 0.14061 | 2.72417 | 1.31669 | -0.62457 | 2.94609 | 1.74131 | 0.06027 | 2.70071 | 1.2304 | -0.46017 | 3.01565 |
| C | 3.48718 | -2.70926 | -1.14274 | 3.54384 | -2.64208 | -1.14528 | 4.5124 | -1.54038 | -0.938 | 4.33921 | -2.00479 | -1.02724 | 4.5777 | -1.50599 | -0.95473 | 4.16991 | -2.15161 | -0.95473 |
| C | 2.32285 | -1.7762 | -0.9591 | 2.35749 | -1.73826 | -0.95559 | 3.04125 | -1.22237 | -0.86119 | 3.08419 | -1.17116 | -1.01383 | 3.10567 | -1.19293 | -0.87813 | 2.95518 | -1.25996 | -0.95107 |
| O | 2.43916 | -1.07454 | 0.18056 | 2.46291 | -1.03425 | 0.18376 | 2.82836 | -0.14614 | -0.08167 | 2.60735 | -1.04606 | 0.23751 | 2.88768 | -0.12219 | -0.09185 | 2.48182 | -1.10327 | 0.29827 |
| O | 1.39254 | -1.67525 | -1.73433 | 1.42315 | -1.65686 | -1.72815 | 2.16894 | -1.85132 | -1.41333 | 2.57003 | -0.67278 | -1.98928 | 2.2352 | -1.82007 | -1.43519 | 2.46848 | -0.745 | -1.93191 |
| H | -0.52374 | -2.042 | -0.14704 | -0.49121 | -2.0363 | -0.14977 | -0.12492 | -1.88781 | -0.51702 | -0.58875 | -1.80703 | -0.65975 | -0.18405 | -1.73852 | -0.63042 | -0.60711 | -2.07168 | -0.44325 |
| H | -1.51452 | -2.81991 | 1.04125 | -1.46247 | -2.83681 | 1.03965 | -0.57917 | -3.10221 | 0.64724 | -1.37496 | -3.02582 | 0.31121 | -0.52419 | -3.05667 | 0.45397 | -1.58818 | -3.01926 | 0.64517 |
| H | 2.47063 | 1.34725 | -0.53536 | 2.45441 | 1.40073 | -0.53634 | 1.83857 | 2.09086 | -0.81566 | 2.31574 | 1.48546 | -0.36192 | 1.86529 | 2.13629 | -0.79408 | 2.29358 | 1.41239 | -0.39811 |
| H | 2.62974 | 1.47109 | 1.20536 | 2.61502 | 1.51532 | 1.20468 | 2.19222 | 2.14454 | 0.89836 | 2.46214 | 1.33473 | 1.38027 | 2.22195 | 2.15202 | 0.92093 | 2.44759 | 1.32452 | 1.34551 |
| H | 0.37451 | 2.4097 | 1.41094 | 0.32882 | 2.38155 | 1.41155 | -0.17325 | 2.285 | 1.47073 | 0.14346 | 2.01312 | 1.71047 | -0.14321 | 2.21949 | 1.49263 | 0.14444 | 2.09726 | 1.64607 |
| H | -3.07093 | 0.45785 | -1.15909 | -3.09257 | 0.41107 | -1.15129 | -3.42689 | -0.27376 | -0.74942 | -3.44465 | 0.49053 | -0.87743 | -3.5644 | -0.23936 | -0.49567 | -3.12481 | 0.42079 | -1.31156 |
| H | -0.82254 | -1.44998 | 2.8323 | -0.79978 | -1.45106 | 2.83112 | -0.17291 | -1.6984 | 2.54811 | -0.91428 | -1.91519 | 2.40573 | -0.15689 | -1.74063 | 2.45222 | -1.00443 | -1.77218 | 2.57758 |
| H | 1.94898 | 3.65065 | -0.552 | 0.75675 | 4.29022 | 0.14226 | 0.66189 | 4.11372 | -0.48565 | 1.57874 | 3.69916 | -0.01209 | -0.50826 | 4.26076 | 0.38795 | 0.55531 | 4.16442 | 0.66923 |
| H | 0.91218 | 1.26052 | -2.23016 | -0.65451 | 1.89901 | -2.73821 | 0.10306 | 1.51753 | -2.29871 | -0.8217 | 2.10217 | -2.46874 | -0.27416 | 3.04056 | -2.14132 | 0.59926 | 3.1447 | -1.90615 |
| H | -0.65265 | 1.87438 | -2.72117 | 0.70375 | 2.94656 | -2.2858 | -1.5943 | 1.85619 | -2.5523 | 0.53625 | 3.0671 | -1.8921 | 0.08819 | 1.31173 | -2.26096 | 0.69701 | 1.3988 | -2.12198 |
| H | 0.64024 | 3.00648 | -2.32353 | 0.86569 | 1.19108 | -2.22652 | -0.49379 | 3.18315 | -2.1809 | 0.67917 | 1.30631 | -2.03335 | -1.54573 | 1.87074 | -2.54534 | -0.78888 | 2.28209 | -2.53948 |
| H | -2.22669 | 2.96023 | -1.19593 | -2.24893 | 2.93009 | -1.226 | -3.15623 | 2.38059 | -0.76985 | -2.40949 | 2.98221 | -0.85108 | -3.10164 | 2.43697 | -0.79808 | -2.4313 | 2.95153 | -0.90929 |
| H | -1.76148 | 3.36474 | 0.52093 | -1.81498 | 3.33924 | 0.49812 | -2.57652 | 2.79229 | 0.91002 | -1.95212 | 3.19292 | 0.90228 | -2.53998 | 2.85805 | 0.88557 | -2.01798 | 3.12511 | 0.8573 |
| H | -0.57147 | -0.71191 | -1.77182 | -0.57675 | -0.71114 | -1.78075 | -2.1893 | -1.15607 | -2.39655 | -2.42928 | -0.45431 | -2.60346 | -2.61688 | -1.02283 | -2.34665 | -1.17521 | 0.3197 | -2.53225 |
| H | -4.2378 | -0.12828 | 0.95207 | -3.62124 | -1.51663 | 1.95873 | -3.98904 | -1.45382 | 1.34988 | -2.94916 | -0.47693 | 1.97848 | -2.6728 | -2.63876 | 2.11743 | -3.81264 | -1.81941 | 1.60549 |
| H | -2.82368 | 0.08967 | 1.98428 | -4.23772 | -0.19531 | 0.96393 | -2.56835 | -0.82365 | 2.18365 | -3.55745 | -2.10867 | 1.72026 | -3.93937 | -1.58436 | 1.48588 | -4.39881 | -0.3997 | 0.73562 |
| H | -3.6518 | -1.46346 | 1.94689 | -2.82272 | 0.05173 | 1.98878 | -2.83082 | -2.56173 | 2.08973 | -4.40369 | -0.7302 | 1.01427 | -2.50551 | -0.89115 | 2.24373 | -3.02968 | -0.26051 | 1.83965 |
| H | -4.15478 | -1.66717 | -1.08016 | -2.63688 | -2.57868 | -1.45921 | -2.15047 | -3.07901 | -1.50608 | -3.62541 | -3.13568 | -0.50299 | -2.24416 | -2.9988 | -1.54528 | -2.6798 | -2.50096 | -1.85934 |
| H | -2.66954 | -2.53496 | -1.46964 | -3.54113 | -3.01263 | 0.00109 | -2.77309 | -3.78173 | -0.024 | -4.27804 | -1.73188 | -1.34867 | -2.74986 | -3.77948 | -0.05501 | -3.64246 | -3.09521 | -0.49651 |
| H | -3.58697 | -2.95831 | -0.01433 | -4.13533 | -1.73519 | -1.06699 | -3.77156 | -2.64244 | -0.92911 | -2.75331 | -2.47116 | -1.8759 | -3.8374 | -2.63981 | -0.84823 | -4.20355 | -1.71058 | -1.44041 |
| H | 1.47886 | 0.96988 | 3.18033 | 0.98292 | -0.51665 | 3.95311 | 1.41878 | -0.44532 | 3.60183 | 2.36091 | -0.95144 | 2.93346 | 2.8276 | -0.01099 | 2.59186 | 1.24083 | 0.61453 | 3.22438 |
| H | 0.97309 | -0.53596 | 3.95246 | 2.50182 | -0.42438 | 3.05184 | 2.78319 | 0.09231 | 2.6119 | 1.32211 | 0.43415 | 3.2252 | 1.51803 | 1.10605 | 2.93666 | 0.71348 | -0.95513 | 3.83924 |
| H | 2.49168 | -0.45896 | 3.04934 | 1.47454 | 0.99413 | 3.18061 | 1.44816 | 1.1872 | 2.92987 | 0.79803 | -1.17403 | 3.73325 | 1.44474 | -0.54538 | 3.55851 | 2.27377 | -0.79084 | 3.02213 |
| H | 3.47983 | -3.46073 | -0.34968 | 4.46554 | -2.05709 | -1.13391 | 4.69774 | -2.23281 | -1.7566 | 4.6613 | -2.16999 | -2.05307 | 5.16396 | -0.59473 | -1.07712 | 4.98815 | -1.66002 | -0.42286 |
| H | 3.41973 | -3.19963 | -2.11104 | 3.59958 | -3.35407 | -0.31889 | 5.10154 | -0.63199 | -1.06777 | 5.1292 | -1.49019 | -0.47502 | 4.89283 | -1.97769 | -0.01994 | 3.95681 | -3.08636 | -0.43334 |
| H | 4.42738 | -2.16088 | -1.06324 | 3.4547 | -3.17745 | -2.08763 | 4.82732 | -2.0062 | -0.00022 | 4.16447 | -2.9609 | -0.53067 | 4.76641 | -2.19183 | -1.7781 | 4.47432 | -2.35531 | -1.97907 |

## Table S22. Optimized cartesian coordinates of conformers of (1*R,*2*R*,4*R*,5*R,*10*S*)-2 at B3LYP/6-311G(d) level in chloroform.

|  | (1*R,*2*R*,4*R*,5*R,*10*S*)-2-1 | | | (1*R,*2*R*,4*R*,5*R,*10*S*)-2-2 | | | (1*R,*2*R*,4*R*,5*R,*10*S*)-2-3 | | | (1*R,*2*R*,4*R*,5*R,*10*S*)-2-4 | | |
| --- | --- | --- | --- | --- | --- | --- | --- | --- | --- | --- | --- | --- |
| C | 0.4781 | -2.00304 | -0.20488 | 0.47513 | -1.98277 | -0.21141 | 0.48513 | -1.99494 | -0.20866 | 1.00326 | -1.96451 | -0.29807 |
| C | -1.53365 | 0.34844 | 0.00538 | -1.54659 | 0.36125 | -0.00339 | -1.53625 | 0.34641 | 0.01041 | -1.49098 | -0.20752 | 0.23551 |
| C | -1.35419 | 1.86895 | -0.0264 | -1.35712 | 1.87927 | -0.03066 | -1.35397 | 1.86638 | -0.00948 | -1.66623 | 1.31688 | 0.28958 |
| C | -0.10919 | 2.35739 | -0.77948 | -0.10392 | 2.33761 | -0.77493 | -0.11147 | 2.34979 | -0.7713 | -0.64904 | 2.10992 | -0.54314 |
| C | 1.22769 | 1.92062 | -0.18943 | 1.22255 | 1.89465 | -0.16993 | 1.22036 | 1.918 | -0.18255 | 0.79461 | 1.99229 | -0.0869 |
| C | 1.79197 | 0.62566 | -0.76783 | 1.81481 | 0.62571 | -0.7656 | 1.79665 | 0.63314 | -0.77102 | 1.60796 | 0.91546 | -0.80015 |
| C | 2.55503 | -0.47014 | -0.01845 | 2.57362 | -0.47788 | -0.0245 | 2.5628 | -0.46226 | -0.02485 | 2.67576 | 0.0063 | -0.18738 |
| C | 2.02428 | -1.89816 | -0.34748 | 2.02249 | -1.89974 | -0.34808 | 2.03086 | -1.89072 | -0.35135 | 2.46418 | -1.49365 | -0.55461 |
| C | -0.09622 | -1.42359 | 1.05409 | -0.09923 | -1.40285 | 1.0473 | -0.08966 | -1.42128 | 1.05265 | 0.42849 | -1.60192 | 1.0403 |
| C | -0.94387 | -0.39359 | 1.18644 | -0.95731 | -0.38154 | 1.17818 | -0.94518 | -0.39849 | 1.1888 | -0.63617 | -0.83443 | 1.31315 |
| H | -1.18096 | -0.07507 | -0.92958 | -1.19521 | -0.06471 | -0.93789 | -1.18129 | -0.07221 | -0.92604 | -1.13946 | -0.48181 | -0.75398 |
| O | -0.17793 | 3.78204 | -0.92538 | -0.19284 | 3.76729 | -0.811 | -0.10292 | 3.78244 | -0.80539 | -0.98503 | 3.50323 | -0.49835 |
| C | 1.46202 | 2.3796 | 1.23754 | 1.42162 | 2.33544 | 1.26848 | 1.43749 | 2.37179 | 1.24921 | 1.02299 | 2.42056 | 1.35107 |
| C | 2.40296 | 1.9558 | -1.13083 | 2.41913 | 1.9675 | -1.09493 | 2.39995 | 1.97027 | -1.11929 | 1.8402 | 2.37378 | -1.10329 |
| H | 1.16461 | 0.22643 | -1.55593 | 1.21007 | 0.23662 | -1.57631 | 1.17861 | 0.23502 | -1.56684 | 1.03593 | 0.41758 | -1.57386 |
| O | 2.63104 | -0.24428 | 1.39109 | 2.67019 | -0.2491 | 1.38211 | 2.64687 | -0.23574 | 1.38351 | 2.83728 | 0.19138 | 1.22007 |
| C | 2.39538 | -2.24815 | -1.79743 | 2.39285 | -2.26109 | -1.79563 | 2.4012 | -2.2432 | -1.80095 | 2.77393 | -1.69138 | -2.04718 |
| C | 2.70993 | -2.91 | 0.58283 | 2.69156 | -2.9175 | 0.58778 | 2.71654 | -2.90156 | 0.57992 | 3.44555 | -2.35091 | 0.25854 |
| C | -1.45505 | 0.02236 | 2.54349 | -1.47908 | 0.02533 | 2.53378 | -1.46077 | 0.00855 | 2.54678 | -1.10475 | -0.63057 | 2.73167 |
| C | -4.93571 | -1.11795 | -0.38418 | -4.95267 | -1.09512 | -0.3867 | -4.93418 | -1.12858 | -0.3857 | -4.86592 | -1.64073 | -0.40833 |
| C | -3.45354 | -0.96594 | -0.60268 | -3.46963 | -0.94981 | -0.60435 | -3.45313 | -0.96887 | -0.60645 | -3.55608 | -0.96586 | -0.71822 |
| O | -2.98469 | 0.12187 | 0.04289 | -2.99793 | 0.14098 | 0.03314 | -2.98725 | 0.11741 | 0.04383 | -2.80853 | -0.83617 | 0.39423 |
| O | -2.76656 | -1.71566 | -1.25528 | -2.78459 | -1.70733 | -1.25029 | -2.76463 | -1.71241 | -1.26453 | -3.20816 | -0.58301 | -1.81174 |
| H | 0.224 | -3.0695 | -0.23462 | 0.20622 | -3.04553 | -0.24802 | 0.23026 | -3.0612 | -0.24299 | 1.00031 | -3.05812 | -0.38617 |
| H | -0.00281 | -1.57256 | -1.08125 | 0.00471 | -1.54131 | -1.08807 | 0.00477 | -1.5604 | -1.08338 | 0.36476 | -1.60664 | -1.10577 |
| H | -1.37347 | 2.29191 | 0.9819 | -1.36805 | 2.30943 | 0.9719 | -1.35693 | 2.29176 | 0.99509 | -1.67151 | 1.68621 | 1.31599 |
| H | -2.2167 | 2.28602 | -0.55016 | -2.21235 | 2.307 | -0.55823 | -2.22985 | 2.27899 | -0.52032 | -2.65364 | 1.54242 | -0.12294 |
| H | -0.16595 | 1.98889 | -1.80793 | -0.15501 | 1.95212 | -1.804 | -0.17341 | 1.96853 | -1.80083 | -0.71607 | 1.75927 | -1.58231 |
| H | 3.60161 | -0.44673 | -0.3365 | 3.61623 | -0.46663 | -0.35635 | 3.6077 | -0.43962 | -0.34897 | 3.65102 | 0.28926 | -0.59489 |
| H | 0.21012 | -1.92333 | 1.97227 | 0.20919 | -1.90061 | 1.96594 | 0.22157 | -1.92113 | 1.96905 | 0.94143 | -2.04411 | 1.8931 |
| H | -0.26378 | 4.17402 | -0.04846 | 0.62657 | 4.09678 | -1.20026 | -0.95262 | 4.08066 | -1.14992 | -1.89658 | 3.60156 | -0.7968 |
| H | 0.75685 | 1.93513 | 1.94446 | 0.75813 | 1.81059 | 1.95963 | 2.45441 | 2.17877 | 1.5804 | 2.08181 | 2.47348 | 1.59044 |
| H | 2.46399 | 2.13375 | 1.57783 | 2.4424 | 2.17125 | 1.60331 | 1.2458 | 3.4451 | 1.30767 | 0.58027 | 3.40825 | 1.49453 |
| H | 1.34926 | 3.46698 | 1.29834 | 1.19306 | 3.40107 | 1.34374 | 0.7625 | 1.87739 | 1.95263 | 0.55532 | 1.74292 | 2.07012 |
| H | 3.36685 | 2.19111 | -0.69105 | 3.3702 | 2.21082 | -0.63325 | 3.35838 | 2.21145 | -0.67128 | 2.74819 | 2.82141 | -0.71256 |
| H | 2.26337 | 2.33936 | -2.13603 | 2.30104 | 2.34815 | -2.10548 | 2.26253 | 2.35998 | -2.12232 | 1.52596 | 2.76281 | -2.06589 |
| H | 1.72607 | -0.15636 | 1.71588 | 1.77131 | -0.15641 | 1.7217 | 1.74529 | -0.13173 | 1.71211 | 1.97127 | 0.07009 | 1.62832 |
| H | 3.48096 | -2.25537 | -1.93102 | 3.47855 | -2.28583 | -1.926 | 3.48671 | -2.25079 | -1.93511 | 3.81547 | -1.43889 | -2.26521 |
| H | 1.97527 | -1.54104 | -2.51683 | 1.98565 | -1.5506 | -2.51927 | 1.98088 | -1.53705 | -2.52123 | 2.13999 | -1.07459 | -2.68885 |
| H | 2.02095 | -3.24171 | -2.05863 | 2.00364 | -3.24962 | -2.05418 | 2.0265 | -3.23713 | -2.06053 | 2.61703 | -2.7343 | -2.33604 |
| H | 2.44708 | -2.75073 | 1.62801 | 2.39448 | -3.93377 | 0.31387 | 2.43135 | -3.92128 | 0.30597 | 3.38327 | -3.39679 | -0.05533 |
| H | 2.42622 | -3.92942 | 0.30641 | 3.78118 | -2.85458 | 0.51522 | 3.80499 | -2.82474 | 0.50252 | 4.47456 | -2.01433 | 0.10317 |
| H | 3.79838 | -2.83184 | 0.507 | 2.42786 | -2.75154 | 1.63171 | 2.45537 | -2.7396 | 1.62506 | 3.24776 | -2.29996 | 1.32858 |
| H | -2.54681 | -0.01955 | 2.57729 | -1.06432 | -0.60701 | 3.32012 | -2.55183 | -0.05014 | 2.58169 | -2.12779 | -0.99557 | 2.85419 |
| H | -1.06329 | -0.62798 | 3.32683 | -1.2357 | 1.06368 | 2.77783 | -1.05855 | -0.63697 | 3.32885 | -0.46505 | -1.16159 | 3.4381 |
| H | -1.17518 | 1.05012 | 2.79476 | -2.5688 | -0.05402 | 2.56962 | -1.19631 | 1.04007 | 2.79838 | -1.11298 | 0.42537 | 3.0176 |
| H | -5.10883 | -1.52634 | 0.61537 | -5.45363 | -0.12854 | -0.44472 | -5.3469 | -1.80479 | -1.13166 | -4.68694 | -2.61686 | 0.04685 |
| H | -5.4404 | -0.15305 | -0.43828 | -5.36696 | -1.78307 | -1.12096 | -5.10211 | -1.55415 | 0.60757 | -5.43022 | -1.04428 | 0.312 |
| H | -5.34792 | -1.8045 | -1.12087 | -5.1282 | -1.49911 | 0.61424 | -5.44255 | -0.16482 | -0.42188 | -5.44511 | -1.75832 | -1.32145 |

## Table S23. Optimized cartesian coordinates of conformers of (1*R,*2*R*,4*R*,5*S,*10*R*)-2 at B3LYP/6-311G(d) level in chloroform.

|  | (1*R,*2*R*,4*R*,5*S,*10*R*)-2-1 | | | (1*R,*2*R*,4*R*,5*S,*10*R*)-2-2 | | | (1*R,*2*R*,4*R*,5*S,*10*R*)-2-3 | | | (1*R,*2*R*,4*R*,5*S,*10*R*)-2-4 | | | (1*R,*2*R*,4*R*,5*S,*10*R*)-2-5 | | |
| --- | --- | --- | --- | --- | --- | --- | --- | --- | --- | --- | --- | --- | --- | --- | --- |
| C | -0.93711 | -2.01644 | 0.2029 | -0.52684 | -1.98498 | 0.27165 | -0.54103 | -1.99173 | 0.29474 | -0.54774 | -2.00763 | 0.35096 | -0.56261 | -2.01444 | 0.37194 |
| C | 1.51095 | -0.16975 | 0.35496 | 1.58244 | 0.26979 | 0.13457 | 1.57439 | 0.25182 | 0.13559 | 1.56255 | 0.24485 | 0.15823 | 1.55349 | 0.22665 | 0.1585 |
| C | 1.79569 | 1.32933 | 0.44092 | 1.57682 | 1.79505 | 0.1144 | 1.57581 | 1.77767 | 0.1034 | 1.59455 | 1.77021 | 0.12505 | 1.5931 | 1.75226 | 0.1141 |
| C | 0.55819 | 2.21144 | 0.64439 | 0.21548 | 2.43067 | 0.38569 | 0.21888 | 2.43668 | 0.38107 | 0.24782 | 2.43994 | 0.38876 | 0.25252 | 2.44879 | 0.38319 |
| C | -0.57134 | 1.92633 | -0.33659 | -0.92197 | 1.88632 | -0.46511 | -0.91776 | 1.90392 | -0.46861 | -0.89424 | 1.91014 | -0.46195 | -0.89127 | 1.93035 | -0.463 |
| C | -1.64927 | 0.97585 | 0.163 | -1.77727 | 0.81095 | 0.17709 | -1.77056 | 0.82253 | 0.17522 | -1.74591 | 0.82969 | 0.17899 | -1.73733 | 0.84216 | 0.18095 |
| C | -2.31498 | -0.06555 | -0.7234 | -2.32399 | -0.37915 | -0.5959 | -2.31096 | -0.36976 | -0.59867 | -2.25445 | -0.36442 | -0.60408 | -2.24009 | -0.35317 | -0.60421 |
| C | -2.37327 | -1.48636 | -0.08801 | -2.05634 | -1.7479 | 0.09592 | -2.06591 | -1.73634 | 0.1061 | -2.06558 | -1.71833 | 0.1416 | -2.07467 | -1.70564 | 0.1501 |
| C | -0.23893 | -1.54947 | 1.45108 | 0.17099 | -1.327 | 1.43027 | 0.15981 | -1.32709 | 1.44751 | 0.16041 | -1.32904 | 1.49258 | 0.14982 | -1.33123 | 1.5079 |
| C | 0.85102 | -0.78148 | 1.56266 | 1.11163 | -0.37527 | 1.415 | 1.10463 | -0.37981 | 1.42269 | 1.10318 | -0.37994 | 1.45327 | 1.09607 | -0.38617 | 1.45954 |
| H | 0.9291 | -0.36818 | -0.5369 | 1.01634 | -0.11144 | -0.70706 | 1.00199 | -0.13032 | -0.70134 | 0.97067 | -0.12884 | -0.66899 | 0.95556 | -0.14693 | -0.66439 |
| O | 0.9507 | 3.59046 | 0.63361 | 0.40656 | 3.83321 | 0.15998 | 0.31399 | 3.84583 | 0.13091 | 0.4703 | 3.83711 | 0.15605 | 0.38431 | 3.85276 | 0.1193 |
| C | -0.19629 | 1.94103 | -1.8055 | -0.68106 | 1.87949 | -1.96077 | -0.67404 | 1.89166 | -1.96397 | -0.65399 | 1.90193 | -1.95756 | -0.65214 | 1.91753 | -1.95893 |
| C | -1.93005 | 2.45689 | 0.02822 | -2.32199 | 2.21109 | 0.00242 | -2.31363 | 2.22299 | -0.00514 | -2.29643 | 2.22197 | 0.00775 | -2.28848 | 2.23394 | 0.00737 |
| H | -1.49987 | 0.65768 | 1.18496 | -1.49375 | 0.58776 | 1.19585 | -1.48767 | 0.59929 | 1.19386 | -1.46076 | 0.60413 | 1.19557 | -1.44943 | 0.61499 | 1.19616 |
| O | -3.62098 | 0.35913 | -1.14684 | -3.72011 | -0.21713 | -0.8959 | -3.70145 | -0.20174 | -0.92217 | -3.63222 | -0.12593 | -0.93415 | -3.61052 | -0.10866 | -0.96034 |
| C | -3.24288 | -1.49803 | 1.17588 | -2.79932 | -1.84999 | 1.43468 | -2.81985 | -1.81912 | 1.43988 | -2.84111 | -1.73907 | 1.46391 | -2.85996 | -1.70974 | 1.46672 |
| C | -2.99803 | -2.43064 | -1.12822 | -2.57628 | -2.84805 | -0.84406 | -2.59145 | -2.83797 | -0.82914 | -2.60799 | -2.83798 | -0.76502 | -2.6246 | -2.82339 | -0.75441 |
| C | 1.48515 | -0.49101 | 2.89705 | 1.77457 | 0.10272 | 2.68062 | 1.77088 | 0.10673 | 2.68335 | 1.77947 | 0.11437 | 2.70533 | 1.77579 | 0.11582 | 2.70671 |
| C | 4.5511 | -1.75564 | -1.0958 | 4.70447 | -1.4601 | -0.93695 | 4.68391 | -1.49504 | -0.9478 | 4.6239 | -1.53978 | -0.99954 | 4.60151 | -1.57511 | -1.01086 |
| C | 3.2289 | -1.03506 | -1.07848 | 3.23127 | -1.14828 | -0.90164 | 3.21286 | -1.17369 | -0.90935 | 3.16151 | -1.18316 | -0.94461 | 3.14132 | -1.21046 | -0.95065 |
| O | 2.79657 | -0.8574 | 0.18349 | 2.98443 | -0.10728 | -0.08366 | 2.97381 | -0.13523 | -0.08514 | 2.95055 | -0.17151 | -0.08221 | 2.93869 | -0.19934 | -0.08499 |
| O | 2.62875 | -0.65753 | -2.0595 | 2.38083 | -1.75214 | -1.51339 | 2.35775 | -1.76813 | -1.52345 | 2.29104 | -1.73408 | -1.57879 | 2.26573 | -1.75451 | -1.58333 |
| H | -0.32493 | -1.84794 | -0.68665 | -0.02342 | -1.76521 | -0.67083 | -0.02952 | -1.79152 | -0.64782 | -0.02185 | -1.84326 | -0.59112 | -0.02973 | -1.86921 | -0.56941 |
| H | -1.02371 | -3.10637 | 0.29096 | -0.40004 | -3.06528 | 0.41638 | -0.42972 | -3.07147 | 0.45537 | -0.46202 | -3.08419 | 0.54254 | -0.49352 | -3.08952 | 0.57781 |
| H | 2.2875 | 1.62295 | -0.49206 | 1.92344 | 2.12253 | -0.86905 | 1.92017 | 2.0955 | -0.88403 | 1.94833 | 2.07929 | -0.86185 | 1.94491 | 2.05147 | -0.87653 |
| H | 2.50483 | 1.53201 | 1.24636 | 2.29378 | 2.18303 | 0.84162 | 2.30816 | 2.15503 | 0.82477 | 2.32182 | 2.14726 | 0.84776 | 2.33572 | 2.11773 | 0.83103 |
| H | 0.17116 | 2.06151 | 1.65553 | -0.05259 | 2.27193 | 1.43954 | -0.04631 | 2.27983 | 1.43525 | -0.02695 | 2.29371 | 1.44273 | -0.01865 | 2.30944 | 1.43834 |
| H | -1.75832 | -0.16812 | -1.65866 | -1.85143 | -0.4302 | -1.57994 | -1.82168 | -0.432 | -1.57412 | -1.69025 | -0.45078 | -1.54157 | -1.66055 | -0.4489 | -1.53156 |
| H | -0.665 | -1.93415 | 2.37636 | -0.10721 | -1.72054 | 2.4071 | -0.11975 | -1.7103 | 2.42804 | -0.11098 | -1.70456 | 2.47821 | -0.12194 | -1.69772 | 2.4968 |
| H | 1.37418 | 3.77674 | -0.21292 | -0.45267 | 4.26012 | 0.26223 | 1.06876 | 4.18881 | 0.62353 | -0.38072 | 4.28139 | 0.25313 | 1.13924 | 4.18345 | 0.62002 |
| H | -1.07485 | 1.82002 | -2.44075 | -0.33037 | 2.86356 | -2.28005 | -1.58264 | 1.64138 | -2.51353 | 0.10228 | 1.17044 | -2.25712 | 0.12008 | 1.20226 | -2.25804 |
| H | 0.25293 | 2.90227 | -2.07567 | 0.07147 | 1.14422 | -2.2591 | -0.35174 | 2.88283 | -2.29022 | -1.5685 | 1.67238 | -2.5068 | -1.56336 | 1.66218 | -2.50228 |
| H | 0.51704 | 1.15589 | -2.06836 | -1.59449 | 1.65675 | -2.51405 | 0.09622 | 1.17345 | -2.2596 | -0.30952 | 2.88698 | -2.28085 | -0.33542 | 2.90926 | -2.289 |
| H | -2.56608 | 2.7783 | -0.788 | -3.07044 | 2.37338 | -0.76358 | -3.05883 | 2.38354 | -0.77494 | -3.05373 | 2.37046 | -0.75061 | -3.04665 | 2.37846 | -0.75101 |
| H | -2.0346 | 3.03862 | 0.93743 | -2.46079 | 2.80591 | 0.90038 | -2.44176 | 2.83432 | 0.88124 | -2.43843 | 2.81066 | 0.9093 | -2.41913 | 2.83687 | 0.89948 |
| H | -4.04076 | 0.8026 | -0.40072 | -4.14734 | 0.15287 | -0.11477 | -4.13138 | 0.20436 | -0.16079 | -3.88564 | -0.7477 | -1.62491 | -3.85194 | -0.72673 | -1.65862 |
| H | -3.26308 | -2.49774 | 1.61881 | -3.88005 | -1.79935 | 1.2824 | -2.62496 | -2.77649 | 1.93139 | -2.4777 | -0.99493 | 2.17484 | -2.79706 | -2.68933 | 1.94971 |
| H | -4.27378 | -1.22795 | 0.93563 | -2.52048 | -1.05968 | 2.13522 | -3.89825 | -1.74989 | 1.27898 | -2.75843 | -2.71892 | 1.94325 | -3.912 | -1.49029 | 1.27652 |
| H | -2.88427 | -0.80827 | 1.94291 | -2.58437 | -2.80623 | 1.91983 | -2.5326 | -1.02985 | 2.13812 | -3.89802 | -1.53634 | 1.2826 | -2.48978 | -0.96916 | 2.17785 |
| H | -3.12604 | -3.43404 | -0.71277 | -2.47837 | -3.83269 | -0.37864 | -2.50612 | -3.82022 | -0.3561 | -2.10944 | -2.83848 | -1.7399 | -2.12199 | -2.83291 | -1.72725 |
| H | -3.97167 | -2.06056 | -1.44859 | -3.62546 | -2.68282 | -1.08906 | -3.63734 | -2.66389 | -1.08167 | -2.44221 | -3.81701 | -0.30904 | -2.47061 | -3.80231 | -0.29399 |
| H | -2.36084 | -2.51451 | -2.01446 | -2.00876 | -2.86362 | -1.77991 | -2.01869 | -2.86592 | -1.76155 | -3.68428 | -2.74288 | -0.92867 | -3.69904 | -2.71744 | -0.9227 |
| H | 2.54488 | -0.76375 | 2.89037 | 1.45113 | -0.48307 | 3.54244 | 1.5443 | 1.15888 | 2.88625 | 1.55686 | 1.16837 | 2.90145 | 2.86349 | 0.03972 | 2.61523 |
| H | 1.43659 | 0.57304 | 3.1508 | 2.86306 | 0.02398 | 2.60359 | 1.45131 | -0.47474 | 3.54943 | 1.46618 | -0.4608 | 3.57793 | 1.54916 | 1.1696 | 2.90034 |
| H | 0.99589 | -1.04783 | 3.69769 | 1.55062 | 1.15449 | 2.88746 | 2.85936 | 0.02976 | 2.60383 | 2.86717 | 0.03502 | 2.61701 | 1.46762 | -0.45647 | 3.58293 |
| H | 4.48125 | -2.68779 | -0.53228 | 4.98711 | -1.9604 | -0.00671 | 5.27883 | -0.58437 | -1.02535 | 4.89539 | -2.07991 | -0.08849 | 4.7865 | -2.21697 | -1.86961 |
| H | 4.84593 | -1.96111 | -2.12248 | 4.92187 | -2.11996 | -1.77427 | 4.96624 | -1.99463 | -0.01713 | 4.81641 | -2.17671 | -1.86035 | 5.2231 | -0.68063 | -1.06428 |
| H | 5.31297 | -1.13913 | -0.61295 | 5.29389 | -0.54561 | -1.01043 | 4.89491 | -2.15798 | -1.78428 | 5.24116 | -0.64196 | -1.04524 | 4.875 | -2.11192 | -0.0985 |

## Table S24. Optimized cartesian coordinates of conformers of (1*R,*2*R*,4*R*,5*S,*10*S*)-2 at B3LYP/6-311G(d) level in chloroform.

|  | (1*R,*2*R*,4*R*,5*S,*10*S*)-2-1 | | | (1*R,*2*R*,4*R*,5*S,*10*S*)-2-2 | | | (1*R,*2*R*,4*R*,5*S,*10*S*)-2-3 | | | (1*R,*2*R*,4*R*,5*S,*10*S*)-2-4 | | |
| --- | --- | --- | --- | --- | --- | --- | --- | --- | --- | --- | --- | --- |
| C | 0.4634 | -1.97166 | -0.10904 | 0.47167 | -1.98164 | -0.10922 | 0.45983 | -1.97457 | -0.12639 | 0.4676 | -1.98688 | -0.12313 |
| C | -1.62665 | 0.33652 | 0.01815 | -1.61773 | 0.32361 | 0.02995 | -1.62252 | 0.33688 | 0.02329 | -1.61219 | 0.32339 | 0.03692 |
| C | -1.47241 | 1.86 | 0.03636 | -1.46729 | 1.8482 | 0.05544 | -1.46598 | 1.86038 | 0.04668 | -1.46089 | 1.84813 | 0.06796 |
| C | -0.21937 | 2.36667 | -0.67482 | -0.22375 | 2.37676 | -0.67209 | -0.2192 | 2.36927 | -0.67474 | -0.22552 | 2.38171 | -0.67114 |
| C | 1.1047 | 1.9014 | -0.09255 | 1.10361 | 1.91952 | -0.10392 | 1.10871 | 1.90575 | -0.10271 | 1.10738 | 1.92572 | -0.11757 |
| C | 1.7105 | 0.65039 | -0.69578 | 1.6965 | 0.65614 | -0.70286 | 1.70922 | 0.65253 | -0.71073 | 1.69025 | 0.65717 | -0.72038 |
| C | 2.43486 | -0.37912 | 0.16138 | 2.42437 | -0.36773 | 0.15811 | 2.41613 | -0.37465 | 0.15257 | 2.40314 | -0.36229 | 0.14758 |
| C | 2.01209 | -1.83913 | -0.15947 | 2.01861 | -1.83205 | -0.16454 | 2.00795 | -1.8341 | -0.18785 | 2.01353 | -1.82698 | -0.19147 |
| C | -0.18216 | -1.39991 | 1.11823 | -0.17338 | -1.4163 | 1.12121 | -0.18066 | -1.41214 | 1.10818 | -0.17172 | -1.42933 | 1.11421 |
| C | -1.07334 | -0.40633 | 1.21455 | -1.06295 | -0.42192 | 1.22313 | -1.06666 | -0.41457 | 1.21363 | -1.05525 | -0.4302 | 1.22453 |
| H | -1.21872 | -0.06213 | -0.90559 | -1.20745 | -0.06834 | -0.89556 | -1.21976 | -0.05819 | -0.90416 | -1.20614 | -0.06469 | -0.89195 |
| O | -0.31797 | 3.79604 | -0.63679 | -0.22123 | 3.80973 | -0.63115 | -0.32017 | 3.79905 | -0.63675 | -0.22813 | 3.8151 | -0.62645 |
| C | 1.31779 | 2.20199 | 1.37646 | 1.32882 | 2.22471 | 1.36229 | 1.32995 | 2.20496 | 1.3653 | 1.3465 | 2.23239 | 1.34605 |
| C | 2.30651 | 2.00613 | -1.0033 | 2.29239 | 2.00978 | -1.02194 | 2.30694 | 2.00176 | -1.01943 | 2.28883 | 2.00198 | -1.04662 |
| H | 1.1436 | 0.23615 | -1.52071 | 1.12104 | 0.23547 | -1.51834 | 1.13778 | 0.23743 | -1.53131 | 1.10586 | 0.23376 | -1.52731 |
| O | 3.86062 | -0.22331 | 0.11322 | 3.8493 | -0.20004 | 0.11758 | 3.83403 | -0.17473 | 0.03828 | 3.82008 | -0.14899 | 0.04146 |
| C | 2.49053 | -2.25721 | -1.55705 | 2.49845 | -2.24265 | -1.56362 | 2.48706 | -2.22741 | -1.59056 | 2.49254 | -2.21419 | -1.59571 |
| C | 2.6553 | -2.76684 | 0.8822 | 2.67431 | -2.7541 | 0.87446 | 2.65508 | -2.77853 | 0.83944 | 2.67621 | -2.76301 | 0.83363 |
| C | -1.65032 | -0.01232 | 2.55213 | -1.63608 | -0.03021 | 2.56297 | -1.63606 | -0.02475 | 2.55562 | -1.61984 | -0.04083 | 2.56861 |
| C | -4.97435 | -1.19262 | -0.5599 | -4.95885 | -1.21812 | -0.55896 | -4.97642 | -1.18419 | -0.54268 | -4.95783 | -1.21311 | -0.54376 |
| C | -3.48729 | -1.00308 | -0.70706 | -3.47342 | -1.01785 | -0.70776 | -3.48907 | -0.99939 | -0.69376 | -3.4722 | -1.01573 | -0.69533 |
| O | -3.07368 | 0.08163 | -0.0231 | -3.0645 | 0.06319 | -0.01419 | -3.0706 | 0.08504 | -0.01248 | -3.05988 | 0.06451 | -0.00282 |
| O | -2.75559 | -1.72728 | -1.34061 | -2.73878 | -1.7312 | -1.34978 | -2.76117 | -1.72677 | -1.32798 | -2.74029 | -1.73073 | -1.33864 |
| H | 0.23324 | -3.04366 | -0.14705 | 0.25221 | -3.05589 | -0.14647 | 0.23097 | -3.0463 | -0.17355 | 0.25119 | -3.0613 | -0.16699 |
| H | 0.02329 | -1.55414 | -1.01303 | 0.02375 | -1.56804 | -1.01117 | 0.01523 | -1.54923 | -1.02403 | 0.01327 | -1.56824 | -1.01901 |
| H | -1.50088 | 2.2592 | 1.05131 | -1.48606 | 2.24236 | 1.07252 | -1.48469 | 2.25529 | 1.0636 | -1.46857 | 2.23755 | 1.08704 |
| H | -2.33034 | 2.28762 | -0.48677 | -2.34416 | 2.26315 | -0.4514 | -2.32883 | 2.29048 | -0.46623 | -2.34392 | 2.26489 | -0.4266 |
| H | -0.26097 | 2.03249 | -1.72262 | -0.27636 | 2.04705 | -1.72028 | -0.26883 | 2.03499 | -1.72212 | -0.28809 | 2.05472 | -1.71957 |
| H | 2.19491 | -0.20529 | 1.21121 | 2.17649 | -0.19747 | 1.2068 | 2.13349 | -0.20991 | 1.19769 | 2.11356 | -0.19975 | 1.19118 |
| H | 0.11715 | -1.87217 | 2.05255 | 0.12751 | -1.8921 | 2.05319 | 0.11967 | -1.89274 | 2.03829 | 0.13017 | -1.91272 | 2.04233 |
| H | 0.50253 | 4.15006 | -1.00127 | -1.07299 | 4.12135 | -0.95884 | 0.49903 | 4.15259 | -1.00466 | -1.08 | 4.12427 | -0.95613 |
| H | 2.35922 | 2.0589 | 1.66832 | 2.3667 | 2.05038 | 1.6499 | 2.3731 | 2.05763 | 1.64963 | 0.7204 | 1.62297 | 2.00206 |
| H | 1.05363 | 3.24154 | 1.58245 | 1.10075 | 3.27408 | 1.55871 | 1.07211 | 3.24577 | 1.57334 | 2.38667 | 2.05281 | 1.62271 |
| H | 0.70502 | 1.56208 | 2.01435 | 0.70007 | 1.60942 | 2.00957 | 0.71838 | 1.56834 | 2.00815 | 1.12709 | 3.28359 | 1.5429 |
| H | 3.25497 | 2.25454 | -0.54331 | 3.24478 | 2.25968 | -0.57056 | 3.26393 | 2.23305 | -0.57076 | 3.2529 | 2.23086 | -0.61201 |
| H | 2.17835 | 2.38942 | -2.01098 | 2.14855 | 2.40311 | -2.02219 | 2.17467 | 2.38201 | -2.02782 | 2.13767 | 2.38994 | -2.04807 |
| H | 4.11729 | -0.12622 | -0.81131 | 4.10544 | -0.07247 | -0.80329 | 4.25965 | -0.65075 | 0.7598 | 4.24497 | -0.61316 | 0.77103 |
| H | 3.58165 | -2.26462 | -1.61732 | 2.10685 | -1.5846 | -2.34423 | 2.02811 | -1.61011 | -2.36687 | 3.57272 | -2.08629 | -1.68121 |
| H | 2.11069 | -1.59449 | -2.3396 | 2.16784 | -3.25827 | -1.79724 | 2.2313 | -3.26896 | -1.80471 | 2.02153 | -1.60383 | -2.37024 |
| H | 2.14559 | -3.2679 | -1.79093 | 3.58937 | -2.23312 | -1.62659 | 3.56933 | -2.11592 | -1.67225 | 2.25191 | -3.25968 | -1.80858 |
| H | 2.41175 | -3.81239 | 0.67343 | 2.44055 | -3.80195 | 0.6657 | 2.32371 | -3.80785 | 0.68017 | 3.76548 | -2.75118 | 0.74022 |
| H | 3.74033 | -2.65686 | 0.87021 | 3.75808 | -2.63317 | 0.85926 | 3.745 | -2.77925 | 0.75161 | 2.42457 | -2.48781 | 1.86194 |
| H | 2.31233 | -2.53799 | 1.89473 | 2.33229 | -2.52935 | 1.88827 | 2.40149 | -2.50173 | 1.86678 | 2.35564 | -3.79625 | 0.67728 |
| H | -1.39742 | 1.01627 | 2.82705 | -1.3722 | 0.99402 | 2.84409 | -2.72826 | -0.07427 | 2.54561 | -2.71265 | -0.07591 | 2.55867 |
| H | -2.74204 | -0.07141 | 2.53861 | -2.72837 | -0.07871 | 2.5498 | -1.27149 | -0.68627 | 3.34282 | -1.26398 | -0.71163 | 3.35189 |
| H | -1.2827 | -0.6656 | 3.34469 | -1.27446 | -0.69152 | 3.35157 | -1.37326 | 0.99993 | 2.83593 | -1.34247 | 0.97836 | 2.85518 |
| H | -5.33929 | -1.86064 | -1.33756 | -5.32243 | -1.8766 | -1.34535 | -5.18249 | -1.62799 | 0.43536 | -5.15792 | -1.67461 | 0.4273 |
| H | -5.18092 | -1.64157 | 0.41568 | -5.15924 | -1.68342 | 0.41023 | -5.4972 | -0.22747 | -0.58736 | -5.48589 | -0.25975 | -0.5713 |
| H | -5.4978 | -0.23722 | -0.60115 | -5.48853 | -0.2656 | -0.58375 | -5.34475 | -1.85458 | -1.31668 | -5.32348 | -1.87356 | -1.32754 |

## Table S25. Optimized cartesian coordinates of conformers of (1*S,*2*R*,4*R*,5*R,*10*R*)-2 at B3LYP/6-311G(d) level in chloroform.

|  | (1*S,*2*R*,4*R*,5*R,*10*R*)-2-1 | | | (1*S,*2*R*,4*R*,5*R,*10*R*)-2-2 | | | (1*S,*2*R*,4*R*,5*R,*10*R*)-2-3 | | | (1*S,*2*R*,4*R*,5*R,*10*R*)-2-4 | | | (1*S,*2*R*,4*R*,5*R,*10*R*)-2-5 | | |
| --- | --- | --- | --- | --- | --- | --- | --- | --- | --- | --- | --- | --- | --- | --- | --- |
| C | -0.72549 | -1.77123 | 1.0141 | -0.70425 | -1.84689 | 0.98744 | -0.72919 | -1.83004 | 0.98149 | -0.57468 | -1.81607 | 0.43025 | -1.04356 | -1.94324 | 0.61388 |
| C | 1.40327 | 0.31701 | 0.36865 | 1.3719 | 0.30711 | 0.39621 | 1.3809 | 0.29736 | 0.39343 | 1.53315 | 0.4136 | 0.0377 | 1.35525 | -0.00872 | 0.4126 |
| C | 1.64137 | 1.77229 | -0.0328 | 1.62539 | 1.76489 | 0.01293 | 1.6462 | 1.753 | 0.01146 | 1.45798 | 1.93188 | -0.1128 | 1.65635 | 1.49115 | 0.2721 |
| C | 0.3429 | 2.58647 | -0.20231 | 0.35571 | 2.59975 | -0.2036 | 0.37207 | 2.59342 | -0.20947 | 0.06378 | 2.55037 | 0.08744 | 0.43496 | 2.42829 | 0.26817 |
| C | -0.68242 | 1.8591 | -1.08215 | -0.6804 | 1.87487 | -1.0714 | -0.65419 | 1.87186 | -1.07843 | -1.04079 | 1.87946 | -0.74224 | -0.63057 | 1.99389 | -0.74345 |
| C | -1.63183 | 0.91696 | -0.36168 | -1.5855 | 0.90381 | -0.33703 | -1.58963 | 0.92773 | -0.34125 | -1.94457 | 0.88901 | -0.02227 | -1.6586 | 1.00775 | -0.23358 |
| C | -2.06841 | -0.48312 | -0.79329 | -2.01592 | -0.49109 | -0.79629 | -2.04072 | -0.46337 | -0.79204 | -2.51733 | -0.42156 | -0.52915 | -2.16228 | -0.24509 | -0.94356 |
| C | -2.13148 | -1.48423 | 0.4051 | -2.10092 | -1.51947 | 0.37618 | -2.12799 | -1.48881 | 0.38308 | -2.11746 | -1.65383 | 0.33975 | -2.3973 | -1.42561 | 0.03757 |
| C | -0.10232 | -0.79729 | 1.98721 | -0.09331 | -0.8924 | 1.98586 | -0.1035 | -0.88584 | 1.97968 | 0.15827 | -1.06208 | 1.50569 | -0.37634 | -1.20723 | 1.74937 |
| C | 0.89684 | 0.07034 | 1.76941 | 0.86008 | 0.02572 | 1.78701 | 0.86363 | 0.01795 | 1.78236 | 1.1202 | -0.13725 | 1.38155 | 0.71566 | -0.42912 | 1.71592 |
| H | 0.70832 | -0.11031 | -0.33546 | 0.69034 | -0.1121 | -0.32449 | 0.69575 | -0.11407 | -0.32853 | 0.95863 | -0.05958 | -0.74998 | 0.75807 | -0.33659 | -0.43284 |
| O | -0.19089 | 3.00524 | 1.05754 | -0.14588 | 2.95823 | 1.08734 | -0.24094 | 2.9561 | 1.0329 | -0.26849 | 2.66345 | 1.47488 | -0.09395 | 2.63041 | 1.58211 |
| C | -0.16752 | 1.58473 | -2.4843 | -0.19237 | 1.63956 | -2.4893 | -0.14613 | 1.60477 | -2.48392 | -0.68127 | 1.77854 | -2.21417 | -0.10326 | 1.96805 | -2.16772 |
| C | -2.13926 | 2.21295 | -0.94917 | -2.14566 | 2.18971 | -0.89714 | -2.11147 | 2.22 | -0.92615 | -2.46647 | 2.24791 | -0.41539 | -2.05831 | 2.43219 | -0.54184 |
| H | -1.53421 | 0.984 | 0.71078 | -1.42703 | 0.97593 | 0.72821 | -1.44838 | 1.00419 | 0.72549 | -1.73956 | 0.83392 | 1.03634 | -1.56678 | 0.84828 | 0.82823 |
| O | -1.32735 | -1.02411 | -1.88975 | -1.26318 | -1.01211 | -1.89621 | -1.30366 | -0.99963 | -1.89599 | -2.15215 | -0.60579 | -1.89869 | -1.25727 | -0.74657 | -1.93836 |
| C | -3.10407 | -0.97307 | 1.47721 | -3.06845 | -1.01105 | 1.45393 | -3.0812 | -0.97018 | 1.46866 | -2.77071 | -1.54328 | 1.72499 | -3.3725 | -1.02248 | 1.15112 |
| C | -2.67255 | -2.81772 | -0.13889 | -2.66524 | -2.82857 | -0.20229 | -2.71088 | -2.79288 | -0.18863 | -2.67682 | -2.91454 | -0.34458 | -3.02877 | -2.58064 | -0.75967 |
| C | 1.58733 | 0.79489 | 2.89645 | 1.46285 | 0.81148 | 2.91839 | 1.48255 | 0.78141 | 2.92059 | 1.89488 | 0.36392 | 2.57699 | 1.40419 | 0.01424 | 2.98425 |
| C | 4.03326 | -2.01658 | -0.84408 | 4.03313 | -1.99529 | -0.82327 | 4.01645 | -2.03685 | -0.82177 | 4.69756 | -1.31317 | -0.90215 | 4.41909 | -1.75428 | -0.78789 |
| C | 2.69268 | -1.33964 | -0.78049 | 2.69333 | -1.31425 | -0.77487 | 2.68398 | -1.34132 | -0.77412 | 3.2114 | -1.07741 | -0.82863 | 3.12919 | -0.98158 | -0.88134 |
| O | 2.66469 | -0.40978 | 0.18953 | 2.64295 | -0.41258 | 0.21681 | 2.64455 | -0.43594 | 0.2155 | 2.94305 | 0.07971 | -0.19201 | 2.63529 | -0.72683 | 0.3439 |
| O | 1.75374 | -1.60078 | -1.50589 | 1.77217 | -1.55775 | -1.53012 | 1.75954 | -1.57659 | -1.52746 | 2.36863 | -1.82426 | -1.26723 | 2.60133 | -0.62994 | -1.91177 |
| H | -0.03396 | -1.99792 | 0.20133 | -0.00912 | -2.05848 | 0.17329 | -0.04185 | -2.04296 | 0.16119 | -0.15185 | -1.6303 | -0.55542 | -0.35796 | -2.06595 | -0.22395 |
| H | -0.81975 | -2.71165 | 1.56857 | -0.81537 | -2.80178 | 1.51302 | -0.84304 | -2.78633 | 1.50409 | -0.37716 | -2.87609 | 0.63602 | -1.23666 | -2.9532 | 0.99363 |
| H | 2.17236 | 1.76007 | -0.98753 | 2.18733 | 1.75351 | -0.92376 | 2.21535 | 1.74093 | -0.92183 | 1.81402 | 2.17266 | -1.11758 | 2.19202 | 1.62086 | -0.66981 |
| H | 2.29381 | 2.28151 | 0.67918 | 2.25663 | 2.26566 | 0.74912 | 2.28688 | 2.24351 | 0.74957 | 2.14796 | 2.41435 | 0.58334 | 2.33696 | 1.80971 | 1.0649 |
| H | 0.60558 | 3.52619 | -0.6972 | 0.65458 | 3.51741 | -0.72972 | 0.67069 | 3.51168 | -0.73416 | 0.12885 | 3.59035 | -0.24844 | 0.78978 | 3.42207 | -0.02297 |
| H | -3.08677 | -0.42027 | -1.18877 | -3.02868 | -0.41813 | -1.20445 | -3.05597 | -0.37984 | -1.19213 | -3.61414 | -0.35292 | -0.46928 | -3.12462 | -0.02432 | -1.4253 |
| H | -0.452 | -0.8955 | 3.01356 | -0.44438 | -1.02098 | 3.00842 | -0.45403 | -1.01242 | 3.00267 | -0.0754 | -1.38331 | 2.51991 | -0.78993 | -1.42126 | 2.73401 |
| H | -0.35301 | 2.22619 | 1.60319 | -0.82101 | 3.63575 | 0.9713 | 0.31916 | 3.61022 | 1.46354 | -0.23615 | 1.78084 | 1.86431 | -0.29872 | 1.77094 | 1.96973 |
| H | 0.15577 | 2.5268 | -2.94085 | 0.68117 | 0.9851 | -2.52885 | 0.7186 | 0.93746 | -2.50341 | -1.54184 | 1.49613 | -2.81296 | 0.37294 | 2.92689 | -2.39802 |
| H | 0.68358 | 0.90085 | -2.50087 | -0.96145 | 1.17913 | -3.1048 | -0.91432 | 1.14621 | -3.10146 | -0.32055 | 2.7532 | -2.56285 | 0.63693 | 1.18388 | -2.33317 |
| H | -0.9395 | 1.14623 | -3.11209 | 0.08886 | 2.59674 | -2.94254 | 0.15446 | 2.55046 | -2.94867 | 0.09889 | 1.04157 | -2.41234 | -0.90973 | 1.84941 | -2.89645 |
| H | -2.73097 | 2.15611 | -1.85734 | -2.75248 | 2.12675 | -1.79486 | -2.71149 | 2.154 | -1.82878 | -3.15194 | 2.30109 | -1.25522 | -2.63772 | 2.61361 | -1.44214 |
| H | -2.42942 | 2.99472 | -0.25732 | -2.47917 | 2.94912 | -0.19681 | -2.39905 | 3.00408 | -0.23674 | -2.65404 | 2.95932 | 0.38078 | -2.29843 | 3.07585 | 0.29575 |
| H | -0.38813 | -1.10568 | -1.67142 | -0.32577 | -1.10129 | -1.67143 | -0.36427 | -1.08616 | -1.68015 | -2.72516 | -1.28437 | -2.27096 | -1.17792 | -0.09931 | -2.64412 |
| H | -3.22152 | -1.71904 | 2.26844 | -2.718 | -0.10164 | 1.94392 | -3.21823 | -1.7272 | 2.24645 | -2.45832 | -0.65404 | 2.27493 | -2.9862 | -0.23228 | 1.79642 |
| H | -4.09272 | -0.78999 | 1.0464 | -3.20412 | -1.7695 | 2.23047 | -4.06569 | -0.75007 | 1.04506 | -2.51686 | -2.41179 | 2.33924 | -3.59208 | -1.88418 | 1.78755 |
| H | -2.77302 | -0.04744 | 1.9507 | -4.05171 | -0.80054 | 1.02255 | -2.71846 | -0.06394 | 1.95535 | -3.86041 | -1.51028 | 1.63543 | -4.31959 | -0.6727 | 0.72974 |
| H | -2.80027 | -3.54065 | 0.67139 | -2.80838 | -3.56899 | 0.5896 | -2.85652 | -3.53008 | 0.60583 | -2.56499 | -3.78451 | 0.30695 | -3.26051 | -3.4212 | -0.09993 |
| H | -3.6458 | -2.67487 | -0.61741 | -3.63468 | -2.65702 | -0.67968 | -3.68181 | -2.61138 | -0.65923 | -3.74382 | -2.80753 | -0.56825 | -3.96223 | -2.26058 | -1.2325 |
| H | -1.99831 | -3.24594 | -0.8808 | -1.99632 | -3.25099 | -0.95261 | -2.05221 | -3.22396 | -0.943 | -2.14894 | -3.13939 | -1.27429 | -2.35798 | -2.9319 | -1.54347 |
| H | 2.65576 | 0.55457 | 2.89875 | 1.10361 | 0.45084 | 3.88387 | 1.24044 | 1.84512 | 2.86242 | 1.83295 | 1.44956 | 2.69253 | 0.8811 | -0.35726 | 3.86682 |
| H | 1.51481 | 1.88218 | 2.80895 | 2.55526 | 0.72776 | 2.91236 | 1.13117 | 0.40823 | 3.88384 | 1.53705 | -0.09623 | 3.49946 | 2.42656 | -0.37568 | 3.00787 |
| H | 1.17515 | 0.50374 | 3.86367 | 1.21625 | 1.87204 | 2.83302 | 2.5743 | 0.69527 | 2.90253 | 2.9568 | 0.125 | 2.46781 | 1.48567 | 1.10087 | 3.06926 |
| H | 4.22058 | -2.54666 | 0.0925 | 4.16908 | -2.59273 | 0.08145 | 4.82589 | -1.30543 | -0.84608 | 5.22768 | -0.39211 | -1.14596 | 5.16703 | -1.165 | -0.25255 |
| H | 4.05395 | -2.71918 | -1.67378 | 4.0896 | -2.64019 | -1.69714 | 4.14505 | -2.63646 | 0.0826 | 5.05499 | -1.65134 | 0.0744 | 4.26516 | -2.67577 | -0.22301 |
| H | 4.82436 | -1.27383 | -0.96191 | 4.8344 | -1.25496 | -0.84851 | 4.06632 | -2.6819 | -1.69592 | 4.91228 | -2.08216 | -1.64142 | 4.78252 | -1.98719 | -1.78631 |

## Table S26. Optimized cartesian coordinates of conformers of (1*S,*2*R*,4*R*,5*R,*10*R*)-2 at B3LYP/6-311G(d) level in chloroform.

|  | (1*S,*2*R*,4*R*,5*R,*10*R*)-2-6 | | | (1*S,*2*R*,4*R*,5*R,*10*R*)-2-7 | | | (1*S,*2*R*,4*R*,5*R,*10*R*)-2-8 | | | (1*S,*2*R*,4*R*,5*R,*10*R*)-2-9 | | |
| --- | --- | --- | --- | --- | --- | --- | --- | --- | --- | --- | --- | --- |
| C | -0.51359 | -1.93895 | 0.58688 | -1.04525 | -2.00242 | 0.61579 | 0.2884 | -1.75439 | 0.20735 | -0.4986 | -1.9372 | 0.66087 |
| C | 1.43328 | 0.41036 | 0.10185 | 1.31827 | -0.0239 | 0.42696 | -1.81323 | 0.69716 | 0.68882 | 1.40436 | 0.44384 | 0.06766 |
| C | 1.40527 | 1.92592 | -0.10675 | 1.65184 | 1.46886 | 0.28123 | -1.48331 | 2.12311 | 0.2168 | 1.31547 | 1.95865 | -0.15913 |
| C | 0.03845 | 2.61104 | -0.01419 | 0.47272 | 2.44669 | 0.21753 | -0.40041 | 2.34661 | -0.85483 | -0.06799 | 2.60737 | 0.02544 |
| C | -1.05549 | 1.90112 | -0.81827 | -0.6124 | 2.00094 | -0.76701 | 1.02605 | 1.94815 | -0.45945 | -1.16479 | 1.91284 | -0.78697 |
| C | -1.82542 | 0.82003 | -0.08395 | -1.59851 | 0.99281 | -0.22434 | 1.54919 | 0.61478 | -0.96562 | -1.86952 | 0.76399 | -0.10114 |
| C | -2.2906 | -0.52255 | -0.62579 | -2.10254 | -0.25901 | -0.9377 | 2.42681 | -0.38521 | -0.21616 | -2.20323 | -0.59674 | -0.70404 |
| C | -2.03308 | -1.71491 | 0.34454 | -2.37799 | -1.43886 | 0.03226 | 1.79052 | -1.80847 | -0.18517 | -2.00645 | -1.75449 | 0.31063 |
| C | 0.16527 | -1.09578 | 1.63149 | -0.38179 | -1.27105 | 1.75526 | -0.02235 | -0.9866 | 1.45992 | 0.1568 | -1.02346 | 1.66597 |
| C | 1.03711 | -0.09438 | 1.46826 | 0.67196 | -0.44623 | 1.725 | -0.85739 | 0.03933 | 1.66832 | 1.04416 | -0.03909 | 1.45621 |
| H | 0.85201 | -0.08082 | -0.67098 | 0.73187 | -0.35088 | -0.42594 | -2.76715 | 0.76049 | 1.21758 | 0.81859 | -0.08245 | -0.68019 |
| O | -0.28383 | 2.75084 | 1.37373 | -0.0241 | 2.62332 | 1.54726 | -0.80045 | 1.79789 | -2.11213 | -0.40942 | 2.75618 | 1.40631 |
| C | -0.73824 | 1.85687 | -2.30118 | -0.11736 | 1.98873 | -2.20231 | 1.46416 | 2.593 | 0.84345 | -0.88669 | 1.93346 | -2.27943 |
| C | -2.49676 | 2.12813 | -0.43036 | -2.05005 | 2.40137 | -0.53128 | 2.05176 | 1.90294 | -1.56541 | -2.60193 | 2.06097 | -0.35497 |
| H | -1.53043 | 0.7833 | 0.9535 | -1.44034 | 0.85236 | 0.83309 | 0.83601 | 0.13251 | -1.62039 | -1.57893 | 0.67563 | 0.93287 |
| O | -1.67985 | -0.75586 | -1.8997 | -1.18393 | -0.77205 | -1.91616 | 2.80666 | 0.06007 | 1.08821 | -1.39503 | -0.92916 | -1.843 |
| C | -2.80224 | -1.49453 | 1.65412 | -3.34459 | -1.00803 | 1.14246 | 1.88574 | -2.42583 | -1.5902 | -2.86031 | -1.52494 | 1.56477 |
| C | -2.58393 | -2.99081 | -0.32018 | -3.04121 | -2.56675 | -0.77758 | 2.57654 | -2.70035 | 0.78565 | -2.48133 | -3.05458 | -0.36207 |
| C | 1.69873 | 0.57533 | 2.64378 | 1.28767 | 0.08904 | 2.99046 | -1.02295 | 0.60384 | 3.06114 | 1.79211 | 0.60107 | 2.60231 |
| C | 4.59929 | -1.3403 | -0.82531 | 4.38701 | -1.8 | -0.7339 | -3.12326 | -1.82487 | -1.71058 | 4.61831 | -1.21229 | -0.85539 |
| C | 3.11508 | -1.07751 | -0.7958 | 3.11152 | -1.00438 | -0.84501 | -2.99533 | -1.08949 | -0.40627 | 3.12461 | -1.02777 | -0.777 |
| O | 2.84638 | 0.0505 | -0.1156 | 2.59809 | -0.75455 | 0.37052 | -2.07455 | -0.09336 | -0.50582 | 2.82161 | 0.13646 | -0.17214 |
| O | 2.27966 | -1.78924 | -1.30366 | 2.61295 | -0.6355 | -1.88497 | -3.59901 | -1.32869 | 0.60717 | 2.30803 | -1.81763 | -1.18842 |
| H | -0.00251 | -1.8695 | -0.37223 | -0.35512 | -2.13879 | -0.21659 | -0.04748 | -2.79066 | 0.34643 | 0.06814 | -1.95079 | -0.26758 |
| H | -0.39511 | -2.98053 | 0.91088 | -1.2705 | -3.00885 | 0.98659 | -0.27419 | -1.36155 | -0.63165 | -0.40359 | -2.94502 | 1.08283 |
| H | 1.81566 | 2.11183 | -1.10215 | 2.2201 | 1.57282 | -0.64434 | -1.24697 | 2.72235 | 1.09809 | 1.65777 | 2.14455 | -1.18043 |
| H | 2.07908 | 2.41369 | 0.60071 | 2.31259 | 1.7818 | 1.09234 | -2.40847 | 2.54112 | -0.19059 | 2.0228 | 2.4712 | 0.49685 |
| H | 0.15884 | 3.61667 | -0.44268 | 0.87299 | 3.40894 | -0.13317 | -0.37408 | 3.43063 | -1.02082 | 0.01211 | 3.63852 | -0.33354 |
| H | -3.38043 | -0.47661 | -0.76929 | -3.05045 | -0.02472 | -1.44135 | 3.38247 | -0.47865 | -0.74161 | -3.25827 | -0.60714 | -1.00983 |
| H | -0.06124 | -1.3778 | 2.65921 | -0.79524 | -1.49348 | 2.73815 | 0.49567 | -1.35224 | 2.34558 | -0.05469 | -1.26612 | 2.70693 |
| H | -1.01647 | 3.37138 | 1.45206 | -0.60835 | 3.38934 | 1.55074 | -1.24055 | 0.9572 | -1.93389 | -0.37129 | 1.88992 | 1.82965 |
| H | -0.50487 | 2.86908 | -2.65183 | -0.93264 | 1.83423 | -2.91427 | 2.5315 | 2.47456 | 1.01142 | -1.76276 | 1.63573 | -2.86066 |
| H | 0.11608 | 1.21566 | -2.52391 | 0.32184 | 2.96076 | -2.4501 | 1.24337 | 3.66569 | 0.80784 | -0.64494 | 2.95454 | -2.59239 |
| H | -1.5769 | 1.47618 | -2.87772 | 0.64719 | 1.2288 | -2.37332 | 0.94558 | 2.18595 | 1.71456 | -0.05225 | 1.29241 | -2.56737 |
| H | -3.21223 | 2.12466 | -1.24665 | -2.65074 | 2.56006 | -1.42179 | 3.05518 | 2.22294 | -1.30282 | -3.34349 | 2.0804 | -1.14793 |
| H | -2.76455 | 2.79902 | 0.38044 | -2.32816 | 3.04092 | 0.30008 | 1.74097 | 2.13671 | -2.57716 | -2.83231 | 2.68078 | 0.50305 |
| H | -2.12275 | -1.50603 | -2.31085 | -1.09434 | -0.13148 | -2.6266 | 1.99436 | 0.24435 | 1.57594 | -1.66075 | -0.37543 | -2.58207 |
| H | -2.66842 | -2.34884 | 2.3239 | -3.59694 | -1.86423 | 1.77446 | 1.42346 | -3.41691 | -1.60533 | -3.91714 | -1.4186 | 1.30218 |
| H | -3.87463 | -1.39055 | 1.46193 | -4.27735 | -0.62382 | 0.71835 | 2.92944 | -2.5411 | -1.89576 | -2.56885 | -0.63669 | 2.12728 |
| H | -2.47311 | -0.60433 | 2.1917 | -2.93003 | -0.23483 | 1.79036 | 1.38323 | -1.81682 | -2.34577 | -2.77191 | -2.38073 | 2.23983 |
| H | -1.98219 | -3.29701 | -1.18022 | -3.29759 | -3.40739 | -0.12701 | 2.51617 | -2.3468 | 1.81421 | -1.87667 | -3.2877 | -1.23807 |
| H | -2.57141 | -3.82485 | 0.38561 | -3.96471 | -2.21648 | -1.24911 | 2.19525 | -3.72504 | 0.75062 | -2.41634 | -3.89361 | 0.336 |
| H | -3.61871 | -2.85519 | -0.65212 | -2.3784 | -2.92905 | -1.56345 | 3.63621 | -2.72665 | 0.51593 | -3.52399 | -2.96777 | -0.6832 |
| H | 1.46526 | 0.05556 | 3.57494 | 1.16153 | 1.17111 | 3.06164 | -0.91251 | 1.69229 | 3.08508 | 1.47789 | 0.18224 | 3.5596 |
| H | 2.78688 | 0.58594 | 2.52492 | 0.83512 | -0.36893 | 3.87199 | -2.02333 | 0.38341 | 3.44941 | 2.86669 | 0.42601 | 2.49301 |
| H | 1.3684 | 1.61196 | 2.73728 | 2.36239 | -0.11961 | 3.01539 | -0.29176 | 0.18133 | 3.75101 | 1.65953 | 1.68522 | 2.64524 |
| H | 5.15498 | -0.42164 | -1.01596 | 4.20156 | -2.73233 | -0.19678 | -3.94924 | -2.53004 | -1.655 | 4.99651 | -1.49427 | 0.13107 |
| H | 4.91419 | -1.71963 | 0.15074 | 4.7752 | -2.01553 | -1.72697 | -3.28233 | -1.12568 | -2.53326 | 4.85481 | -2.00372 | -1.56364 |
| H | 4.82703 | -2.08599 | -1.58438 | 5.12865 | -1.23724 | -0.16259 | -2.19777 | -2.36901 | -1.91565 | 5.11184 | -0.28372 | -1.14382 |

## Table S27. Optimized cartesian coordinates of conformers of (1*S,*2*R*,4*R*,5*R,*10*S*)-2 at B3LYP/6-311G(d) level in chloroform.

|  | (1*S,*2*R*,4*R*,5*R,*10*S*)-2-1 | | | (1*S,*2*R*,4*R*,5*R,*10*S*)-2-2 | | | (1*S,*2*R*,4*R*,5*R,*10*S*)-2-3 | | | (1*S,*2*R*,4*R*,5*R,*10*S*)-2-4 | | |
| --- | --- | --- | --- | --- | --- | --- | --- | --- | --- | --- | --- | --- |
| C | 1.03347 | -1.87618 | -0.55135 | 0.48821 | -1.88176 | -0.24086 | 0.47859 | -1.89806 | -0.25128 | 0.99728 | -1.85378 | -0.42879 |
| C | -1.42138 | -0.24829 | 0.38429 | -1.52839 | 0.44064 | 0.11866 | -1.52245 | 0.43976 | 0.11712 | -1.45991 | -0.21985 | 0.40939 |
| C | -1.60569 | 1.22177 | 0.81021 | -1.35133 | 1.9591 | 0.18686 | -1.34032 | 1.95842 | 0.20537 | -1.65242 | 1.27022 | 0.74294 |
| C | -0.76747 | 2.21354 | -0.01216 | -0.12439 | 2.48485 | -0.5777 | -0.12582 | 2.49635 | -0.5561 | -0.76128 | 2.19427 | -0.09731 |
| C | 0.73734 | 2.09001 | 0.23474 | 1.22214 | 2.00287 | -0.04835 | 1.2332 | 2.00732 | -0.04287 | 0.73452 | 2.03724 | 0.19209 |
| C | 1.49195 | 1.07718 | -0.6115 | 1.81434 | 0.75453 | -0.68776 | 1.79973 | 0.74599 | -0.67417 | 1.51834 | 1.07265 | -0.68006 |
| C | 2.60239 | 0.13794 | -0.14089 | 2.57407 | -0.37336 | 0.01131 | 2.55753 | -0.38496 | 0.02244 | 2.64738 | 0.14303 | -0.26014 |
| C | 2.46143 | -1.28765 | -0.75512 | 2.0343 | -1.78279 | -0.37861 | 2.02492 | -1.79225 | -0.3833 | 2.42943 | -1.33064 | -0.72053 |
| C | 0.48234 | -1.80031 | 0.84395 | -0.08107 | -1.38354 | 1.05439 | -0.09343 | -1.40526 | 1.0447 | 0.48011 | -1.67427 | 0.96759 |
| C | -0.57595 | -1.10783 | 1.29116 | -0.93591 | -0.37012 | 1.25144 | -0.9379 | -0.3839 | 1.24531 | -0.60465 | -1.00177 | 1.36908 |
| H | -1.03681 | -0.27517 | -0.62806 | -1.17096 | 0.09319 | -0.84344 | -1.16869 | 0.0957 | -0.84716 | -1.06668 | -0.30186 | -0.59649 |
| O | -1.05239 | 2.09332 | -1.40434 | -0.22447 | 2.14377 | -1.96395 | -0.35482 | 2.17774 | -1.93252 | -1.02921 | 2.03133 | -1.49053 |
| C | 1.12207 | 2.42972 | 1.66312 | 1.43579 | 2.39092 | 1.40365 | 1.47599 | 2.41106 | 1.40021 | 1.06206 | 2.29513 | 1.65053 |
| C | 1.67507 | 2.55774 | -0.84759 | 2.41325 | 2.1121 | -0.96647 | 2.41718 | 2.08967 | -0.97581 | 1.69581 | 2.56422 | -0.84282 |
| H | 0.85107 | 0.64648 | -1.36807 | 1.20812 | 0.40973 | -1.51361 | 1.17252 | 0.40556 | -1.48707 | 0.90531 | 0.66382 | -1.47207 |
| O | 2.74019 | 0.0803 | 1.28294 | 2.65883 | -0.21119 | 1.43014 | 2.62855 | -0.23099 | 1.44252 | 2.86263 | 0.23579 | 1.1515 |
| C | 2.73518 | -1.21097 | -2.26518 | 2.40069 | -2.06999 | -1.84354 | 2.39827 | -2.06292 | -1.84949 | 2.65835 | -1.40542 | -2.23918 |
| C | 3.50757 | -2.21361 | -0.1177 | 2.71398 | -2.84051 | 0.50366 | 2.70564 | -2.85485 | 0.49215 | 3.4667 | -2.23321 | -0.03382 |
| C | -1.0234 | -1.20541 | 2.72723 | -1.44849 | -0.04376 | 2.6323 | -1.44674 | -0.05925 | 2.62816 | -1.04016 | -1.00544 | 2.81191 |
| C | -4.81014 | -1.37627 | -0.66954 | -4.92435 | -1.01828 | -0.36047 | -4.92985 | -0.98383 | -0.38204 | -4.83688 | -1.39326 | -0.63235 |
| C | -3.51185 | -0.62036 | -0.71353 | -3.44167 | -0.84721 | -0.56569 | -3.44226 | -0.83517 | -0.56928 | -3.52802 | -0.65626 | -0.69835 |
| O | -2.73449 | -0.91265 | 0.335 | -2.98048 | 0.20635 | 0.1379 | -2.97571 | 0.21554 | 0.13417 | -2.77393 | -0.88807 | 0.38027 |
| O | -3.20289 | 0.16964 | -1.58543 | -2.75054 | -1.55976 | -1.25449 | -2.75266 | -1.56122 | -1.24556 | -3.19633 | 0.07504 | -1.61318 |
| H | 1.08204 | -2.93427 | -0.83615 | 0.22338 | -2.94155 | -0.34171 | 0.21784 | -2.95824 | -0.35671 | 1.00665 | -2.93012 | -0.64632 |
| H | 0.35026 | -1.41587 | -1.26507 | 0.01208 | -1.38631 | -1.08456 | 0.00191 | -1.40126 | -1.09394 | 0.31155 | -1.42233 | -1.15777 |
| H | -1.39659 | 1.35496 | 1.87104 | -1.33881 | 2.3224 | 1.21588 | -1.31519 | 2.30194 | 1.23997 | -1.48857 | 1.46292 | 1.80234 |
| H | -2.65647 | 1.49156 | 0.68212 | -2.23804 | 2.4049 | -0.27474 | -2.22056 | 2.41383 | -0.25241 | -2.69118 | 1.54637 | 0.54695 |
| H | -1.06033 | 3.22264 | 0.3134 | -0.13108 | 3.58033 | -0.4802 | -0.13574 | 3.58963 | -0.43941 | -1.02251 | 3.22654 | 0.17858 |
| H | 3.56873 | 0.52785 | -0.47457 | 3.61985 | -0.34174 | -0.31021 | 3.60581 | -0.34761 | -0.28961 | 3.56666 | 0.4787 | -0.7625 |
| H | 1.0118 | -2.40631 | 1.57729 | 0.2323 | -1.93654 | 1.93918 | 0.21204 | -1.9667 | 1.92693 | 1.05061 | -2.18742 | 1.73862 |
| H | -1.83814 | 1.53739 | -1.52843 | -1.00701 | 2.5752 | -2.32425 | 0.24605 | 2.70063 | -2.47387 | -1.80003 | 1.45339 | -1.60562 |
| H | 0.73284 | 3.42194 | 1.91662 | 0.76204 | 1.86669 | 2.0857 | 0.80154 | 1.90629 | 2.09595 | 2.1286 | 2.43577 | 1.801 |
| H | 0.71526 | 1.72546 | 2.39394 | 2.45237 | 2.18269 | 1.72591 | 2.49247 | 2.18915 | 1.71429 | 0.54248 | 3.20106 | 1.98494 |
| H | 2.20078 | 2.43987 | 1.79649 | 1.24973 | 3.46369 | 1.52494 | 1.30837 | 3.48814 | 1.50978 | 0.75869 | 1.46746 | 2.29205 |
| H | 2.60522 | 3.00724 | -0.51395 | 3.36129 | 2.34508 | -0.4915 | 3.37245 | 2.31602 | -0.5134 | 2.6142 | 3.00315 | -0.4649 |
| H | 1.26164 | 2.97177 | -1.75897 | 2.27804 | 2.52905 | -1.957 | 2.31254 | 2.49065 | -1.97937 | 1.30363 | 3.01984 | -1.74449 |
| H | 1.87197 | -0.13536 | 1.64576 | 1.75563 | -0.13728 | 1.76273 | 1.72172 | -0.16257 | 1.76696 | 3.73326 | -0.13096 | 1.33797 |
| H | 2.045 | -0.53665 | -2.77746 | 2.02306 | -3.05038 | -2.14701 | 2.02529 | -3.04126 | -2.16477 | 2.48556 | -2.42135 | -2.605 |
| H | 2.63488 | -2.19831 | -2.72469 | 3.48603 | -2.07412 | -1.98023 | 3.48413 | -2.06164 | -1.9822 | 3.68537 | -1.12964 | -2.49518 |
| H | 3.75198 | -0.85678 | -2.45736 | 1.98015 | -1.33058 | -2.52908 | 1.97712 | -1.31795 | -2.52874 | 1.9883 | -0.73913 | -2.78784 |
| H | 4.51429 | -1.80627 | -0.24841 | 2.45712 | -2.72496 | 1.55608 | 2.44393 | -2.74931 | 1.54442 | 3.30253 | -2.30807 | 1.04235 |
| H | 3.3459 | -2.33816 | 0.95229 | 2.41996 | -3.84459 | 0.18462 | 2.41685 | -3.85718 | 0.16323 | 3.42243 | -3.24517 | -0.44441 |
| H | 3.4811 | -3.19962 | -0.59048 | 3.80288 | -2.76908 | 0.42645 | 3.79456 | -2.77846 | 0.41984 | 4.48433 | -1.86173 | -0.19701 |
| H | -0.95383 | -0.24606 | 3.24936 | -1.17898 | 0.96942 | 2.94652 | -1.14777 | 0.94137 | 2.9558 | -0.97476 | -0.01303 | 3.26904 |
| H | -2.06934 | -1.51912 | 2.78437 | -2.53989 | -0.09888 | 2.66464 | -2.53932 | -0.08173 | 2.65567 | -2.08199 | -1.32679 | 2.90359 |
| H | -0.42019 | -1.92739 | 3.2793 | -1.04926 | -0.73697 | 3.37414 | -1.07139 | -0.7729 | 3.36301 | -0.42277 | -1.68132 | 3.40547 |
| H | -5.40606 | -1.02743 | 0.17751 | -5.09686 | -1.49694 | 0.60759 | -5.33319 | -1.64178 | -1.14915 | -4.6746 | -2.43877 | -0.36694 |
| H | -5.363 | -1.2141 | -1.59194 | -5.43324 | -0.05419 | -0.34725 | -5.12499 | -1.42368 | 0.59993 | -5.46255 | -0.95019 | 0.14677 |
| H | -4.62493 | -2.44135 | -0.5222 | -5.33364 | -1.6537 | -1.14336 | -5.42675 | -0.01378 | -0.41348 | -5.34986 | -1.321 | -1.58875 |

## Table S28. Optimized cartesian coordinates of conformers of (1*S,*2*R,*4*R,*5*S,*10*R*)-2 at B3LYP/6-311G(d) level in chloroform.

|  | (1*S,*2*R,*4*R,*5*S,*10*R*)-2-1 | | | (1*S,*2*R,*4*R,*5*S,*10*R*)-2-2 | | | (1*S,*2*R,*4*R,*5*S,*10*R*)-2-3 | | | (1*S,*2*R,*4*R,*5*S,*10*R*)-2-4 | | |
| --- | --- | --- | --- | --- | --- | --- | --- | --- | --- | --- | --- | --- |
| C | -0.76721 | -1.89942 | 0.38144 | -0.74077 | -1.96847 | 0.5036 | -0.7412 | -1.94761 | 0.45279 | 0.34567 | -1.7473 | 0.29175 |
| C | 1.59081 | 0.06566 | 0.27678 | 1.53082 | 0.07077 | 0.30422 | 1.55154 | 0.08019 | 0.29341 | -1.97541 | 0.54923 | 0.6345 |
| C | 1.739 | 1.58271 | 0.13723 | 1.74114 | 1.57453 | 0.11497 | 1.74084 | 1.58691 | 0.11202 | -1.71193 | 2.02279 | 0.27576 |
| C | 0.43406 | 2.39176 | 0.22575 | 0.47948 | 2.44294 | 0.11678 | 0.45761 | 2.43461 | 0.1181 | -0.57835 | 2.38375 | -0.70077 |
| C | -0.68519 | 1.86678 | -0.68016 | -0.65504 | 1.88013 | -0.74291 | -0.66199 | 1.8643 | -0.74414 | 0.83778 | 2.01761 | -0.25349 |
| C | -1.73745 | 0.96162 | -0.06311 | -1.63826 | 0.93833 | -0.07737 | -1.67014 | 0.94847 | -0.07338 | 1.44924 | 0.73389 | -0.77091 |
| C | -2.33611 | -0.23927 | -0.77184 | -2.20463 | -0.29591 | -0.76623 | -2.25926 | -0.27746 | -0.75521 | 2.3155 | -0.15917 | 0.10118 |
| C | -2.25035 | -1.55468 | 0.05745 | -2.19872 | -1.57555 | 0.12102 | -2.21283 | -1.57116 | 0.10992 | 1.87662 | -1.64848 | 0.04716 |
| C | -0.09374 | -1.23739 | 1.554 | -0.07649 | -1.25147 | 1.64798 | -0.0731 | -1.25251 | 1.60758 | -0.14098 | -1.03058 | 1.51781 |
| C | 0.97977 | -0.43437 | 1.56026 | 0.943 | -0.38606 | 1.61287 | 0.95206 | -0.39359 | 1.58999 | -1.06748 | -0.07924 | 1.67433 |
| H | 1.03272 | -0.30781 | -0.57243 | 0.94612 | -0.30666 | -0.5253 | 0.98194 | -0.30012 | -0.54473 | -2.98072 | 0.5091 | 1.06027 |
| O | 0.00492 | 2.55894 | 1.5807 | 0.0901 | 2.63854 | 1.48059 | -0.04376 | 2.62516 | 1.44663 | -0.86777 | 1.91094 | -2.01878 |
| C | -0.2704 | 1.66684 | -2.1258 | -0.27489 | 1.67249 | -2.19618 | -0.2641 | 1.63248 | -2.189 | 1.20236 | 2.51359 | 1.13081 |
| C | -2.07565 | 2.38781 | -0.44646 | -2.06335 | 2.33916 | -0.4722 | -2.06079 | 2.3538 | -0.4914 | 1.92261 | 2.07277 | -1.29543 |
| H | -1.64554 | 0.82818 | 1.00477 | -1.46336 | 0.83762 | 0.98304 | -1.51352 | 0.85185 | 0.9901 | 0.82509 | 0.21108 | -1.48276 |
| O | -3.69063 | 0.01519 | -1.18023 | -3.51917 | -0.04426 | -1.29146 | -3.60029 | -0.02521 | -1.2122 | 3.7151 | -0.01828 | -0.18994 |
| C | -3.1 | -1.47042 | 1.33176 | -3.07561 | -1.39735 | 1.36713 | -3.06554 | -1.42664 | 1.37682 | 2.18289 | -2.25989 | -1.32737 |
| C | -2.7947 | -2.68975 | -0.82562 | -2.7648 | -2.72742 | -0.72593 | -2.78199 | -2.71643 | -0.74402 | 2.65275 | -2.42711 | 1.11825 |
| C | 1.67012 | -0.03888 | 2.84219 | 1.57116 | 0.15792 | 2.86695 | 1.57943 | 0.11908 | 2.85767 | -1.38624 | 0.43326 | 3.06145 |
| C | 4.74419 | -1.47909 | -0.95728 | 4.61139 | -1.61239 | -0.94889 | 4.65476 | -1.5885 | -0.92352 | -2.82455 | -1.92097 | -2.01489 |
| C | 3.36025 | -0.89066 | -1.03083 | 3.24574 | -0.97978 | -1.01188 | 3.28448 | -0.96775 | -1.0023 | -2.89645 | -1.24949 | -0.67187 |
| O | 2.93327 | -0.51762 | 0.19079 | 2.8575 | -0.5578 | 0.20395 | 2.88727 | -0.53133 | 0.2059 | -2.04552 | -0.19028 | -0.61867 |
| O | 2.70968 | -0.764 | -2.04317 | 2.58132 | -0.86137 | -2.01729 | 2.6233 | -0.87055 | -2.01187 | -3.59054 | -1.58511 | 0.25251 |
| H | -0.18238 | -1.78424 | -0.53419 | -0.13001 | -1.92971 | -0.40226 | -0.14764 | -1.87025 | -0.4619 | 0.10337 | -2.81347 | 0.39488 |
| H | -0.74442 | -2.97402 | 0.60284 | -0.77128 | -3.02854 | 0.7835 | -0.74633 | -3.01596 | 0.70204 | -0.1726 | -1.4003 | -0.59496 |
| H | 2.203 | 1.77046 | -0.83439 | 2.24088 | 1.70341 | -0.84813 | 2.2439 | 1.73076 | -0.84845 | -1.57114 | 2.57488 | 1.20703 |
| H | 2.43007 | 1.96321 | 0.89292 | 2.4225 | 1.95846 | 0.87688 | 2.42505 | 1.97155 | 0.87435 | -2.63201 | 2.41295 | -0.16889 |
| H | 0.66878 | 3.41011 | -0.09937 | 0.76335 | 3.41719 | -0.306 | 0.72076 | 3.41742 | -0.29822 | -0.6027 | 3.47684 | -0.78612 |
| H | -1.80646 | -0.42632 | -1.70949 | -1.61286 | -0.53349 | -1.65448 | -1.70724 | -0.49998 | -1.67239 | 2.23457 | 0.15651 | 1.14281 |
| H | -0.48983 | -1.53183 | 2.52463 | -0.4585 | -1.52093 | 2.63148 | -0.45768 | -1.53639 | 2.58595 | 0.34042 | -1.35935 | 2.43708 |
| H | -0.13522 | 1.68413 | 1.96312 | -0.52284 | 3.38056 | 1.51747 | 0.54631 | 3.2328 | 1.9045 | -1.24063 | 1.0249 | -1.92748 |
| H | 0.45883 | 0.86376 | -2.2555 | 0.48277 | 0.89521 | -2.32312 | 0.48832 | 0.84815 | -2.30253 | 0.69316 | 1.95477 | 1.9179 |
| H | -1.13007 | 1.44041 | -2.75817 | -1.14131 | 1.39324 | -2.7977 | -1.12673 | 1.35516 | -2.79634 | 2.27514 | 2.43465 | 1.31341 |
| H | 0.18022 | 2.58594 | -2.51416 | 0.12769 | 2.59933 | -2.61765 | 0.14944 | 2.55136 | -2.61738 | 0.93437 | 3.56984 | 1.23801 |
| H | -2.69652 | 2.54455 | -1.3208 | -2.71 | 2.4509 | -1.33413 | -2.70215 | 2.46313 | -1.35822 | 2.89571 | 2.42773 | -0.97651 |
| H | -2.23143 | 3.08662 | 0.3664 | -2.27026 | 3.04285 | 0.32725 | -2.22062 | 3.07524 | 0.29972 | 1.64471 | 2.32235 | -2.31202 |
| H | -4.10174 | 0.56525 | -0.50362 | -3.99608 | 0.48397 | -0.64096 | -4.02698 | 0.54023 | -0.55836 | 3.81023 | 0.04437 | -1.14739 |
| H | -2.79914 | -0.65185 | 1.98936 | -3.05634 | -2.30133 | 1.98239 | -2.74113 | -0.59819 | 2.00983 | 1.82672 | -3.29275 | -1.37377 |
| H | -3.02117 | -2.39679 | 1.90758 | -4.11645 | -1.21873 | 1.08574 | -3.01637 | -2.33818 | 1.97931 | 3.25769 | -2.27503 | -1.52124 |
| H | -4.1535 | -1.33 | 1.08072 | -2.74803 | -0.567 | 1.99591 | -4.11414 | -1.26338 | 1.11731 | 1.69926 | -1.70946 | -2.13932 |
| H | -2.82675 | -3.63195 | -0.27141 | -2.8349 | -3.64394 | -0.13344 | -2.83356 | -3.6425 | -0.16448 | 2.39695 | -3.49027 | 1.09019 |
| H | -3.80145 | -2.45718 | -1.17272 | -3.75695 | -2.48015 | -1.1024 | -3.78301 | -2.47279 | -1.09921 | 3.72651 | -2.3275 | 0.95269 |
| H | -2.16071 | -2.84064 | -1.70522 | -2.12022 | -2.93438 | -1.58637 | -2.15031 | -2.90445 | -1.61832 | 2.44031 | -2.05727 | 2.12473 |
| H | 1.7532 | 1.04507 | 2.95872 | 1.15349 | -0.31898 | 3.75566 | 2.66163 | -0.04414 | 2.8514 | -0.70987 | 0.00931 | 3.80412 |
| H | 1.14551 | -0.4361 | 3.71279 | 2.65227 | -0.01505 | 2.86557 | 1.4112 | 1.19204 | 2.97212 | -1.31261 | 1.52299 | 3.13154 |
| H | 2.69164 | -0.43058 | 2.85339 | 1.41036 | 1.23501 | 2.94647 | 1.16565 | -0.38238 | 3.7343 | -2.41121 | 0.1713 | 3.34665 |
| H | 5.4425 | -0.73859 | -0.56115 | 5.34687 | -0.87412 | -0.62128 | 5.37899 | -0.84906 | -0.57464 | -3.59832 | -2.68174 | -2.08658 |
| H | 4.75307 | -2.3321 | -0.27575 | 4.6168 | -2.42351 | -0.21826 | 4.65351 | -2.40819 | -0.20223 | -2.94155 | -1.19176 | -2.81831 |
| H | 5.06457 | -1.79321 | -1.94811 | 4.88732 | -1.99322 | -1.92971 | 4.94979 | -1.95747 | -1.90331 | -1.84523 | -2.39075 | -2.13651 |

## Table S29. Optimized cartesian coordinates of conformers of (1*S,*2*R,*4*R,*5*S,*10*R*)-2 at B3LYP/6-311G(d) level in chloroform.

|  | (1*S,*2*R,*4*R,*5*S,*10*R*)-2-5 | | | (1*S,*2*R,*4*R,*5*S,*10*R*)-2-6 | | | (1*S,*2*R,*4*R,*5*S,*10*R*)-2-7 | | |
| --- | --- | --- | --- | --- | --- | --- | --- | --- | --- |
| C | -0.48282 | -1.85159 | 0.5272 | -0.73749 | -1.97211 | 0.63029 | -0.47659 | -1.91761 | 0.59997 |
| C | 1.59044 | 0.38285 | 0.04095 | 1.50702 | 0.08647 | 0.30552 | 1.54759 | 0.35252 | 0.09558 |
| C | 1.55485 | 1.89252 | -0.19847 | 1.73487 | 1.58225 | 0.06958 | 1.5726 | 1.85751 | -0.17294 |
| C | 0.17963 | 2.56337 | -0.03423 | 0.48593 | 2.46974 | 0.05765 | 0.21904 | 2.58631 | -0.10194 |
| C | -0.94674 | 1.86023 | -0.79685 | -0.6592 | 1.89339 | -0.77576 | -0.9036 | 1.86424 | -0.83376 |
| C | -1.80863 | 0.86334 | -0.03945 | -1.60879 | 0.93701 | -0.08258 | -1.7229 | 0.85981 | -0.04227 |
| C | -2.26516 | -0.44871 | -0.64101 | -2.11233 | -0.32724 | -0.753 | -2.16493 | -0.46275 | -0.63963 |
| C | -2.01195 | -1.67007 | 0.29218 | -2.17801 | -1.55625 | 0.20142 | -1.99084 | -1.67491 | 0.32206 |
| C | 0.20988 | -1.01742 | 1.57232 | -0.05822 | -1.20202 | 1.73242 | 0.20972 | -1.07001 | 1.6373 |
| C | 1.16478 | -0.09041 | 1.41013 | 0.95134 | -0.32864 | 1.6431 | 1.11902 | -0.10347 | 1.4678 |
| H | 1.00539 | -0.11757 | -0.72087 | 0.89519 | -0.30592 | -0.4972 | 0.9521 | -0.13835 | -0.66405 |
| O | -0.1432 | 2.77569 | 1.34343 | 0.113 | 2.7048 | 1.41946 | -0.19026 | 2.80793 | 1.25268 |
| C | -0.65023 | 1.62608 | -2.2656 | -0.30156 | 1.66789 | -2.23181 | -0.61696 | 1.61123 | -2.30041 |
| C | -2.36839 | 2.20359 | -0.44822 | -2.0755 | 2.31571 | -0.48358 | -2.3259 | 2.18008 | -0.4605 |
| H | -1.60295 | 0.80112 | 1.01793 | -1.41333 | 0.84976 | 0.97422 | -1.45834 | 0.82555 | 1.00232 |
| O | -3.65494 | -0.32042 | -0.98186 | -3.39256 | -0.04017 | -1.3392 | -3.52957 | -0.32073 | -1.07106 |
| C | -2.78395 | -1.53988 | 1.61032 | -3.07669 | -1.27791 | 1.41122 | -2.78882 | -1.48258 | 1.61642 |
| C | -2.50031 | -2.93358 | -0.43953 | -2.76456 | -2.74024 | -0.58818 | -2.51461 | -2.93129 | -0.3975 |
| C | 1.91854 | 0.48507 | 2.58446 | 1.60092 | 0.26498 | 2.8632 | 1.79357 | 0.55976 | 2.6385 |
| C | 4.7027 | -1.42282 | -0.92894 | 4.52883 | -1.67792 | -0.98154 | 4.61472 | -1.51814 | -0.91691 |
| C | 3.22877 | -1.11608 | -0.89299 | 3.16091 | -1.04813 | -1.02068 | 3.15577 | -1.14259 | -0.90952 |
| O | 2.98988 | -0.00777 | -0.16551 | 2.82377 | -0.56099 | 0.18564 | 2.93918 | -0.08939 | -0.10169 |
| O | 2.37081 | -1.77642 | -1.43217 | 2.45638 | -0.98142 | -2.00355 | 2.29292 | -1.71616 | -1.53504 |
| H | 0.03284 | -1.78492 | -0.43217 | -0.11415 | -2.01197 | -0.26747 | 0.0626 | -1.8913 | -0.34923 |
| H | -0.34234 | -2.88974 | 0.85321 | -0.80312 | -3.00878 | 0.98066 | -0.38732 | -2.95247 | 0.95202 |
| H | 1.91338 | 2.06195 | -1.21756 | 2.2254 | 1.6749 | -0.90248 | 1.98739 | 1.99123 | -1.17654 |
| H | 2.26174 | 2.39627 | 0.46465 | 2.43184 | 1.97796 | 0.81117 | 2.27501 | 2.34703 | 0.50845 |
| H | 0.27142 | 3.57674 | -0.43772 | 0.78035 | 3.4291 | -0.3923 | 0.35686 | 3.56336 | -0.58737 |
| H | -1.70469 | -0.64672 | -1.56345 | -1.42691 | -0.60954 | -1.56334 | -1.55458 | -0.68966 | -1.52367 |
| H | -0.04581 | -1.27765 | 2.59846 | -0.41777 | -1.43605 | 2.73318 | -0.04396 | -1.3239 | 2.6655 |
| H | -0.17716 | 1.91706 | 1.78245 | -0.53646 | 3.41616 | 1.43661 | 0.38882 | 3.47621 | 1.63366 |
| H | -1.53649 | 1.27557 | -2.79672 | 0.46619 | 0.90032 | -2.36024 | -1.49804 | 1.21814 | -2.80961 |
| H | -0.33822 | 2.5616 | -2.74063 | -1.17618 | 1.36253 | -2.80866 | -0.34131 | 2.5446 | -2.80178 |
| H | 0.14295 | 0.89211 | -2.43201 | 0.07652 | 2.59335 | -2.67845 | 0.19807 | 0.89898 | -2.45886 |
| H | -3.09019 | 2.2193 | -1.25486 | -2.74988 | 2.3887 | -1.32642 | -3.06163 | 2.15974 | -1.25413 |
| H | -2.54393 | 2.91742 | 0.34739 | -2.28979 | 3.01779 | 0.31565 | -2.50433 | 2.90419 | 0.32408 |
| H | -3.88681 | -1.04875 | -1.56832 | -3.58975 | -0.73347 | -1.97818 | -3.73324 | -1.05724 | -1.65756 |
| H | -3.84882 | -1.40996 | 1.41054 | -2.67986 | -0.48885 | 2.05191 | -3.8409 | -1.30562 | 1.38545 |
| H | -2.45356 | -0.6898 | 2.20995 | -3.18714 | -2.17663 | 2.0252 | -2.43014 | -0.63728 | 2.20585 |
| H | -2.65762 | -2.43934 | 2.21989 | -4.06935 | -0.96985 | 1.07706 | -2.72318 | -2.37497 | 2.24606 |
| H | -2.00142 | -3.05214 | -1.40692 | -2.75786 | -3.64884 | 0.01881 | -2.00467 | -3.08531 | -1.35446 |
| H | -2.29067 | -3.82786 | 0.15226 | -3.80208 | -2.55865 | -0.87779 | -2.34617 | -3.82217 | 0.21243 |
| H | -3.57963 | -2.9119 | -0.60907 | -2.18354 | -2.94412 | -1.49413 | -3.58963 | -2.87376 | -0.58451 |
| H | 1.54635 | 0.08253 | 3.52791 | 2.6836 | 0.10246 | 2.84327 | 1.49918 | 1.60841 | 2.71741 |
| H | 2.98166 | 0.23855 | 2.50627 | 1.43079 | 1.34246 | 2.9087 | 1.53388 | 0.06545 | 3.57615 |
| H | 1.85657 | 1.57552 | 2.63082 | 1.20841 | -0.18507 | 3.77698 | 2.88224 | 0.5323 | 2.52948 |
| H | 4.90914 | -2.15058 | -1.71093 | 4.58182 | -2.41872 | -0.18179 | 5.244 | -0.63109 | -0.99738 |
| H | 5.28522 | -0.51525 | -1.09025 | 4.74761 | -2.14511 | -1.93923 | 4.86169 | -2.01224 | 0.02665 |
| H | 5.00478 | -1.84121 | 0.03503 | 5.27983 | -0.91353 | -0.76823 | 4.81614 | -2.2017 | -1.73907 |

## Table S30. Optimized cartesian coordinates of conformers of (1*S,*2*R,*4*R,*5*S,*10*S*)-2 at B3LYP/6-311G(d) level in chloroform.

|  | (1*S,*2*R,*4*R,*5*S,*10*S*)-2-1 | | | (1*S,*2*R,*4*R,*5*S,*10*S*)-2-2 | | | (1*S,*2*R,*4*R,*5*S,*10*S*)-2-3 | | | (1*S,*2*R,*4*R,*5*S,*10*S*)-2-4 | | |
| --- | --- | --- | --- | --- | --- | --- | --- | --- | --- | --- | --- | --- |
| C | 0.93967 | -1.89672 | -0.49694 | 0.46605 | -1.87379 | -0.18488 | 0.45826 | -1.88923 | -0.19208 | 0.94151 | -1.89764 | -0.51589 |
| C | -1.51852 | -0.23986 | 0.38417 | -1.61065 | 0.41902 | 0.1464 | -1.60578 | 0.41692 | 0.14317 | -1.51134 | -0.24361 | 0.38594 |
| C | -1.68873 | 1.21164 | 0.87436 | -1.45257 | 1.9354 | 0.29485 | -1.44747 | 1.93365 | 0.30413 | -1.67877 | 1.20699 | 0.88128 |
| C | -0.7737 | 2.21093 | 0.15445 | -0.22183 | 2.51383 | -0.42118 | -0.22705 | 2.524 | -0.40484 | -0.7772 | 2.2136 | 0.1527 |
| C | 0.71142 | 1.97188 | 0.41903 | 1.11841 | 1.99585 | 0.08086 | 1.12378 | 1.99836 | 0.08331 | 0.71128 | 1.98449 | 0.40264 |
| C | 1.46593 | 1.02993 | -0.49646 | 1.71919 | 0.77854 | -0.59382 | 1.70312 | 0.77009 | -0.58503 | 1.45843 | 1.03439 | -0.51386 |
| C | 2.49292 | 0.04777 | 0.04879 | 2.43355 | -0.30601 | 0.19927 | 2.418 | -0.31766 | 0.20493 | 2.4708 | 0.05297 | 0.04402 |
| C | 2.39591 | -1.36011 | -0.60082 | 2.01453 | -1.7399 | -0.22609 | 2.00635 | -1.74986 | -0.23177 | 2.39273 | -1.34659 | -0.62453 |
| C | 0.32746 | -1.84627 | 0.87332 | -0.17453 | -1.41019 | 1.08941 | -0.18119 | -1.42528 | 1.08258 | 0.33512 | -1.85683 | 0.85754 |
| C | -0.73953 | -1.1547 | 1.29318 | -1.06068 | -0.42465 | 1.2737 | -1.05882 | -0.43257 | 1.26826 | -0.72735 | -1.16349 | 1.28637 |
| H | -1.07928 | -0.22895 | -0.6062 | -1.20239 | 0.11543 | -0.81046 | -1.20148 | 0.11301 | -0.81494 | -1.08036 | -0.22978 | -0.60797 |
| O | -1.02759 | 2.22221 | -1.24968 | -0.30221 | 2.25858 | -1.82735 | -0.43304 | 2.29355 | -1.80309 | -1.04519 | 2.22362 | -1.24882 |
| C | 1.09284 | 2.01753 | 1.8847 | 1.33777 | 2.19906 | 1.56589 | 1.36607 | 2.21421 | 1.56337 | 1.1059 | 2.04116 | 1.86436 |
| C | 1.71093 | 2.52484 | -0.55877 | 2.32019 | 2.15297 | -0.81107 | 2.32198 | 2.13212 | -0.8208 | 1.70447 | 2.52239 | -0.59034 |
| H | 0.88261 | 0.68202 | -1.33762 | 1.15387 | 0.43347 | -1.44772 | 1.1204 | 0.42884 | -1.42959 | 0.86795 | 0.67581 | -1.34469 |
| O | 3.83181 | 0.56167 | -0.02342 | 3.86134 | -0.14928 | 0.17203 | 3.84434 | -0.15294 | 0.18934 | 3.7847 | 0.6241 | -0.06824 |
| C | 2.7905 | -1.31037 | -2.08305 | 2.49281 | -2.05508 | -1.65012 | 2.48725 | -2.05205 | -1.65777 | 2.78468 | -1.27105 | -2.10492 |
| C | 3.3567 | -2.30252 | 0.13931 | 2.65802 | -2.74103 | 0.74504 | 2.65242 | -2.75543 | 0.73307 | 3.36082 | -2.29718 | 0.09994 |
| C | -1.25333 | -1.29246 | 2.70381 | -1.63247 | -0.14478 | 2.64208 | -1.62553 | -0.15014 | 2.63841 | -1.23248 | -1.30492 | 2.69979 |
| C | -4.87564 | -1.20464 | -0.90778 | -4.96302 | -1.05629 | -0.54855 | -4.96757 | -1.03149 | -0.55973 | -4.88 | -1.21119 | -0.8766 |
| C | -3.55066 | -0.49762 | -0.84505 | -3.474 | -0.86214 | -0.67461 | -3.47487 | -0.85755 | -0.67278 | -3.55586 | -0.5014 | -0.82552 |
| O | -2.84489 | -0.86249 | 0.22984 | -3.06098 | 0.17358 | 0.0813 | -3.05663 | 0.17758 | 0.08014 | -2.83898 | -0.8671 | 0.24167 |
| O | -3.16734 | 0.31928 | -1.66159 | -2.74198 | -1.54726 | -1.34938 | -2.74526 | -1.55611 | -1.33651 | -3.18255 | 0.31781 | -1.64415 |
| H | 0.96413 | -2.94589 | -0.81711 | 0.23238 | -2.93783 | -0.31626 | 0.22776 | -2.95386 | -0.32297 | 0.97024 | -2.94397 | -0.84419 |
| H | 0.31303 | -1.38518 | -1.22774 | 0.02571 | -1.37476 | -1.0461 | 0.01569 | -1.39214 | -1.0533 | 0.30934 | -1.38387 | -1.23967 |
| H | -1.53442 | 1.28455 | 1.95046 | -1.45329 | 2.24299 | 1.34208 | -1.44164 | 2.22649 | 1.35466 | -1.51085 | 1.2777 | 1.95554 |
| H | -2.72137 | 1.52412 | 0.70243 | -2.33778 | 2.39937 | -0.15159 | -2.32518 | 2.40204 | -0.14516 | -2.7151 | 1.51523 | 0.72407 |
| H | -1.01518 | 3.20784 | 0.55125 | -0.23456 | 3.60106 | -0.2545 | -0.24266 | 3.60726 | -0.21729 | -1.02381 | 3.2082 | 0.55295 |
| H | 2.32953 | -0.0932 | 1.1189 | 2.18504 | -0.20898 | 1.25746 | 2.16241 | -0.22662 | 1.26185 | 2.2624 | -0.10608 | 1.1081 |
| H | 0.81736 | -2.47642 | 1.61339 | 0.12467 | -1.96344 | 1.97832 | 0.11421 | -1.98211 | 1.97054 | 0.82747 | -2.49232 | 1.59185 |
| H | -1.78892 | 1.65345 | -1.44677 | -1.09878 | 2.68268 | -2.16487 | 0.16079 | 2.86659 | -2.29937 | -1.80804 | 1.65446 | -1.43801 |
| H | 0.6875 | 2.91737 | 2.35831 | 1.12363 | 3.23514 | 1.84704 | 2.40208 | 2.00355 | 1.83234 | 0.73596 | 1.18053 | 2.42887 |
| H | 0.7184 | 1.15089 | 2.43609 | 0.70247 | 1.54771 | 2.16961 | 1.16234 | 3.25416 | 1.8379 | 2.19061 | 2.06544 | 1.97898 |
| H | 2.17592 | 2.04173 | 2.01214 | 2.3729 | 1.99548 | 1.843 | 0.73044 | 1.57334 | 2.17803 | 0.70742 | 2.9459 | 2.3347 |
| H | 2.63737 | 2.90925 | -0.14829 | 3.2634 | 2.37445 | -0.32559 | 3.27092 | 2.34663 | -0.34462 | 2.64535 | 2.89252 | -0.20386 |
| H | 1.34181 | 3.0388 | -1.43726 | 2.18401 | 2.59759 | -1.78864 | 2.2183 | 2.56186 | -1.81153 | 1.32862 | 3.02691 | -1.47162 |
| H | 3.93482 | 0.98966 | -0.88139 | 4.11482 | 0.06187 | -0.73411 | 4.10978 | 0.02445 | -0.72074 | 4.37402 | 0.12913 | 0.5113 |
| H | 2.16823 | -0.6135 | -2.65094 | 2.10649 | -1.34075 | -2.38206 | 2.10166 | -1.33122 | -2.38376 | 2.77246 | -2.26636 | -2.55855 |
| H | 2.6826 | -2.2969 | -2.54221 | 2.15537 | -3.04957 | -1.95454 | 2.15021 | -3.04362 | -1.97168 | 3.78845 | -0.8574 | -2.21284 |
| H | 3.83367 | -1.00825 | -2.20168 | 3.58392 | -2.04833 | -1.71118 | 3.5786 | -2.04595 | -1.71818 | 2.10021 | -0.63804 | -2.67479 |
| H | 3.33954 | -3.3024 | -0.30388 | 3.74348 | -2.63505 | 0.73513 | 2.3122 | -2.60613 | 1.76129 | 3.26601 | -3.31548 | -0.2862 |
| H | 4.37671 | -1.92028 | 0.08723 | 2.32151 | -2.58215 | 1.77309 | 2.40846 | -3.78178 | 0.44433 | 4.40103 | -1.99655 | -0.05021 |
| H | 3.09522 | -2.39855 | 1.19653 | 2.40896 | -3.76869 | 0.46514 | 3.7374 | -2.6447 | 0.72722 | 3.17294 | -2.32976 | 1.17708 |
| H | -2.31286 | -1.56397 | 2.70562 | -2.72513 | -0.18312 | 2.62432 | -1.33421 | 0.83592 | 3.0142 | -0.68356 | -2.07949 | 3.2372 |
| H | -0.70401 | -2.06191 | 3.248 | -1.27742 | -0.87378 | 3.37195 | -2.71867 | -0.16625 | 2.62018 | -1.13636 | -0.37568 | 3.27023 |
| H | -1.16587 | -0.36064 | 3.27139 | -1.36113 | 0.84953 | 3.011 | -1.28536 | -0.89083 | 3.36354 | -2.29332 | -1.57093 | 2.70778 |
| H | -4.74726 | -2.275 | -0.74186 | -5.17473 | -1.58251 | 0.38654 | -5.47649 | -0.06734 | -0.5742 | -5.52286 | -0.83436 | -0.07681 |
| H | -5.52539 | -0.82501 | -0.11493 | -5.48381 | -0.09917 | -0.51436 | -5.32682 | -1.66263 | -1.37013 | -5.35872 | -1.03017 | -1.83632 |
| H | -5.34469 | -1.02444 | -1.87242 | -5.32752 | -1.65854 | -1.37849 | -5.20003 | -1.51423 | 0.39359 | -4.7478 | -2.2816 | -0.71402 |

## Table S31. Optimized cartesian coordinates of conformers of (1*R,*2*R,*4*S,*5*S,*10*R*)-3 and (1*R,*2*R,*4*S,*5*S,*10*R*)-3at B3LYP/6-311G(d) level in chloroform.

|  | (1*R,*2*R,*4*S,*5*S,*10*R*)-3-1 | | | (1*R,*2*R,*4*S,*5*S,*10*R*)-3-2 | | |  | (1*R,*2*R,*4*S,*5*S,*10*S)*-3-1 | | | (1*R,*2*R,*4*S,*5*S,*10*S)*-3-2 | | |
| --- | --- | --- | --- | --- | --- | --- | --- | --- | --- | --- | --- | --- | --- |
| C | 0.36468 | 1.71217 | 1.17856 | -0.19306 | -1.90701 | -1.06614 |  | -0.28021 | 1.80785 | -0.89047 | 0.43904 | 2.07407 | 0.85237 |
| C | -0.38707 | 2.38544 | 0.10081 | -1.51174 | -2.15167 | -0.45252 |  | 0.84089 | 2.40123 | -0.14448 | -0.33414 | 2.47973 | -0.33267 |
| C | -2.50887 | 1.19154 | -0.47602 | -2.62247 | 0.06475 | -0.09945 |  | 2.72052 | 0.75904 | -0.08372 | -2.28307 | 0.93708 | -0.65261 |
| C | -2.53417 | -0.11032 | 0.2008 | -1.85802 | 1.2007 | -0.6192 |  | 2.3853 | -0.49626 | -0.75829 | -2.28809 | -0.14874 | 0.3287 |
| C | -2.45549 | -1.46342 | -0.49269 | -1.16403 | 2.26633 | 0.21413 |  | 2.21215 | -1.85489 | -0.09889 | -2.0226 | -1.61126 | 0.00793 |
| C | -0.95799 | -1.88242 | -0.57423 | 0.29073 | 1.77684 | 0.46611 |  | 0.70281 | -2.06082 | 0.20867 | -0.48202 | -1.81223 | 0.01166 |
| C | -0.24308 | -1.98405 | 0.74456 | 0.44597 | 0.66455 | 1.46847 |  | 0.13125 | -1.23348 | 1.32417 | 0.20564 | -1.52771 | 1.31781 |
| C | 0.84972 | -1.33237 | 1.17254 | 1.09898 | -0.50581 | 1.40684 |  | -0.90489 | -0.38625 | 1.31811 | 1.24882 | -0.74417 | 1.61429 |
| C | 1.58531 | -0.31213 | 0.33614 | 1.85993 | -1.10412 | 0.23758 |  | -1.67987 | -0.03605 | 0.07159 | 2.07852 | 0.11432 | 0.678 |
| C | 1.71836 | 1.0729 | 0.98258 | 1.13784 | -2.2706 | -0.45381 |  | -1.63012 | 1.44462 | -0.32946 | 1.87779 | 1.62487 | 0.85977 |
| O | 2.92469 | -0.85211 | 0.11584 | 2.18911 | -0.15313 | -0.80209 |  | -3.06997 | -0.39485 | 0.33569 | 1.86182 | -0.16626 | -0.72419 |
| C | 3.55671 | -0.44305 | -1.00682 | 3.30003 | 0.60138 | -0.59197 |  | -3.79445 | -0.83263 | -0.7187 | 2.61592 | -1.15342 | -1.27212 |
| O | 3.08186 | 0.33201 | -1.81049 | 4.01154 | 0.49526 | 0.38018 |  | -3.36169 | -0.93141 | -1.83932 | 3.4482 | -1.77681 | -0.66839 |
| C | 4.92021 | -1.08188 | -1.11227 | 3.49257 | 1.58316 | -1.72078 |  | -5.19931 | -1.17068 | -0.29052 | 2.24432 | -1.35559 | -2.71788 |
| C | -1.90527 | 2.40083 | 0.20534 | -2.65815 | -1.23836 | -0.86428 |  | 2.24209 | 2.08114 | -0.63462 | -1.84524 | 2.32759 | -0.26311 |
| O | -3.73628 | 0.66672 | 0.06255 | -3.29384 | 1.19121 | -0.69424 |  | 3.7098 | 0.05315 | -0.85164 | -3.52042 | 0.45444 | -0.09818 |
| C | -3.27583 | -2.46789 | 0.33747 | -1.10064 | 3.54828 | -0.63762 |  | 2.64649 | -2.91574 | -1.1251 | -2.72623 | -2.47617 | 1.06551 |
| C | -3.02778 | -1.41041 | -1.91646 | -1.92191 | 2.57285 | 1.51238 |  | 3.07762 | -1.99968 | 1.15812 | -2.55631 | -1.99474 | -1.37826 |
| C | 1.4318 | -1.60589 | 2.5382 | 1.18827 | -1.38071 | 2.63827 |  | -1.40945 | 0.22731 | 2.59961 | 1.75282 | -0.66495 | 3.03695 |
| O | 0.28865 | 3.1464 | 1.12416 | -1.01759 | -3.00209 | -1.50846 |  | 0.04678 | 3.19749 | -1.04755 | 0.19605 | 3.46488 | 0.57841 |
| C | 0.1705 | 2.53641 | -1.29858 | -1.67123 | -2.81347 | 0.89777 |  | 0.73463 | 2.78866 | 1.31005 | 0.2595 | 2.48953 | -1.7201 |
| H | -0.22956 | 1.27603 | 1.98454 | -0.13574 | -1.08837 | -1.78579 |  | -0.00837 | 1.21375 | -1.76419 | -0.14272 | 1.6984 | 1.69337 |
| H | -2.15743 | -0.12893 | 1.22545 | -1.38194 | 1.05029 | -1.59297 |  | 1.82968 | -0.40293 | -1.69321 | -2.01917 | 0.12704 | 1.34801 |
| H | -2.49155 | 1.20218 | -1.56509 | -2.77133 | -0.00812 | 0.97732 |  | 2.91066 | 0.72756 | 0.98618 | -2.16079 | 0.67525 | -1.70106 |
| H | -0.44766 | -1.18941 | -1.25013 | 0.86473 | 2.64069 | 0.83668 |  | 0.57486 | -3.11589 | 0.48484 | -0.07304 | -1.19757 | -0.7842 |
| H | -0.92543 | -2.86871 | -1.06099 | 0.72325 | 1.50174 | -0.49614 |  | 0.13996 | -1.92957 | -0.71883 | -0.28776 | -2.86004 | -0.25721 |
| H | -0.67606 | -2.70583 | 1.43739 | 0.00557 | 0.89154 | 2.43898 |  | 0.59975 | -1.40793 | 2.29101 | -0.20371 | -2.08594 | 2.15806 |
| H | 1.12278 | -0.19185 | -0.64192 | 2.80926 | -1.481 | 0.63201 |  | -1.35432 | -0.63361 | -0.77629 | 3.12713 | -0.11562 | 0.88129 |
| H | 2.23891 | 0.9914 | 1.94306 | 1.79543 | -2.64804 | -1.24631 |  | -2.37767 | 1.606 | -1.11154 | 2.32249 | 1.92242 | 1.81459 |
| H | 2.33114 | 1.70273 | 0.33551 | 1.01511 | -3.08564 | 0.26319 |  | -1.90093 | 2.08688 | 0.5084 | 2.44634 | 2.142 | 0.08622 |
| H | 4.8284 | -2.17218 | -1.08543 | 3.34408 | 1.09651 | -2.6885 |  | -5.18051 | -1.92444 | 0.49944 | 3.00126 | -1.96439 | -3.20769 |
| H | 5.39826 | -0.76825 | -2.04055 | 2.74791 | 2.38288 | -1.6341 |  | -5.68798 | -0.28372 | 0.11903 | 1.28273 | -1.8735 | -2.77385 |
| H | 5.53705 | -0.78471 | -0.25775 | 4.49101 | 2.01714 | -1.65945 |  | -5.76232 | -1.54065 | -1.14447 | 2.13389 | -0.39902 | -3.23033 |
| H | -2.29962 | 3.32513 | -0.23671 | -3.61757 | -1.74819 | -0.70632 |  | 2.93403 | 2.8826 | -0.35235 | -2.32341 | 3.07276 | -0.90908 |
| H | -2.19456 | 2.39411 | 1.26077 | -2.5759 | -1.02412 | -1.93434 |  | 2.2371 | 2.03057 | -1.72562 | -2.17543 | 2.53019 | 0.75802 |
| H | -4.33308 | -2.18325 | 0.33226 | -0.53744 | 3.38005 | -1.56396 |  | 2.55792 | -3.92275 | -0.70827 | -2.43564 | -2.2012 | 2.08209 |
| H | -2.94724 | -2.49961 | 1.38175 | -0.60944 | 4.35863 | -0.08687 |  | 3.68633 | -2.7603 | -1.4214 | -2.49374 | -3.53563 | 0.92374 |
| H | -3.19091 | -3.47917 | -0.07732 | -2.10819 | 3.87965 | -0.91027 |  | 2.02933 | -2.87228 | -2.02754 | -3.80982 | -2.35579 | 0.99244 |
| H | -4.03358 | -0.98062 | -1.9095 | -2.00948 | 1.69893 | 2.16377 |  | 2.81298 | -1.28262 | 1.9367 | -3.60714 | -1.71994 | -1.47848 |
| H | -2.39991 | -0.80991 | -2.58401 | -1.40418 | 3.3572 | 2.07607 |  | 2.96694 | -3.00178 | 1.58115 | -1.999 | -1.5038 | -2.18047 |
| H | -3.08503 | -2.41967 | -2.33841 | -2.93323 | 2.92049 | 1.28436 |  | 4.12919 | -1.84802 | 0.91131 | -2.46662 | -3.07314 | -1.53191 |
| H | 1.35914 | -0.7289 | 3.19456 | 2.22932 | -1.45375 | 2.98045 |  | -2.42889 | -0.11 | 2.80414 | 1.17825 | -1.31426 | 3.69832 |
| H | 0.91305 | -2.43371 | 3.02983 | 0.58965 | -0.97497 | 3.45841 |  | -0.78061 | -0.05287 | 3.44632 | 2.80467 | -0.96446 | 3.09901 |
| H | 2.49544 | -1.85906 | 2.46283 | 0.84393 | -2.40417 | 2.44862 |  | -1.44389 | 1.31824 | 2.55517 | 1.69066 | 0.35266 | 3.43762 |
| H | -0.11051 | 3.51602 | -1.70276 | -1.66731 | -2.06608 | 1.6986 |  | 1.40871 | 3.62391 | 1.52048 | -0.20486 | 3.27997 | -2.31706 |
| H | 1.25708 | 2.44948 | -1.32854 | -2.62508 | -3.35179 | 0.9379 |  | -0.27458 | 3.10826 | 1.56372 | 1.33246 | 2.66992 | -1.69743 |
| H | -0.24095 | 1.76992 | -1.96503 | -0.87462 | -3.53403 | 1.09074 |  | 1.01037 | 1.95639 | 1.96193 | 0.08968 | 1.53451 | -2.22259 |

## Table S32. Optimized cartesian coordinates of conformers of (1*R,*2*R,*4*R,*5*R,*10*R*)-3 at B3LYP/6-311G(d) level in chloroform.

|  | (1*R,*2*R,*4*R,*5*R,*10*R*)-3-1 | | | (1*R,*2*R,*4*R,*5*R,*10*R*)-3-2 | | | (1*R,*2*R,*4*R,*5*R,*10*R*)-3-3 | | | (1*R,*2*R,*4*R,*5*R,*10*R*)-3-4 | | | (1*R,*2*R,*4*R,*5*R,*10*R*)-3-5 | | | (1*R,*2*R,*4*R,*5*R,*10*R*)-3-6 | | |
| --- | --- | --- | --- | --- | --- | --- | --- | --- | --- | --- | --- | --- | --- | --- | --- | --- | --- | --- |
| C | 0.49279 | 1.99375 | 0.9086 | -0.40939 | -1.69808 | -0.874 | 0.02592 | 2.01132 | -1.00728 | -0.80475 | 1.71486 | -0.86789 | 0.15906 | 1.94935 | 0.83417 | -0.27834 | 2.15822 | 0.53886 |
| C | -0.37088 | 2.37094 | -0.2222 | -1.73758 | -2.09542 | -0.36809 | 1.3268 | 2.22492 | -0.34615 | 0.45126 | 2.38292 | -0.48319 | -0.86317 | 2.40103 | -0.12437 | -1.45101 | 2.26389 | -0.34463 |
| C | -2.40339 | 1.00446 | 0.33627 | -2.53031 | 0.30351 | -0.73197 | 2.34099 | -0.01737 | -0.95947 | 2.04443 | 0.5679 | -1.259 | -2.76831 | 0.73159 | 0.16971 | -2.86604 | 0.2306 | 0.25672 |
| C | -2.56156 | -0.03473 | -0.68295 | -2.05195 | 1.17535 | 0.34011 | 2.26245 | -0.86321 | 0.22975 | 2.50959 | -0.13548 | -0.06476 | -2.02589 | -0.31516 | -0.55093 | -1.92807 | -0.67739 | -0.42282 |
| C | -2.4462 | -1.53582 | -0.43519 | -1.04339 | 2.30412 | 0.19159 | 1.40005 | -2.11856 | 0.30998 | 2.19969 | -1.602 | 0.22184 | -2.05246 | -1.81472 | -0.27724 | -1.58049 | -2.10693 | -0.02373 |
| C | -0.93909 | -1.91196 | -0.30521 | 0.38256 | 1.68543 | 0.24456 | -0.08482 | -1.66879 | 0.3388 | 0.68484 | -1.72504 | 0.53826 | -0.60545 | -2.26691 | 0.06527 | -0.04984 | -2.17654 | 0.23447 |
| C | -0.31122 | -1.66485 | 1.03662 | 0.6138 | 0.73607 | 1.38926 | -0.44429 | -0.71043 | 1.43985 | 0.19096 | -0.85218 | 1.65743 | -0.00747 | -1.7345 | 1.33773 | 0.48522 | -1.37531 | 1.38797 |
| C | 0.78031 | -0.9632 | 1.36391 | 1.19094 | -0.47013 | 1.41042 | -1.22154 | 0.37716 | 1.42039 | -0.90652 | -0.09314 | 1.75695 | 0.96481 | -0.82648 | 1.49156 | 1.21571 | -0.25284 | 1.36279 |
| C | 1.58634 | -0.18074 | 0.35911 | 1.81696 | -1.2085 | 0.24118 | -1.96704 | 0.9654 | 0.23758 | -2.00267 | 0.11221 | 0.7331 | 1.55933 | -0.06746 | 0.32986 | 1.54049 | 0.49331 | 0.08698 |
| C | 1.8138 | 1.28719 | 0.73618 | 0.90929 | -2.26145 | -0.40389 | -1.32473 | 2.21835 | -0.3671 | -1.98739 | 1.48407 | 0.03973 | 1.54064 | 1.45819 | 0.49581 | 1.1531 | 1.9758 | 0.11029 |
| O | 2.88807 | -0.8409 | 0.25869 | 2.25009 | -0.33107 | -0.82924 | -2.16547 | 0.03755 | -0.85822 | -1.97958 | -0.9218 | -0.28319 | 2.95224 | -0.49366 | 0.20619 | 2.98761 | 0.47711 | -0.12509 |
| C | 3.53347 | -0.73749 | -0.92022 | 3.40275 | 0.34691 | -0.6289 | -3.1684 | -0.86005 | -0.72285 | -3.16632 | -1.36534 | -0.75306 | 3.47794 | -0.51353 | -1.03571 | 3.49609 | -0.52568 | -0.87029 |
| O | 3.09702 | -0.14067 | -1.87719 | 4.08446 | 0.22701 | 0.35948 | -3.90206 | -0.90157 | 0.23401 | -4.23648 | -0.95437 | -0.37434 | 2.86669 | -0.20157 | -2.03109 | 2.8273 | -1.3703 | -1.41771 |
| C | 4.85681 | -1.45357 | -0.8705 | 3.69011 | 1.26076 | -1.78904 | -3.21692 | -1.78639 | -1.90719 | -2.9546 | -2.42239 | -1.80293 | 4.91354 | -0.96493 | -1.00079 | 4.99908 | -0.44948 | -0.91215 |
| C | -1.8756 | 2.38567 | 0.01114 | -2.91189 | -1.12044 | -0.41535 | 2.55178 | 1.47574 | -0.87231 | 1.72392 | 2.04497 | -1.25789 | -2.30146 | 2.17208 | 0.30608 | -2.75213 | 1.74607 | 0.24236 |
| O | -3.70993 | 0.66612 | -0.16888 | -3.38317 | 1.33474 | -0.18986 | 3.44685 | -0.86828 | -0.59077 | 3.43106 | 0.19328 | -1.12334 | -3.1971 | 0.28564 | -1.1321 | -3.2349 | -0.42563 | -0.97212 |
| C | -3.25066 | -1.96751 | 0.797 | -1.20505 | 3.04683 | -1.13967 | 1.6039 | -3.02105 | -0.91422 | 2.50544 | -2.48577 | -0.99496 | -3.03321 | -2.1949 | 0.83614 | -2.37466 | -2.58828 | 1.19448 |
| C | -3.01443 | -2.25105 | -1.6732 | -1.26361 | 3.29588 | 1.34607 | 1.78362 | -2.90734 | 1.57185 | 3.06923 | -2.0695 | 1.39988 | -2.46642 | -2.51167 | -1.5847 | -1.89381 | -3.01034 | -1.22886 |
| C | 1.2886 | -0.93453 | 2.7835 | 1.29936 | -1.2502 | 2.69711 | -1.49291 | 1.14207 | 2.69379 | -1.19862 | 0.64488 | 3.04403 | 1.53911 | -0.51536 | 2.85004 | 1.78429 | 0.33919 | 2.62814 |
| O | 0.3864 | 3.36949 | 0.50397 | -1.26295 | -2.55189 | -1.65776 | 0.78737 | 3.20861 | -1.26088 | -0.55609 | 3.08044 | -1.25515 | -0.1316 | 3.35012 | 0.69088 | -0.90846 | 3.43197 | 0.32 |
| C | 0.06185 | 2.18116 | -1.65715 | -1.89228 | -3.18422 | 0.66699 | 1.43087 | 2.67256 | 1.09138 | 0.67952 | 2.92393 | 0.90686 | -0.62507 | 2.54505 | -1.60848 | -1.37268 | 2.31398 | -1.85094 |
| H | -0.01064 | 1.79367 | 1.85339 | -0.3235 | -0.70378 | -1.30038 | 0.01417 | 1.32811 | -1.85447 | -0.74816 | 1.02174 | -1.70536 | -0.2348 | 1.53694 | 1.76217 | -0.48892 | 1.75646 | 1.52891 |
| H | -2.32855 | 0.25279 | -1.70795 | -2.0574 | 0.73117 | 1.33493 | 2.4232 | -0.36906 | 1.18689 | 2.667 | 0.47212 | 0.82513 | -1.11948 | -0.00321 | -1.06251 | -1.14699 | -0.21129 | -1.01719 |
| H | -2.24324 | 0.67388 | 1.3591 | -2.21713 | 0.50943 | -1.75175 | 1.82411 | -0.36531 | -1.85038 | 1.52086 | -0.02284 | -2.00692 | -3.44476 | 0.40603 | 0.95503 | -3.40841 | -0.16656 | 1.11024 |
| H | -0.38443 | -1.42954 | -1.11401 | 1.09535 | 2.51829 | 0.32005 | -0.69851 | -2.57411 | 0.44946 | 0.4935 | -2.77366 | 0.80649 | 0.03309 | -2.04077 | -0.79225 | 0.47556 | -1.91967 | -0.68639 |
| H | -0.86457 | -2.99017 | -0.49827 | 0.58218 | 1.20049 | -0.70741 | -0.32661 | -1.24778 | -0.63383 | 0.12498 | -1.53756 | -0.373 | -0.6209 | -3.36023 | 0.14225 | 0.19365 | -3.22789 | 0.42771 |
| H | -0.79973 | -2.19293 | 1.85288 | 0.26491 | 1.10262 | 2.35308 | -0.04378 | -0.97709 | 2.41607 | 0.8142 | -0.87738 | 2.54978 | -0.38828 | -2.19705 | 2.24666 | 0.29218 | -1.80352 | 2.3704 |
| H | 1.1301 | -0.22199 | -0.62526 | 2.71076 | -1.71221 | 0.61461 | -2.96089 | 1.24332 | 0.59618 | -2.96127 | 0.02521 | 1.24865 | 1.06372 | -0.32446 | -0.60113 | 1.08302 | 0.00693 | -0.76931 |
| H | 2.40264 | 1.34966 | 1.65464 | 1.44183 | -2.68166 | -1.26247 | -2.00719 | 2.6062 | -1.1302 | -2.90205 | 1.56598 | -0.55546 | 2.2306 | 1.74342 | 1.29545 | 1.80495 | 2.50969 | 0.80805 |
| H | 2.39397 | 1.76976 | -0.04979 | 0.76527 | -3.07817 | 0.30431 | -1.26084 | 2.987 | 0.40294 | -2.04611 | 2.26914 | 0.79349 | 1.90723 | 1.91923 | -0.42156 | 1.32958 | 2.40575 | -0.8765 |
| H | 5.51996 | -0.94983 | -0.16304 | 4.70846 | 1.63683 | -1.71919 | -2.37927 | -2.4868 | -1.85076 | -2.4448 | -1.98788 | -2.66613 | 5.51777 | -0.22499 | -0.47037 | 5.33231 | 0.57735 | -1.06604 |
| H | 4.72226 | -2.47782 | -0.5187 | 3.54171 | 0.74187 | -2.73679 | -4.14889 | -2.34738 | -1.8984 | -2.31739 | -3.21903 | -1.41462 | 5.00238 | -1.90785 | -0.45861 | 5.40195 | -0.7865 | 0.04679 |
| H | 5.31325 | -1.45329 | -1.85787 | 2.99527 | 2.10443 | -1.76447 | -3.11749 | -1.23002 | -2.83985 | -3.91351 | -2.82982 | -2.1149 | 5.28868 | -1.0791 | -2.01537 | 5.37868 | -1.09417 | -1.70206 |
| H | -2.39484 | 2.76176 | -0.87485 | -3.41026 | -1.11654 | 0.55785 | 3.40941 | 1.66172 | -0.2205 | 2.57499 | 2.57914 | -0.82728 | -2.98407 | 2.8184 | -0.25632 | -3.61356 | 2.15927 | -0.29378 |
| H | -2.09756 | 3.06982 | 0.83425 | -3.64186 | -1.49731 | -1.13965 | 2.80323 | 1.87491 | -1.85804 | 1.60669 | 2.39965 | -2.28487 | -2.38508 | 2.46386 | 1.35585 | -2.81998 | 2.10691 | 1.2717 |
| H | -2.96635 | -1.42353 | 1.69902 | -1.01607 | 2.39525 | -1.99634 | 1.32543 | -2.52132 | -1.84512 | 1.90652 | -2.20769 | -1.86552 | -2.76302 | -1.74224 | 1.79236 | -2.12581 | -3.62829 | 1.42203 |
| H | -3.09966 | -3.03328 | 0.99015 | -0.49417 | 3.87536 | -1.19509 | 0.9813 | -3.91497 | -0.82294 | 2.27914 | -3.52928 | -0.76068 | -3.05208 | -3.27966 | 0.97094 | -3.44965 | -2.5414 | 1.00248 |
| H | -4.31532 | -1.79857 | 0.63396 | -2.21221 | 3.45391 | -1.23986 | 2.64439 | -3.33521 | -1.00161 | 3.5565 | -2.41704 | -1.27648 | -4.04791 | -1.87629 | 0.58476 | -2.16273 | -1.9902 | 2.08332 |
| H | -2.42325 | -2.02763 | -2.56639 | -0.54699 | 4.11976 | 1.29115 | 1.15378 | -3.79423 | 1.6828 | 4.13006 | -2.00024 | 1.14399 | -1.77977 | -2.26532 | -2.39999 | -1.33684 | -2.69466 | -2.11589 |
| H | -3.01193 | -3.33511 | -1.53232 | -2.26994 | 3.72011 | 1.29917 | 2.82369 | -3.23947 | 1.51349 | 2.90977 | -1.46565 | 2.2964 | -2.46523 | -3.59779 | -1.45991 | -1.62207 | -4.04709 | -1.01332 |
| H | -4.04436 | -1.93796 | -1.86416 | -1.15319 | 2.82034 | 2.3238 | 1.6827 | -2.30912 | 2.48037 | 2.8528 | -3.11078 | 1.65395 | -3.4683 | -2.1995 | -1.88359 | -2.95775 | -2.97468 | -1.46876 |
| H | 1.224 | 0.06627 | 3.22306 | 0.79897 | -0.7379 | 3.51975 | -2.54858 | 1.05829 | 2.97467 | -0.38049 | 0.53731 | 3.75691 | 2.62169 | -0.67243 | 2.85248 | 1.5752 | -0.29702 | 3.48912 |
| H | 0.72001 | -1.61336 | 3.42053 | 0.85355 | -2.24653 | 2.60448 | -0.89098 | 0.76467 | 3.52114 | -1.35739 | 1.71487 | 2.88335 | 1.37346 | 0.52582 | 3.1442 | 2.86887 | 0.45485 | 2.54545 |
| H | 2.34126 | -1.22857 | 2.8245 | 2.34872 | -1.39923 | 2.97359 | -1.28023 | 2.21007 | 2.58895 | -2.11146 | 0.26257 | 3.51443 | 1.10121 | -1.15297 | 3.61918 | 1.38033 | 1.33393 | 2.84191 |
| H | -0.40868 | 1.29805 | -2.09336 | -1.14016 | -3.96335 | 0.55098 | 0.5667 | 3.25473 | 1.40523 | 1.32103 | 3.80811 | 0.85724 | -1.20748 | 1.81601 | -2.17405 | -1.99817 | 3.13664 | -2.20928 |
| H | -0.25219 | 3.0472 | -2.24673 | -1.81781 | -2.76855 | 1.67589 | 1.52354 | 1.80961 | 1.7543 | -0.25052 | 3.21463 | 1.39131 | -0.95933 | 3.53782 | -1.92338 | -0.358 | 2.4833 | -2.21111 |
| H | 1.13978 | 2.07196 | -1.75972 | -2.8756 | -3.65334 | 0.57054 | 2.31955 | 3.29676 | 1.21958 | 1.17572 | 2.17855 | 1.53174 | 0.42582 | 2.44213 | -1.87815 | -1.76129 | 1.39709 | -2.29705 |

## Table S33. Optimized cartesian coordinates of conformers of (1*R,*2*R,*4*R,*5*R,*10*S)*-3 at B3LYP/6-311G(d) level in chlo*ro*for*m*.

|  | (1*R,*2*R,*4*R,*5*R,*10*S)*-3-1 | | | (1*R,*2*R,*4*R,*5*R,*10*S)*-3-2 | | |
| --- | --- | --- | --- | --- | --- | --- |
| C | -0.36083 | 1.89818 | -0.73872 | 1.09827 | 1.77279 | 0.86979 |
| C | 0.8038 | 2.36285 | 0.03445 | 0.55021 | 2.22669 | -0.41693 |
| C | 2.48423 | 0.73717 | -0.93688 | -1.85471 | 1.53771 | 0.01224 |
| C | 2.69315 | -0.2975 | 0.07632 | -2.12253 | 0.33674 | -0.78115 |
| C | 2.33419 | -1.7681 | -0.11251 | -2.52561 | -1.02627 | -0.22912 |
| C | 0.79019 | -1.91988 | -0.02924 | -1.24355 | -1.80651 | 0.18108 |
| C | 0.14464 | -1.33697 | 1.19482 | -0.63741 | -1.41415 | 1.50021 |
| C | -0.93397 | -0.54928 | 1.28143 | 0.57625 | -0.97089 | 1.85053 |
| C | -1.7045 | -0.0732 | 0.07232 | 1.7704 | -0.67534 | 0.97165 |
| C | -1.6786 | 1.44314 | -0.16297 | 2.23088 | 0.79289 | 1.0371 |
| O | -3.09197 | -0.47979 | 0.28872 | 1.50251 | -1.02615 | -0.40633 |
| C | -3.8214 | -0.78237 | -0.80424 | 2.54477 | -1.45683 | -1.14892 |
| O | -3.39436 | -0.73202 | -1.93398 | 3.67484 | -1.54589 | -0.73216 |
| C | -5.22067 | -1.18115 | -0.41779 | 2.10832 | -1.78208 | -2.55185 |
| C | 2.19231 | 2.1764 | -0.57312 | -0.91963 | 2.63211 | -0.45951 |
| O | 3.80974 | 0.24299 | -0.65553 | -3.09179 | 1.40127 | -0.71696 |
| C | 2.79598 | -2.291 | -1.47892 | -3.52003 | -0.90045 | 0.93144 |
| C | 3.03257 | -2.58192 | 0.98949 | -3.20344 | -1.79379 | -1.37827 |
| C | -1.48382 | -0.13208 | 2.62182 | 0.88908 | -0.73264 | 3.31284 |
| O | -0.05025 | 3.30037 | -0.66563 | 1.40661 | 3.08832 | 0.37439 |
| C | 0.77338 | 2.48512 | 1.53783 | 1.13685 | 1.80662 | -1.74132 |
| H | -0.14929 | 1.47229 | -1.72004 | 0.40832 | 1.79734 | 1.7118 |
| H | 2.67875 | 0.03288 | 1.11392 | -1.6289 | 0.2801 | -1.75182 |
| H | 2.11243 | 0.41506 | -1.90645 | -1.98448 | 1.46267 | 1.08822 |
| H | 0.57099 | -2.99574 | -0.05862 | -0.52121 | -1.74777 | -0.62732 |
| H | 0.36096 | -1.49851 | -0.93938 | -1.53388 | -2.86254 | 0.26545 |
| H | 0.60342 | -1.63084 | 2.1372 | -1.31472 | -1.57252 | 2.33753 |
| H | -1.36854 | -0.57303 | -0.83225 | 2.60671 | -1.2965 | 1.30081 |
| H | -2.46137 | 1.68554 | -0.88736 | 2.7121 | 0.96714 | 2.00359 |
| H | -1.91587 | 1.98415 | 0.7527 | 3.00214 | 0.95064 | 0.28377 |
| H | -5.71479 | -0.35211 | 0.09366 | 1.14261 | -2.28819 | -2.55782 |
| H | -5.78539 | -1.45242 | -1.30691 | 2.00193 | -0.85443 | -3.11999 |
| H | -5.19346 | -2.02273 | 0.27722 | 2.86226 | -2.40037 | -3.03463 |
| H | 2.95728 | 2.51373 | 0.13104 | -1.20377 | 2.90692 | -1.4788 |
| H | 2.27021 | 2.80709 | -1.46214 | -1.0526 | 3.52091 | 0.16265 |
| H | 3.87537 | -2.1886 | -1.59292 | -3.13086 | -0.30395 | 1.75788 |
| H | 2.31864 | -1.75908 | -2.30543 | -3.76246 | -1.89128 | 1.32561 |
| H | 2.53803 | -3.34853 | -1.57782 | -4.44474 | -0.43232 | 0.59318 |
| H | 2.74662 | -2.25025 | 1.99057 | -4.05719 | -1.23433 | -1.76971 |
| H | 2.78689 | -3.64383 | 0.90412 | -2.50616 | -1.96701 | -2.20367 |
| H | 4.11808 | -2.48072 | 0.90846 | -3.56533 | -2.76703 | -1.03619 |
| H | -2.48513 | -0.54376 | 2.77212 | 0.07021 | -1.06956 | 3.94874 |
| H | -0.84457 | -0.48055 | 3.43432 | 1.79537 | -1.26734 | 3.61718 |
| H | -1.57614 | 0.9533 | 2.71195 | 1.06391 | 0.32554 | 3.53227 |
| H | 1.41218 | 3.31563 | 1.851 | 1.05693 | 2.63306 | -2.45333 |
| H | -0.23107 | 2.67838 | 1.90898 | 2.18779 | 1.53571 | -1.65665 |
| H | 1.14643 | 1.57365 | 2.00883 | 0.58976 | 0.95522 | -2.14907 |


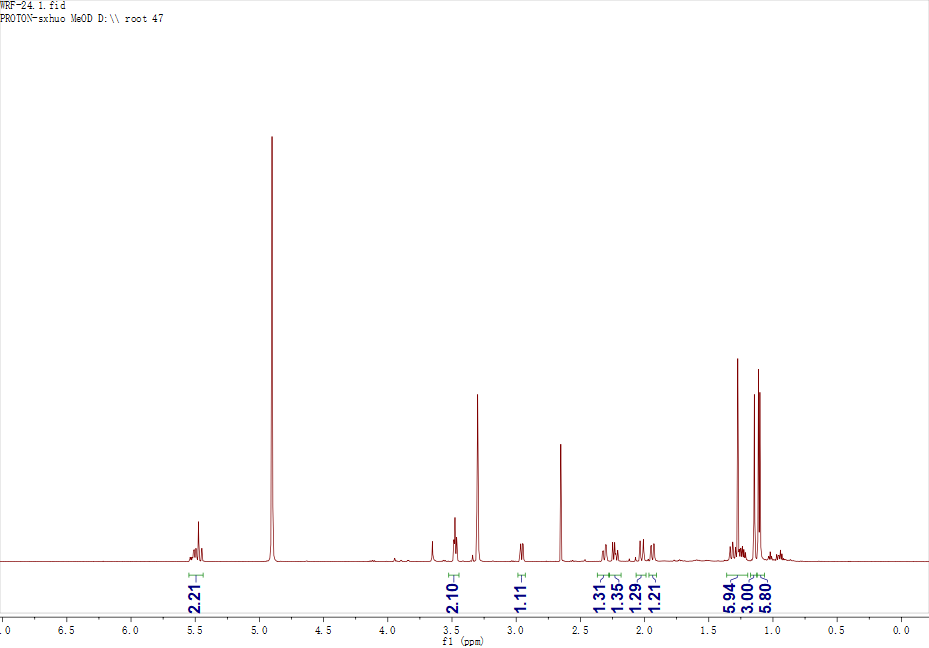


## Figure S4. ^1^H NMR (500 MHz) spectrum of compound 1 in MeOD


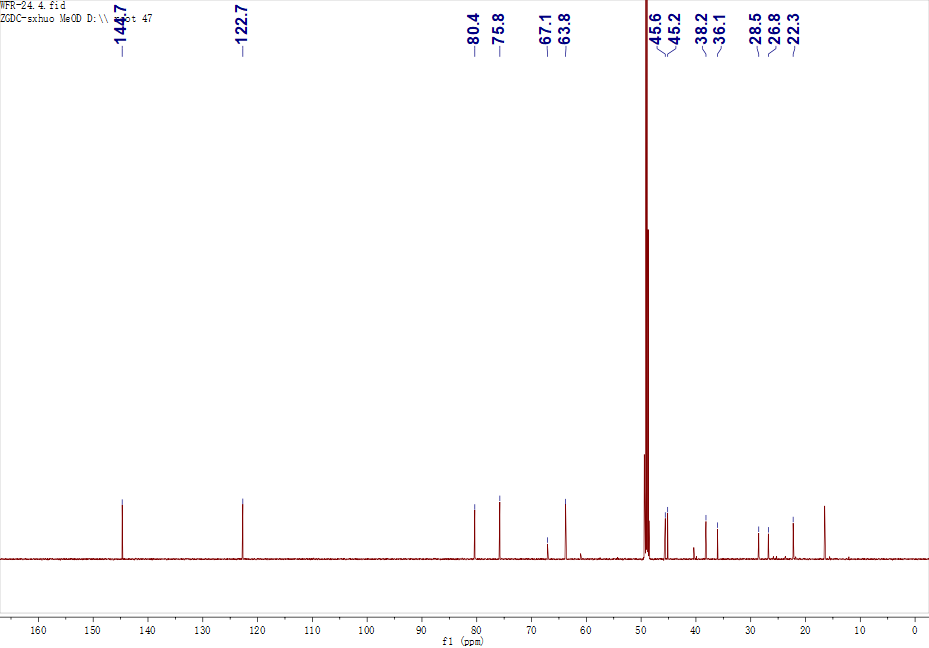


## Figure S5. ^13^C NMR (125 MHz) spectrum of compound 1 in MeOD


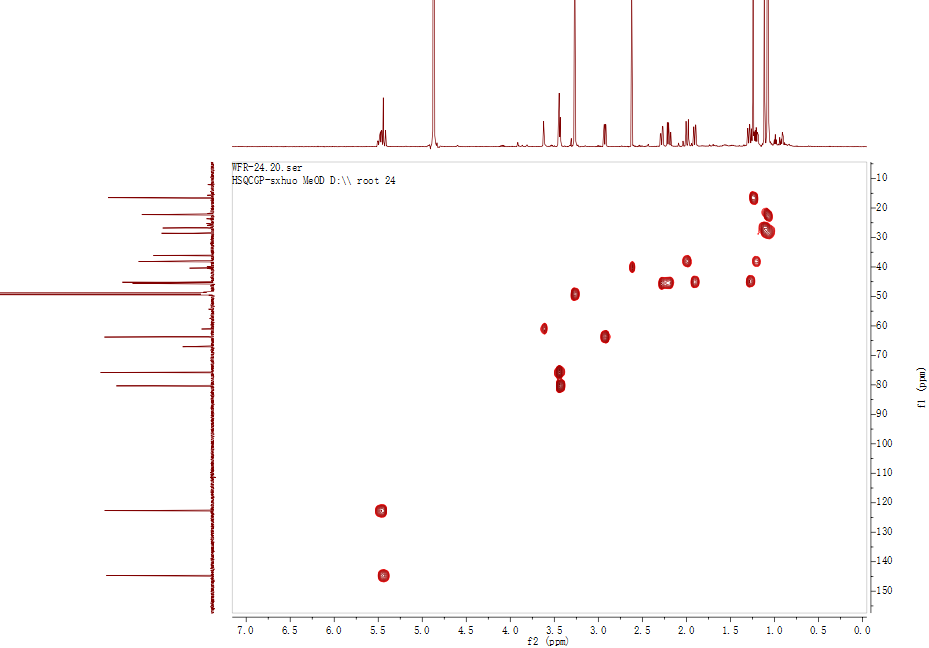


## Figure S6. HSQC spectrum of compound 1 in MeOD

##
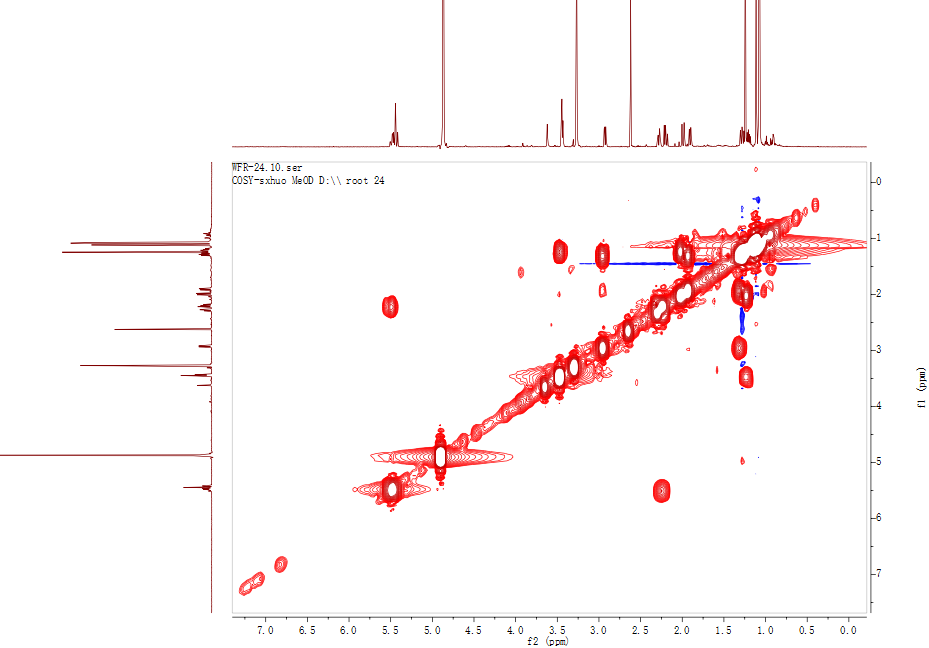


## Figure S7. ^1^H-^1^H COSYspectrum of compound 1 in MeOD


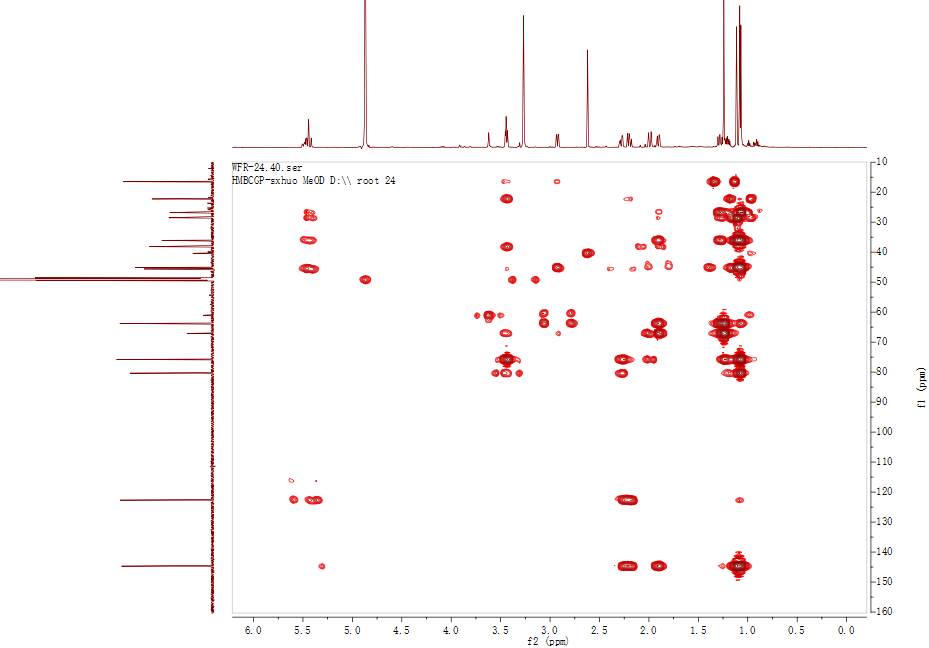


## Figure S8. HMBC spectrum of compound 1 in MeOD


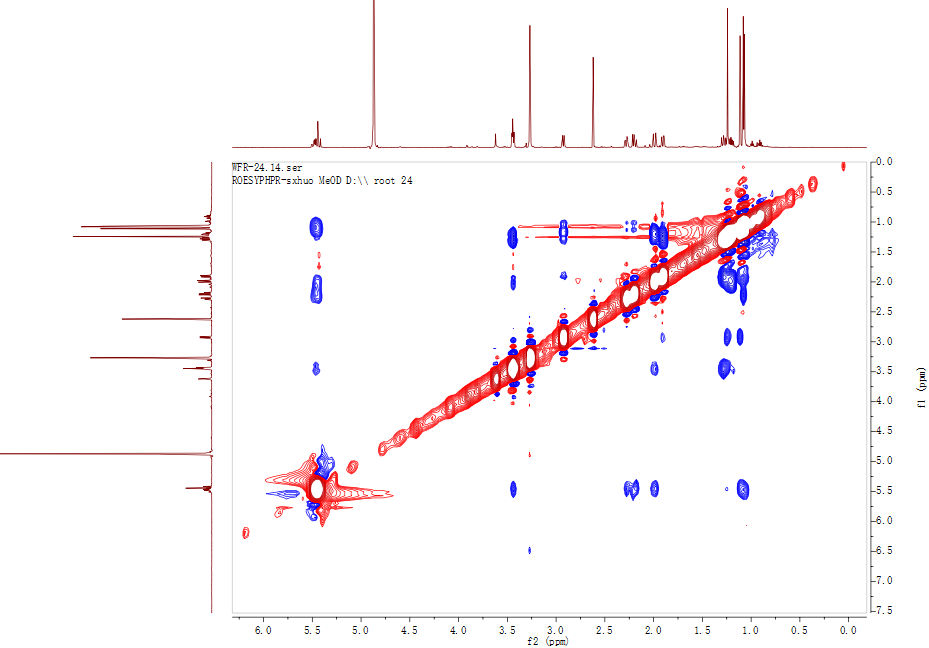


## Figure S9. ROESY spectrum of compound 1 in MeOD


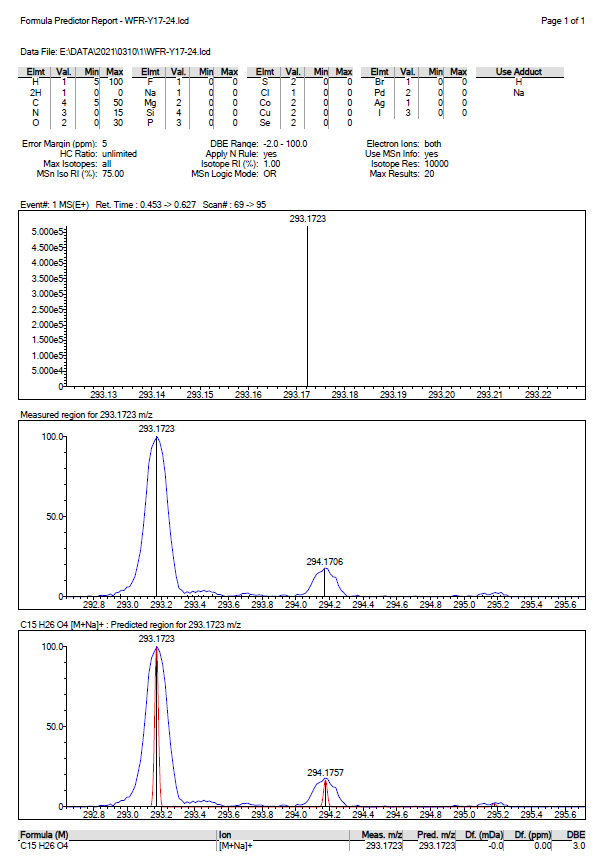


## Figure S10. HRESIMS of compound 1


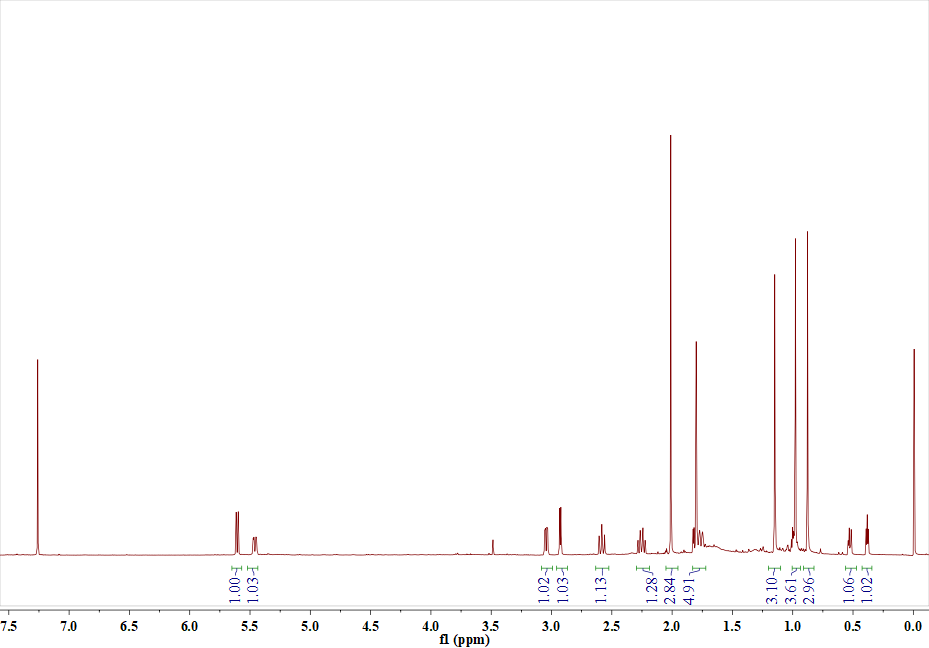


## Figure S11. ^1^H NMR (500 MHz) spectrum of compound 2 in CDCl_3_


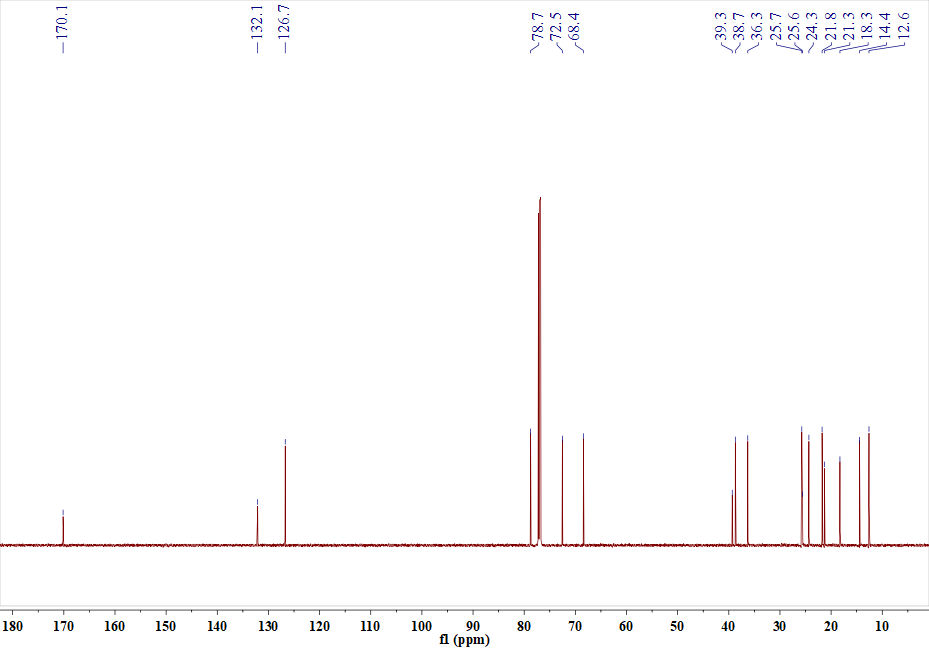


## Figure S12. ^13^C NMR (125 MHz) Spectrum of compound 2 in CDCl_3_


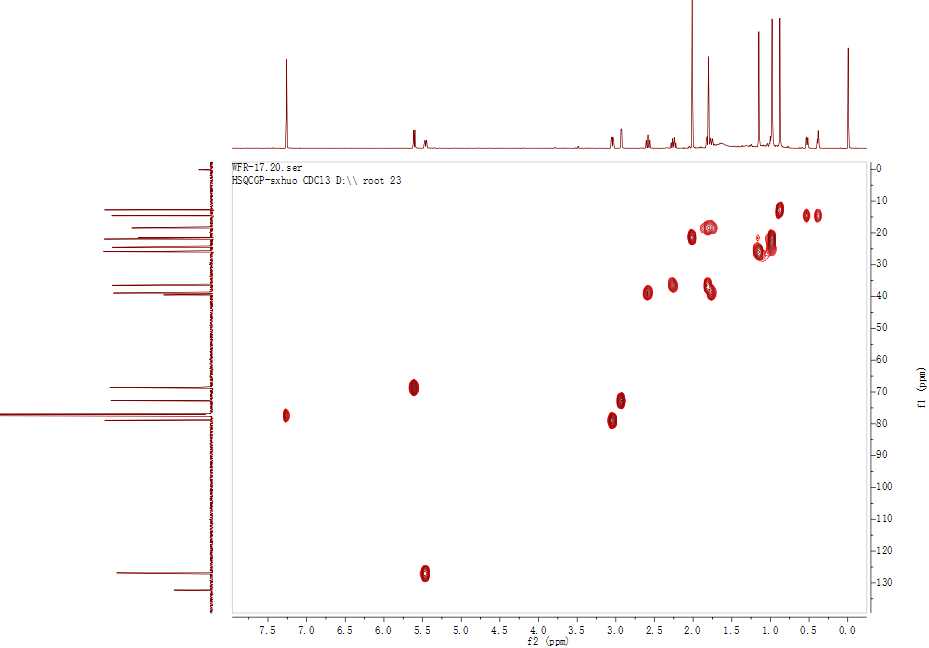


## Figure S13. HSQC spectrum of compound 2 in CDCl_3_


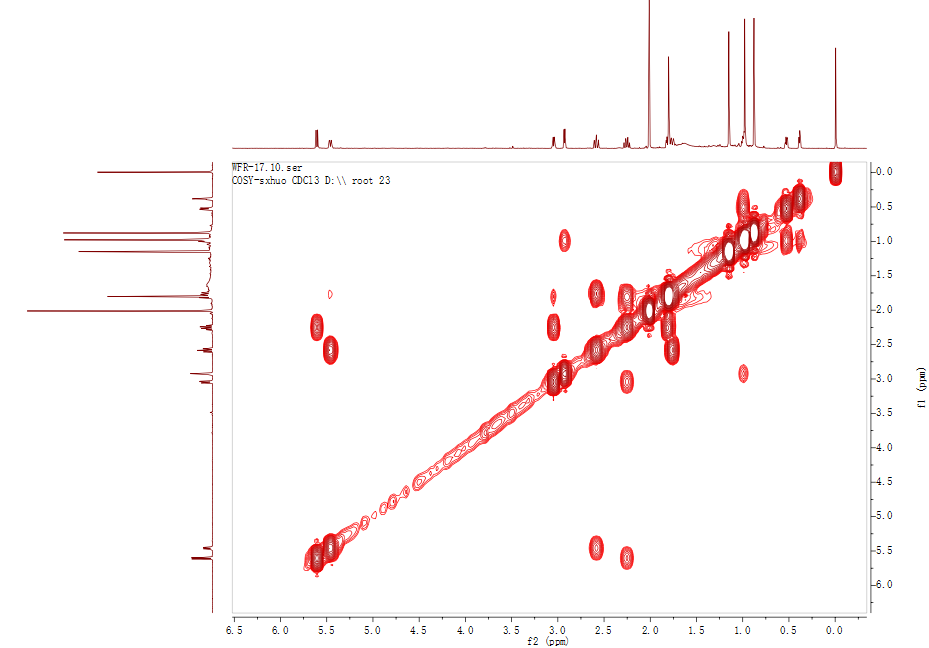


## Figure S14. ^1^H-^1^H COSY spectrum of compound 2 in CDCl_3_


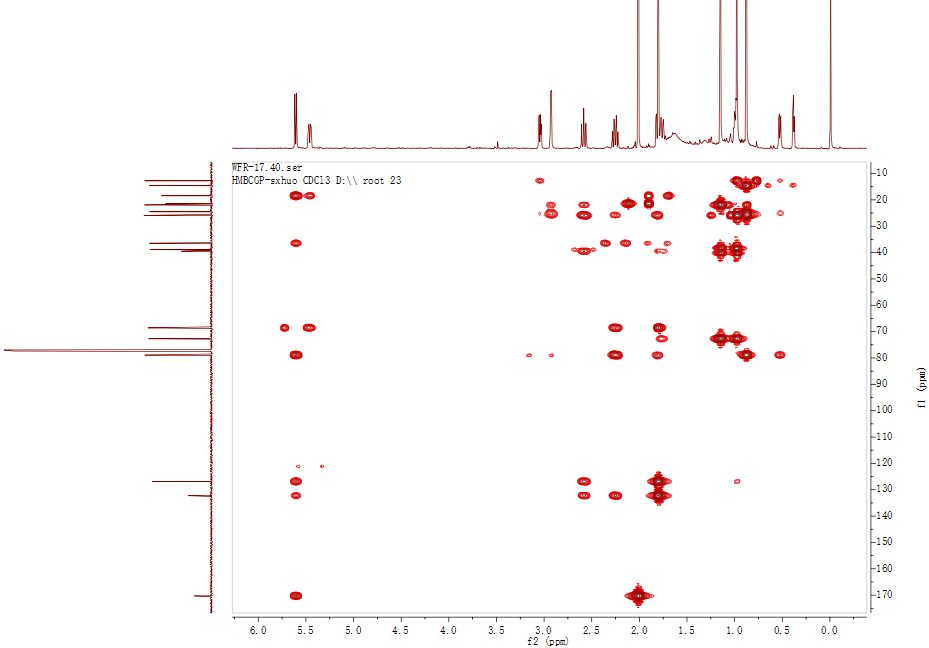


## Figure S15. HMBC spectrum of compound 2 in CDCl_3_


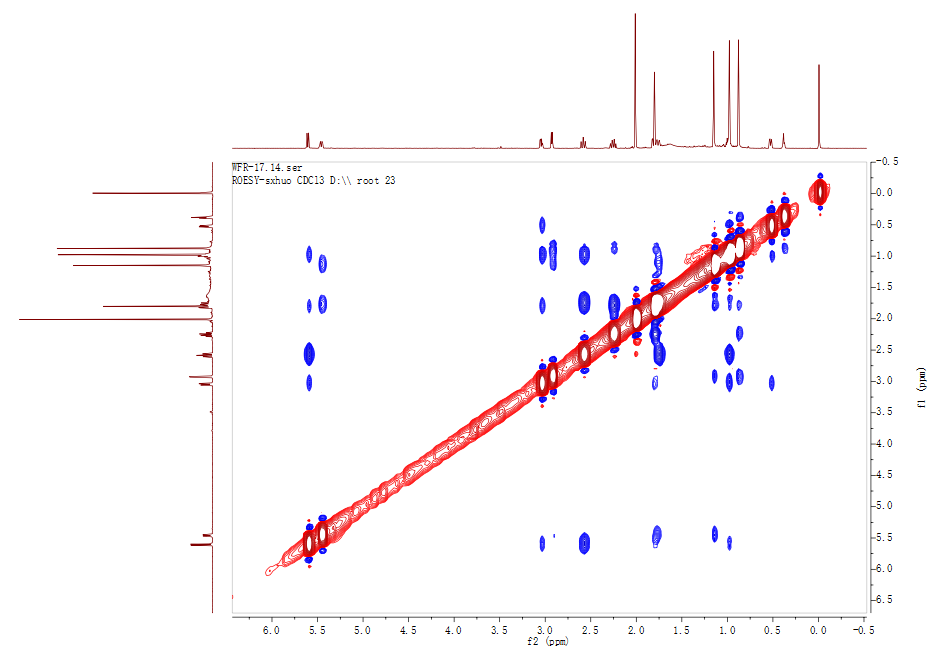


## Figure S16. ROESY spectrum of compound 2 in CDCl_3_


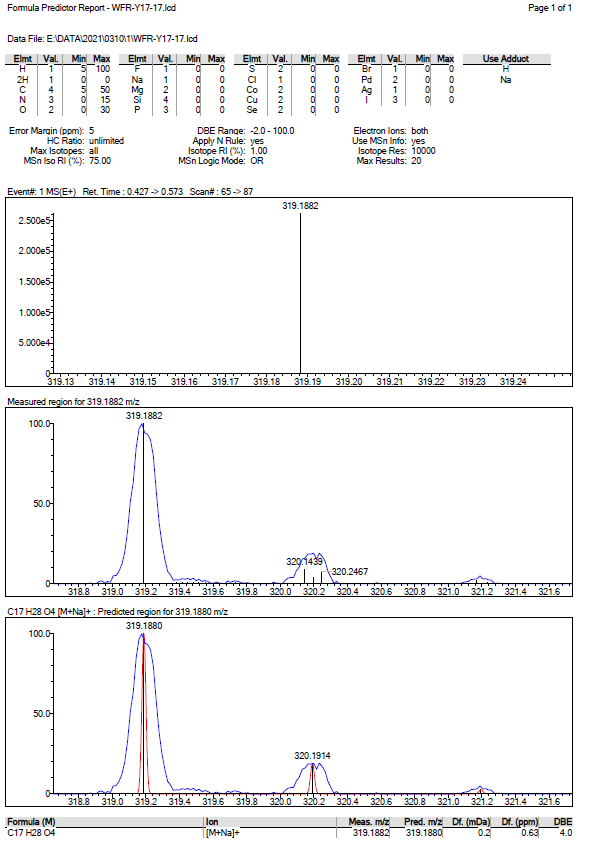


## Figure S17. HRESIMS of compound 2


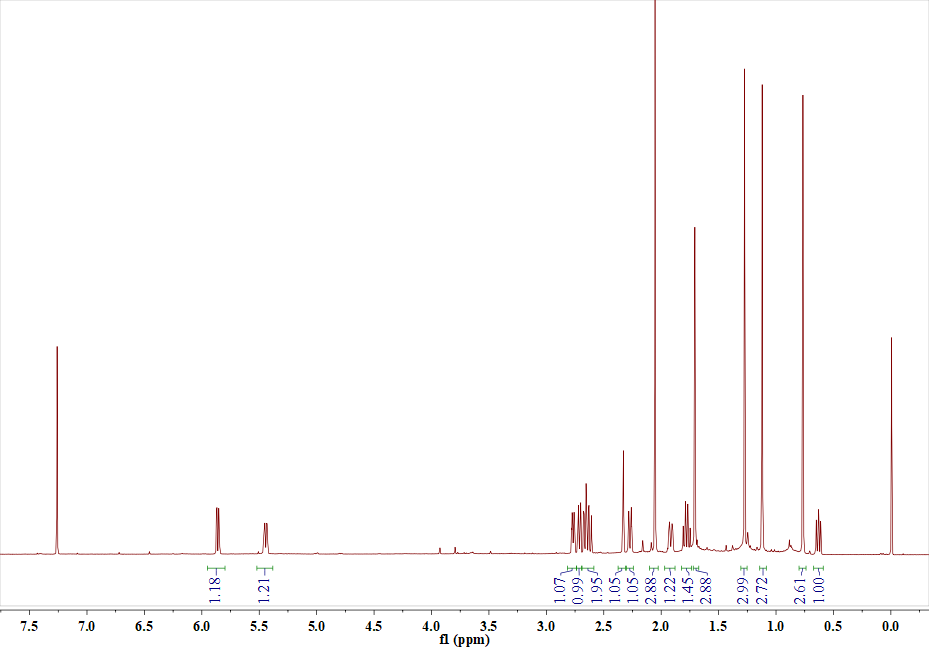


## Figure S18. ^1^H NMR (500 MHz) spectrum of compound 3 in CDCl_3_


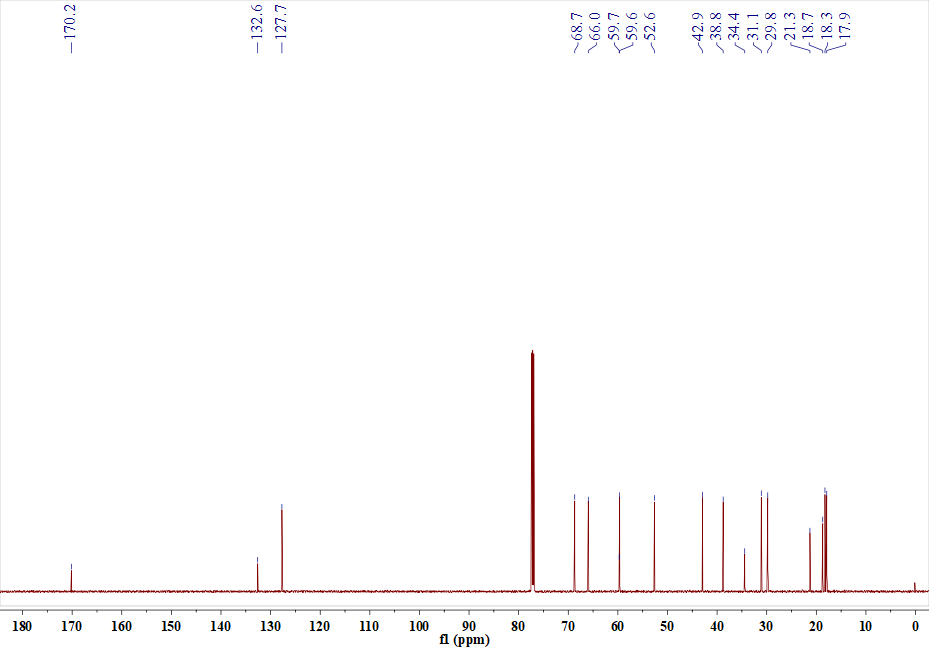


## Figure S19. ^13^C NMR (125 MHz) spectrum of compound 3 in CDCl_3_


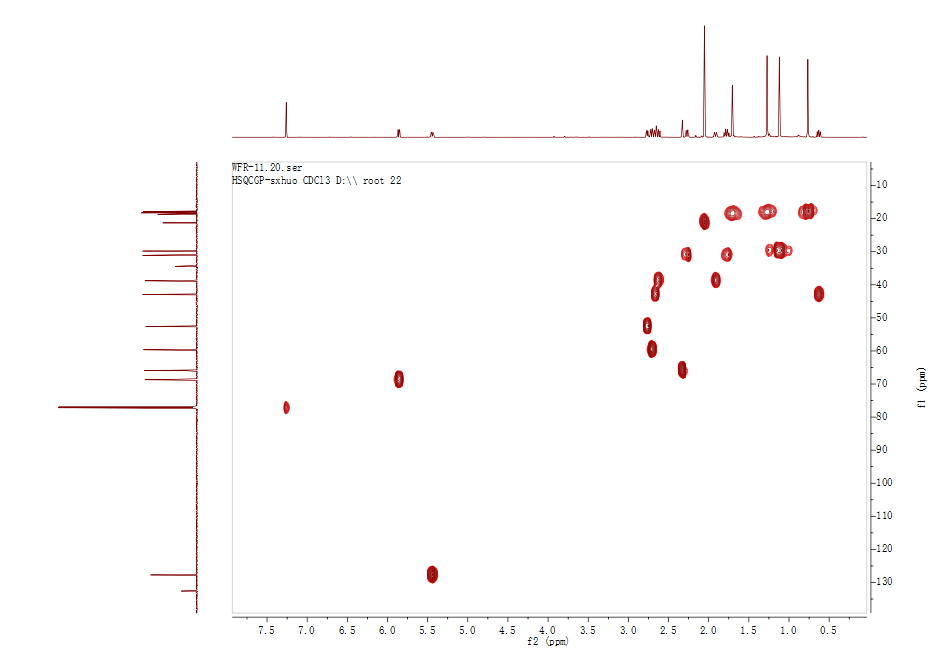


## Figure S20. HSQC spectrum of compound 3 in CDCl_3_


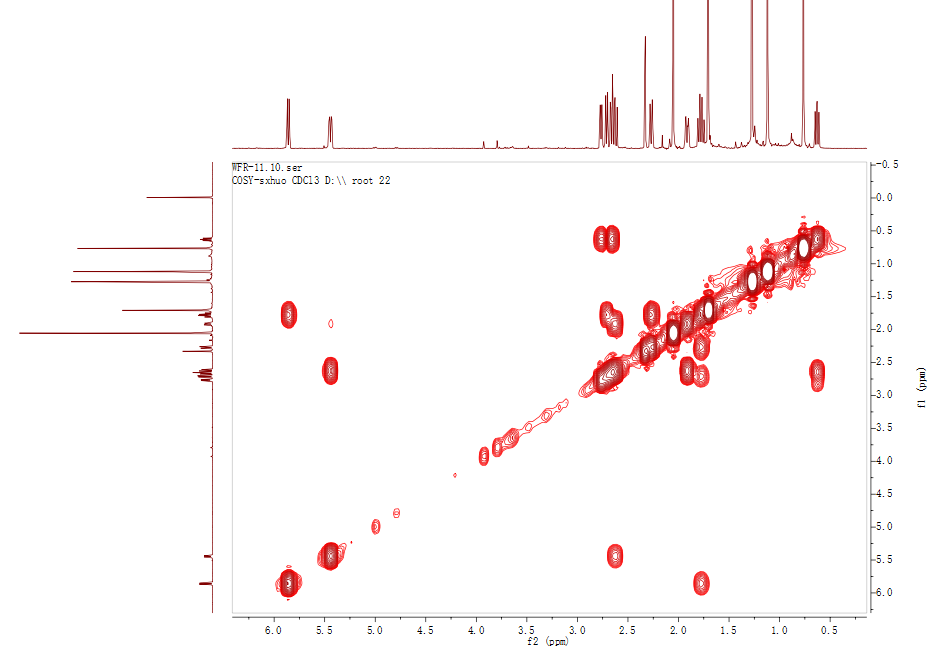


## Figure S21. ^1^H-^1^H COSY spectrum of compound 3 in CDCl_3_


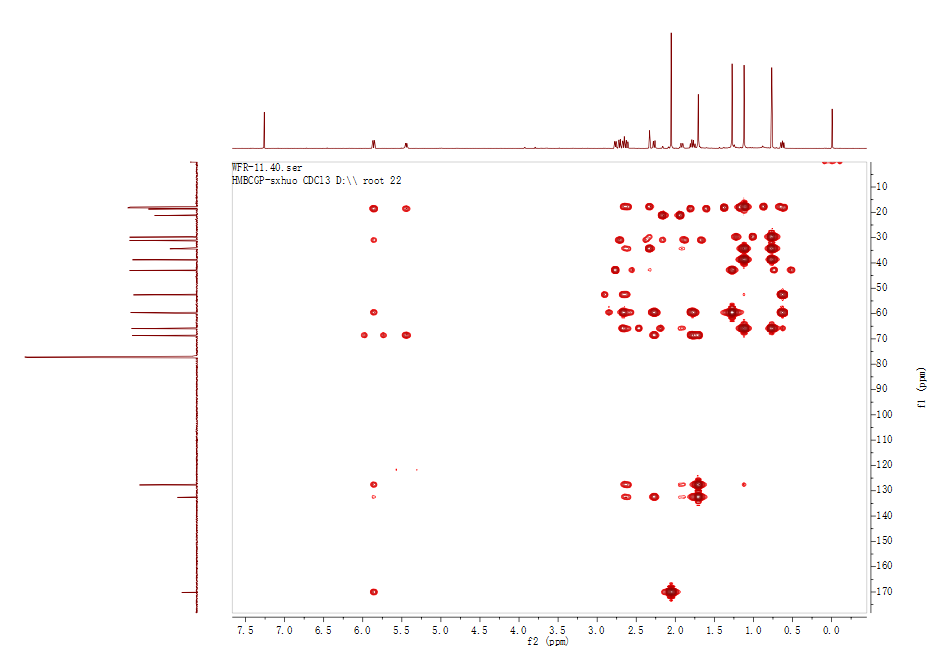


## Figure S22. HMBC spectrum of compound 3 in CDCl_3_


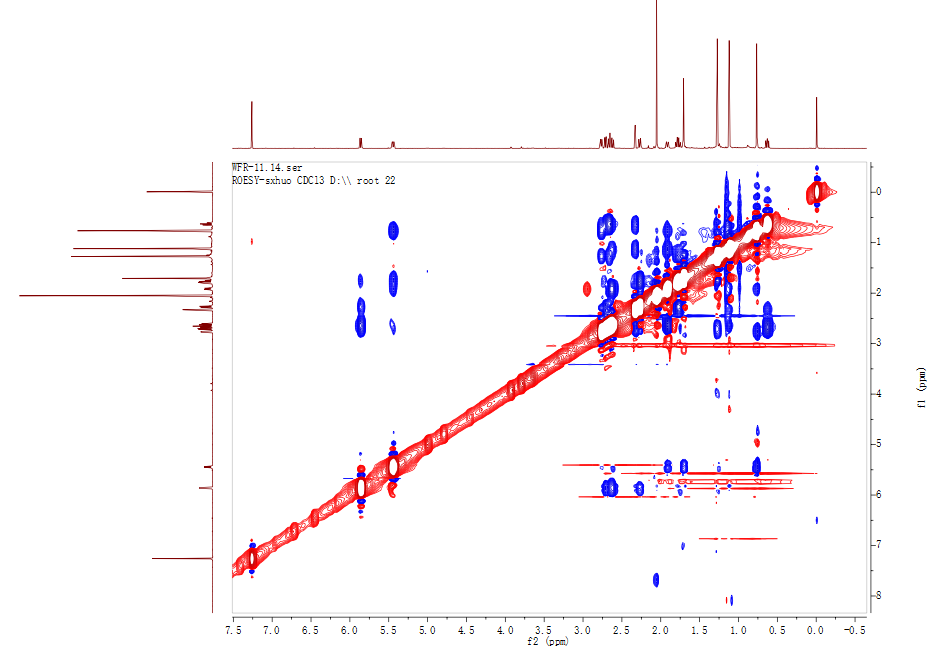


## Figure S23. ROESY spectrum of compound 3 in CDCl_3_


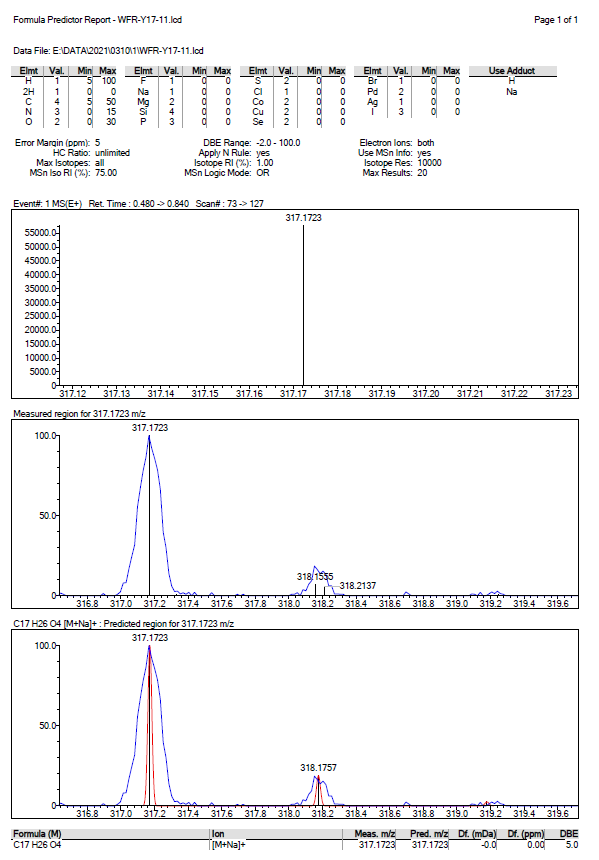


## Figure S24. HRESIMS of compound 3


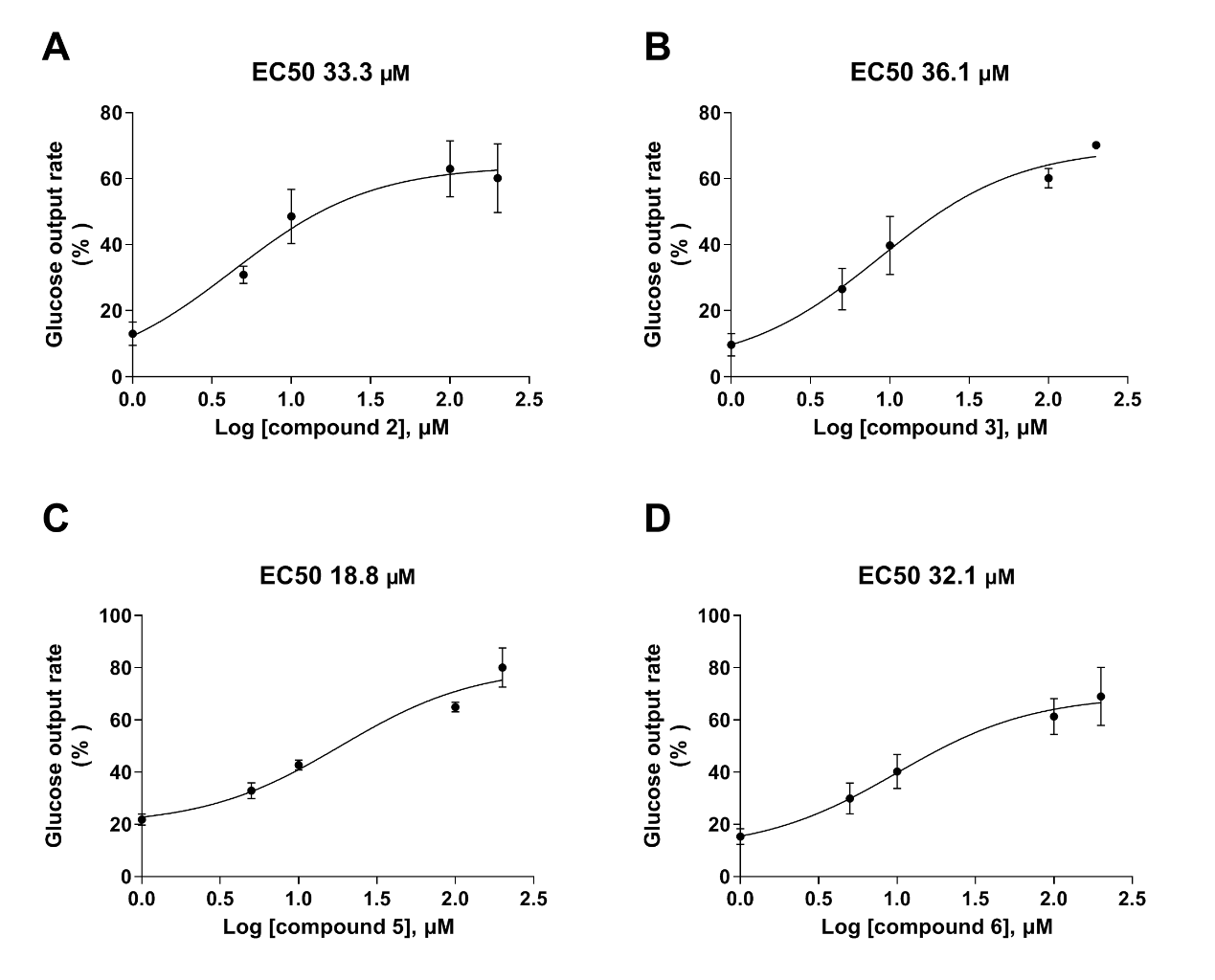


## Figure S25. Dose-response-curves of compounds 2, 3, 5, and 6 against glucagon-induced hepatic glucose production

## Figure S26. Phylogenetic tree of *Penicillium* sp.
